# Thermodynamics of Anion Binding by (Thio)ureido-calix[4]arene Derivatives in Acetonitrile

**DOI:** 10.1021/acsphyschemau.4c00077

**Published:** 2024-10-15

**Authors:** Marija Cvetnić, Nikola Cindro, Nikola Bregović, Vladislav Tomišić

**Affiliations:** Department of Chemistry, Faculty of Science, University of Zagreb, Horvatovac 102a, 10000 Zagreb, Croatia

**Keywords:** calixarenes, urea and thiourea derivatives, anion binding, thermodynamics, supramolecular chemistry

## Abstract

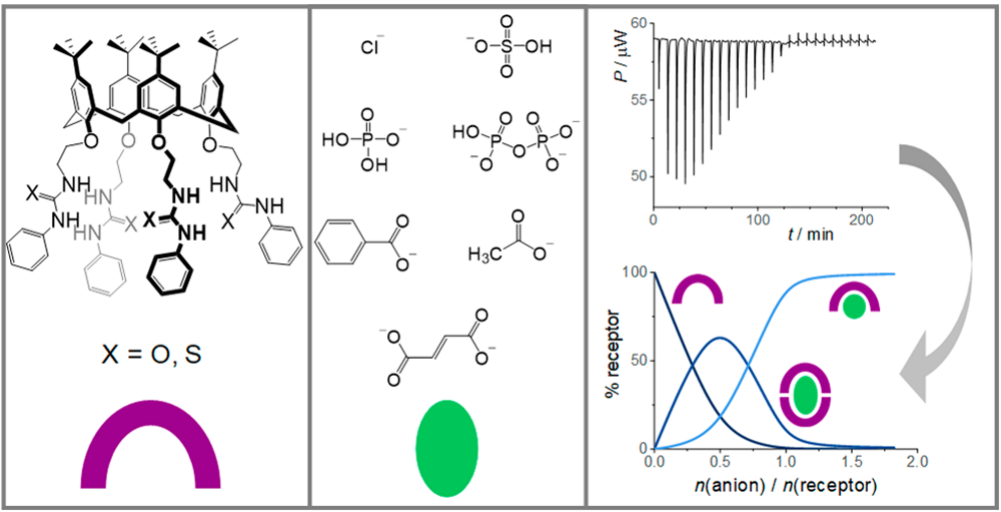

In this work, we developed (thio)ureido-calix[4]arene derivatives and thoroughly explored their anion-binding properties in acetonitrile. A series of anions, including important inorganic ones (Cl^–^, HSO_4_^–^, H_2_PO_4_^–^, and HP_2_O_7_^3–^) and several ever-present carboxylates (acetate, benzoate, and fumarate), were studied. All systems were investigated by several methods (NMR, ITC, and UV) used in a synergistic fashion, providing their comprehensive thermodynamic description. Acidities of the receptors were determined prior to the anion-binding studies and considered in the data-handling procedures. Complexes of various stoichiometries were detected and the driving force for their formation elucidated. The correlation of the anion structural features and H-bond acceptor properties with the stoichiometries and complexation thermodynamics parameters was rationalized. Generally, stability of the complexes followed the trend defined by the basicity of anions. Thiourea and urea analogues exhibited similar affinities for anion binding except for the H_2_PO_4_^–^ and HP_2_O_7_^3–^, which interacted with the thiourea analogue more strongly. The hosts endowed with 4 (thio)urea groups formed species containing two receptor molecules bridged by a fumarate or hydrogen pyrophosphate anion. Thermodynamic information provided in this work is applicable in further design of supramolecular systems, whereas the presented approach to data handling will aid researchers when dealing with multiple coexisting equilibria.

## Introduction

Anion recognition in solution is an important and continuously growing^1−3^ research field within host–guest chemistry due to many potential areas of its application: biochemical transmembrane transport (leading to anticancer or antibacterial activity),^4^ extractions (applicable also on industrial level),^5,6^ catalysis,^7^ multipotent supramolecular capsular assemblies,^8^ water purification systems,^9,10^ sensing,^11,12^ etc.^13^ Functional groups that enable synthetic molecular hosts’ binding of anions are (a) hydroxyl groups, amines, (thio)amides, (thio)squaramides, and (thio)ureas (hydrogen bonding);^14−18^ (b) metal cations (direct Coulombic interaction with anions^19^ or influence on the binding of anions through the second coordination sphere);^20^ (c) neutral and cationic CH hydrogen-bond donors;^21,22^ (d) electron-deficient triazine and tetrazine rings (anion–π interaction);^23^ (e) haloperfluoroarenes, haloimidazolium, and halotriazole/triazolium moieties (halogen bonding);^24,25^ (f) chalcogenodiazoles, chalcogenophenes, and nonaromatic ChB-donor groups (chalcogen bonding);^26^ and (g) rarely used moieties containing tetrel and pnictogen donor atoms (σ–hole interactions).^27^ Despite a tremendous variety in approaches of achieving anion recognition, most anion receptors still rely on hydrogen bonding, whereby the NH groups are probably the most common H-bond donors.^14,16,28^ Among the latter, (thio)urea compounds stand out as particularly interesting hosts for anions due to the ease of attaching this functional group to a selected molecular backbone and the potential for creating stronger/more hydrogen bonds with anions in comparison to amide moieties present in peptides. It is worth mentioning that one of the ultimate goals of anion recognition chemistry–selective anion sensing in water was recently achieved using urea-based cryptand.^29^ Multiple urea moieties within a linear molecule recently enabled interconversion of single–double helix structures with simultaneous helical sense switching, regulated by anion coordination.^30^ On the other hand, placing urea groups on a more complex scaffold, polyhedral oligomeric silsesquioxane, was used to obtain a fluorescence sensor for quantifying sulfate ions.^31^ Furthermore, a remarkable application of a novel macrocyclic bis-thiourea derivative was found in the catalysis of stereospecific invertive substitution pathways of glycosyl chlorides.^32^

Among macrocyclic scaffolds calixarenes stand out with their versatility and applicability.^33−38^ Although they were primarily investigated as receptors for cations,^39−42^ the exploration of their anion-binding potential started by introducing (thio)urea moieties on their rims (usually calix[4]arenes and calix[6]arenes).^43,44^ Despite being developed 30 years ago in Reinhoudt’s group, the synthesis of new derivatives in this class of molecules, as well as the research of their binding properties, is still vibrant. Namely, they are explored for chiral recognition,^45^ as receptors in ion-selective electrodes,^46−48^ as neoglycoconjugates in site-specific molecular delivery systems,^49^ for the extraction of toxic oxyanions, e.g., of Cr(VI) and As(V),^50^ in the preparation of Langmuir–Blodgett thin films for the sensing of hazardous volatile organic compounds,^51^ as binders and transporters of different biochemically relevant anion and ion-pair species,^52−56^ and in the construction of pseudorotaxanes conceivably important for the design of linear molecular motors.^57^ Furthermore, (thio)urea functionalities were lately used in the decoration of *p*-*tert*-butyldihomooxacalix[4]arenes.^58−62^ In the investigations of the anion-binding behavior of the latter macrocycles, several parameters were tested: number of (thio)urea moieties, size of linker for (thio)ureas (propyl and butyl), position and type of other functionalities at the lower rim of calixarene, and conformation of calixarene. Generally, better affinity for the anions was established for dihomooxacalix[4]arenes with a shorter linker and phenyl (instead of alkyl) group at (thio)urea, as was observed for regular calix[4]arenes.^43,44^ Also, among tetra(thio)urea derivatives of (dihomooxa)calix[4]arenes, the analogues comprising urea moieties were better receptors for the anions. These investigations were done in chloroform, and no ITC measurements exist to reveal a complete thermodynamic picture of anion complexation processes.

Another point that is not often (or comprehensively) covered in the investigations of anion binding in nonaqueous solvents is the characterization of the acidity of the receptors. Namely, besides the binding event, hosts for anions containing a (thio)urea moiety can undergo deprotonation of the NH group.^63−68^ Protonation of the anion on the account of the (thio)urea receptor is often present in aprotic organic solvents of low polarity because of the increased basicity of anions in comparison to their behavior in aqueous solutions (e.g., p*K*_a_(AcOH, MeCN) = 22.23; p*K*_a_(BzOH, MeCN) = 21.5).^69^ However, due to the need of highly precise and reliable titration data, as well as because of the difficulties in data analysis, very few examples exist where proton transfer was included in the modeling of the host–guest complexation process.^67^

During recent years, studies of anion-binding abilities of various receptors with particular attention to the thermodynamics of the related processes have been the focus of our group. This research included aromatic bis-urea derivatives,^67^ dehydroacetic derivatives with amide and urea moieties,^70^ and aromatic sulfonylureas.^68^ In the first two classes of receptors, good sensitivity and selectivity for acetate and dihydrogen phosphate in DMSO and/or MeCN was observed. In all cases, p*K*_a_ values of the explored receptors were experimentally determined and, when required, incorporated into models used for fitting titration data obtained by adding basic anions. The experience we gathered in the chemistry of anion binding and the chemistry of calixarenes led us to the conception of pH-sensitive formation of calixarene heterodimers in MeCN.^71^ This was achieved by coupling urea derivatives of *p*-*tert*-butylcalix[4]arenes with diacetato-calixarenes, which was inspired by high affinity of the ureido-calixarenes toward acetate.

**Scheme 1:**
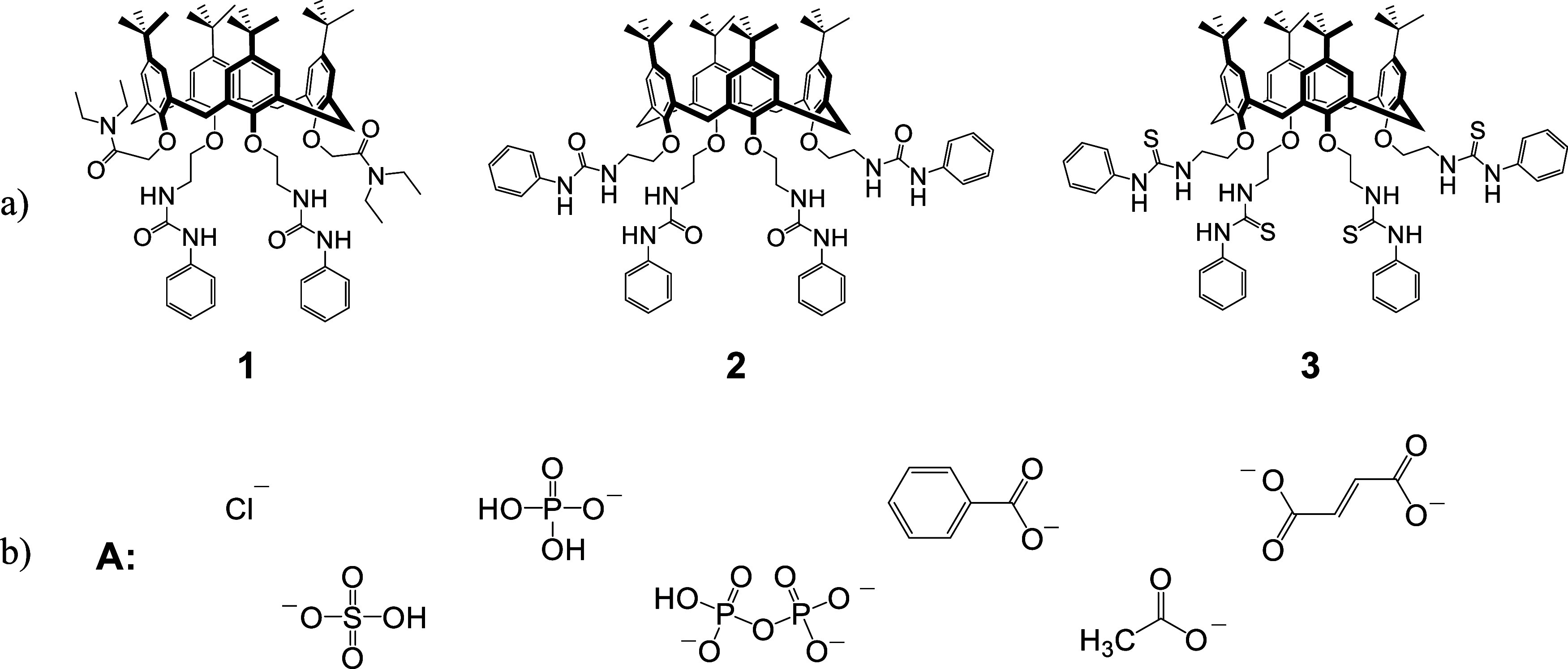
Structures and Assignments of the Investigated (a) Calixarenes and (b) Anions

Herein, we present a detailed thermodynamic study of anion binding by the above-mentioned ureido-calixarenes along with their novel tetrathiourea analogue (Scheme 1). Comprehensive research including a range of different anions was carried out using ^1^H NMR, UV spectrophotometry, and ITC. Furthermore, in the case of basic anions (acetate and benzoate), the extent of proton transfer between the receptor and anion was also addressed. Special attention was also paid to the detection of a variety of complex stoichiometries and their full thermodynamic characterization.

## Results and Discussion

### Synthesis and Characterization of Receptors

The synthesis of urea derivatives of *p*-*tert*-butylcalix[4]arene (**1** and **2**) is described in our recent publication,^71^ whereas the synthesis of the thiourea derivative of *p*-*tert*-butylcalix[4]arene (**3**) was performed using a similar procedure, described in detail in Supporting Information. The NMR, HRMS, and SC-XRD measurements proved the *cone* conformation of calixarene **3** in the acetonitrile solution and in the solid state, as was the case with its urea analogue **2**. The solubility of compound **3** in MeCN is at least 100 times higher than that of **2** (0.075 mmol dm^–3^)^71^ in the same solvent. The latter was concluded from the ^1^H NMR-concentration dependence experiment (Figure S4) which also indicated that no self-assembly of receptor **3** occurred in MeCN. The assignments of the proton signals for the three receptors investigated herein are depicted in Figure S1 (**3**) and Figure S5 (**1** and **2**).

### Acidity of Receptors

Prior to the anion-binding investigations, the acidity of receptors in MeCN had to be explored in order to treat the anion-binding results in the proper manner, i.e., to address the possibility of proton transfer. The proton dissociation of the herein prepared (thio)urea derivatives of calixarenes was characterized by ^1^H NMR (and UV) titrations with suitable organic bases. The selection of the appropriate organic base for each receptor was done by applying the following criteria: low spectral overlap of the base and the receptor, absence of complexation between the base and the receptor, and the convenient p*K*_a_ value of the protonated base (base strong enough to deprotonate urea yet not too strong to do it quantitatively). The organic base that enabled the acidity characterization of macrocycle **1** was phosphazene base P_2_Et,^72^ whereas the dissociation of first two protons of macrocycles **2** and **3** was studied by means of the titration with 1,8-diazabicyclo[5.4.0]undec-7-ene (DBU) (Table 1).

**Table 1:** p*K*_a_ Values for **1**, **2**, and **3** in MeCN at 25 °C Determined by Competitive Titration Experiments with Organic Bases of Known p*K*_a_ Values^72,75,76^^a^

macrocycle (**M**)	referent base (p*K*_a_)	p*K*_a1_**(M/M**^**–**^**, MeCN)**	p*K*_a2_**(M/M**^**2–**^**, MeCN)**
**1**	P_2_Et (32.94)^b^	32.8(3)^d^	31.8(3)^d^
**2**	DBU (24.34)^c^	24.7(7)^d^	25.4(3)^d^
**3**	DBU (24.34)^c^	22.84(4)^d^	24.28(4)^d^
		22.49(7)^e^	24.26(1)^e^

a Standard deviations are given in parentheses.

b P_2_Et = 1-ethyl-2,2,4,4,4-pentakis(dimethylamino)-2λ^5^,4λ^5^-catenadi(phosphazene).

c DBU = 1,8-diazabicyclo[5.4.0]undec-7-ene.

d Determined by NMR.

e Determined by UV.

Addition of P_2_Et to **1** in CD_3_CN caused a downfield shift of urea protons complemented with broadening of the signals and a decrease in their intensity (Figure S6). This was in line with the expected proton dissociation but hindered the quantitative consideration of this signal. Nonetheless, the shifts of other proton signals could be used in the fitting procedure performed within the HYPNMR program^73^ in a multivariate fashion (Figure S6b,c and Table S3). Details of the refinement procedures are provided in the Supporting Information (Section S2). The largest shifts during deprotonation of **1** were observed for aryl and *N*-ethyl (NC**H**_2_CH_2_) protons suggesting that removal of proton(s) significantly impacted the conformation of **1**, probably due to the disruption of intramolecular hydrogen bonds. The deprotonation of the NH_a_ proton in the titration of **1** with P_2_Et is also supported by the change of multiplicity of the linker proton signal (OCH_2_C**H**_2_N) from quartet to triplet (Figure S6) during the titration. The obtained p*K*_a_ values for two most acidic protons of **1** (both around 32; Table 1) suggested that not even typical basic anions in MeCN such as acetate or benzoate are able to deprotonate it. In other words, there is no need to include the proton transfer reaction when fitting data acquired by titrating **1** with the aforementioned anions.

First two p*K*_a_ values of **2** were determined using DBU as a base in the ^1^H NMR titration (Table 1). The most significant changes in chemical shifts were detected for **2***ortho*-phenyl and linker (OCH_2_C**H**_2_N) protons (Figure S7a). The resulting titration curves were processed, allowing the quantitative characterization of the deprotonation processes. During the titration of **2** with DBU (*n*(DBU)/*n*(2) = 0.5–2), broadening of several proton signals was also observed. This is most likely due to intermediate kinetics of the first deprotonation in comparison with the second one, with respect to the NMR time scale. The resulting p*K*_a_ values of **2** were around 25 which implied that no proton transfer between the receptor and anion should be expected if the anion is not used in high excess (Δp*K*_a_ = 2.5 for **2** vs AcOH).

Almost 10 times less DBU was required for achieving saturation of the NMR titration curve in the case of thioureido-calixarene **3** (Figure S8) in comparison to **2** (Figure S7). This observation reflected a much lower p*K*_a_ value of **3** compared to **2** (Table 1). Greater acidity was indeed expected for the calixarene comprising the thiourea moiety in relation to ureido-calixarene. Namely, in one of the first thorough investigations of p*K*_a_ values of compounds in nonaqueous solutions (DMSO), simple thiourea (p*K*_a_ = 21.1) was reported to be 6 orders of magnitude more acidic than urea.^74^ The reason for these findings is better stabilization of negative charge by sulfur than by oxygen. We validated the NMR results using UV titration, which indeed yielded similar p*K*_a_ (Table 1) and provided the characteristic UV spectra of all protonation forms of **3** (Figure S9). The drastic increase in acidity of **2** and **3**, compared to **1** (8 and 10 orders of magnitude, respectively), points out the significance of an array of intramolecular hydrogen bonds formed in the case of receptors with four (thio)urea segments which stabilize their deprotonated forms. When the p*K*_a_ values established for **3** are compared to the acidity of protonated anions of higher basicity, such as benzoate (Δp*K*_a_ = 1.2) and acetate (Δp*K*_a_ = 0.4), proton transfer from the receptor to the anion is expected, especially in solutions containing large amounts of anions, particularly acetate, and will thus be considered in the following sections.

### Anion-Binding Investigations

**Figure 1:**
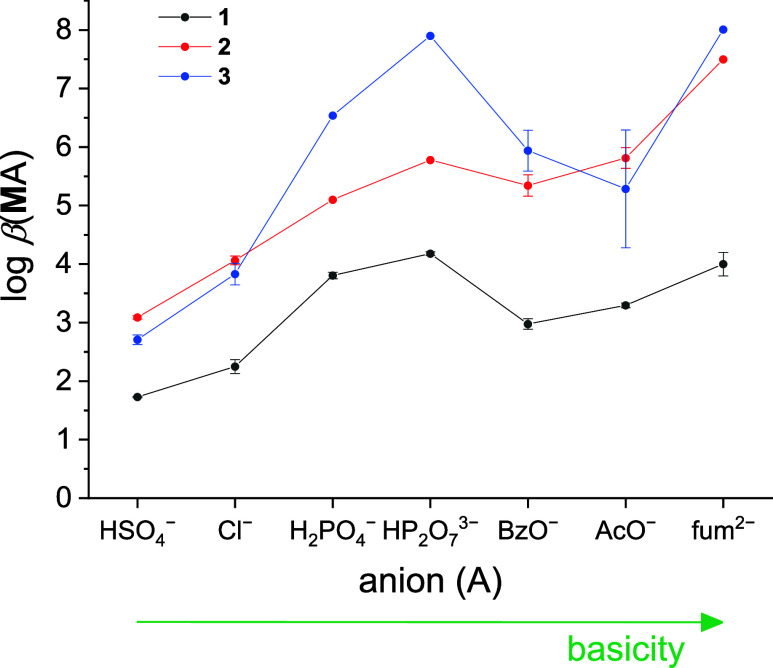
Comparison of the stability constants related to 1:1 complexes of the investigated (thio)ureido-calix[4]arenes (**M**) with selected anions (A) in acetonitrile at 25 °C.

**Figure 2:**
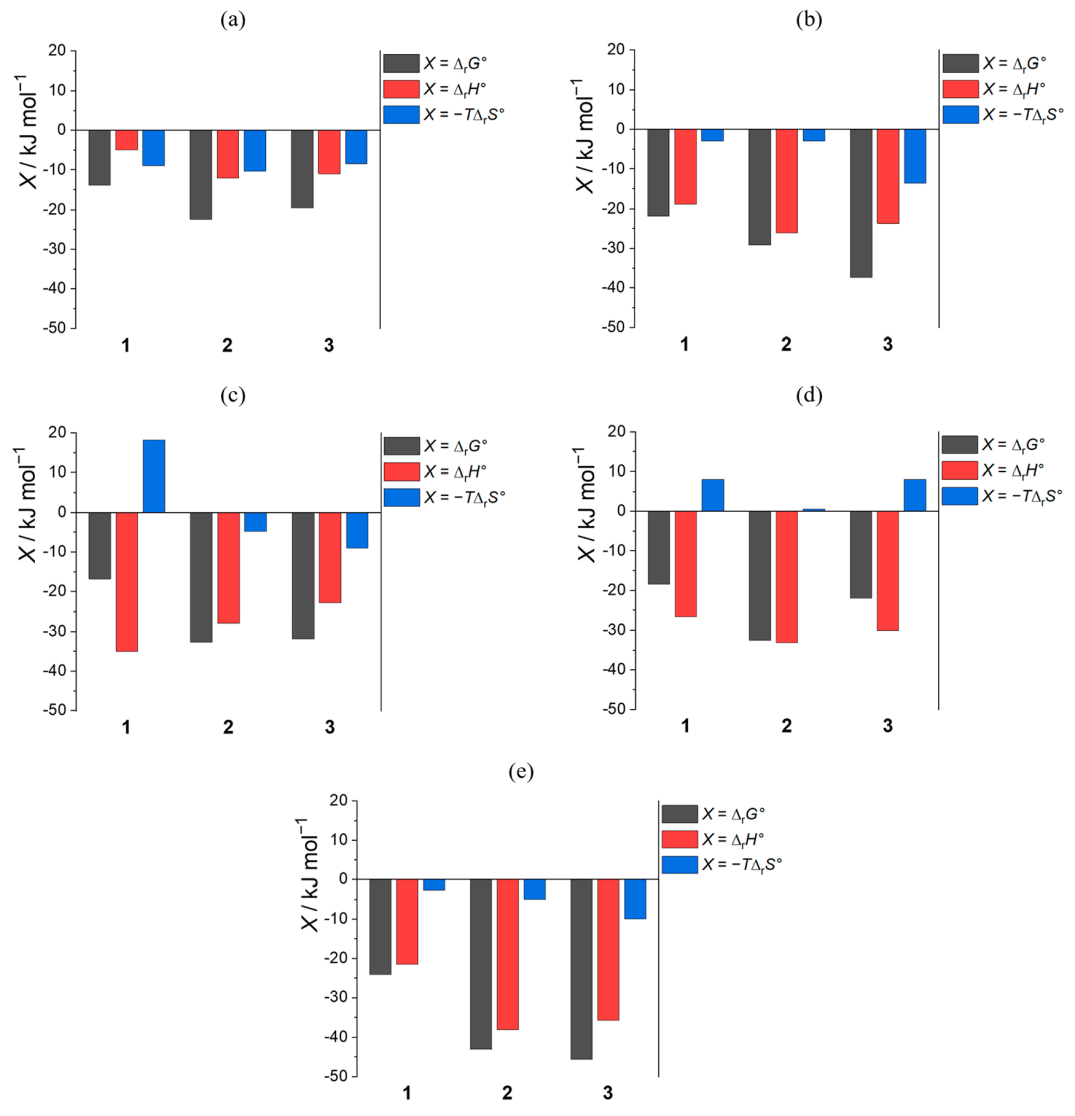
ITC-determined standard thermodynamic parameters for complexation of (a) chloride, (b) dihydrogen phosphate, (c) benzoate, (d) acetate, and (e) fumarate with (thio)urea derivatives of calix[4]arene (**M**A; **M** = **1**, **2**, or **3**) in acetonitrile at *T* = 298 K.

Thermodynamic investigation of binding of anions by (thio)urea derivatives of calix[4]arenes **1**, **2**, and **3** in acetonitrile was generally performed using three methods: ^1^H NMR, UV, and ITC titrations in acetonitrile at 25 °C. In addition, conductometric titration was applied in the case of the **1**HP_2_O_7_^3–^ complex. The anions were used in the form of tetrabutylammonium or tetraethylammonium (in the case of chloride) salts to avoid ion pairing. The stoichiometries of the calixarene (**M**)/anion (A) complexes are given in the **M**_*x*_A_*y*_ form (charges are omitted for clarity) throughout the paper. All complex stability constants determined in this work are listed in Table 2, with the trend of **M**A complex stabilities being mapped graphically in Figure 1. The additional thermodynamic data obtained by ITC are provided in Table 3, again presenting the individual contributions to the reaction Gibbs energy graphically for the sake of clarity (Figure 2). In the continuation of the text, we will discuss the results obtained for each ion and provide an overarching comment in the Conclusions.

**Table 2:** Cumulative Stability Constants of Anion Complexes with Studied Calix[4]arene Derivatives in Acetonitrile at 25 °C^a^

		log β								
	receptor (**M**)	**1**			**2**			**3**		
anion (A)	complex	UV	^1^H NMR	ITC	UV	^1^H NMR	ITC	UV	^1^H NMR	ITC
Cl^–^	**M**A	2.22(3)	2.06(1)	2.47(6)	4.10(2)	4.17(1)	3.92(1)	4.09(1)	3.93(1)	3.47(9)
HSO_4_^–^	**M**A	1.72(2)	1.74(3)	b	3.06(1)	3.12(1)	b	2.66(3)	2.60(1)	2.87(2)
H_2_PO_4_^–^	**M**A	3.90(3)	3.71(4)	3.81^c^	5.1^c^	5.1(2)	5.1^c^	6.54(5)	6.54^c^	6.54^c^
	**M**A_2_	6.73(3)	7.37(2)	7.18(4)	9.23(1)	10.0(1)	9.9(1)	12.32(1)	12.32^c^	12.32^c^
	**M**A_3_							17.31(3)	17.0(1)	17.51(4)
HP_2_O_7_^3–^	**M**A	4.21(1)	4.15(4)	d	5.78(1)	e	5.8^c^	7.90(4)	e	d
	**M**_2_A				10.69(7)	e	11.0(1)	14.02(8)	e	d
BzO^–^	**M**A	3.15(2)	2.85(1)	2.93(2)	5.14(2)	5.18(2)	5.71(4)	6.3^f^	5.59^c^	5.59(4)^g^
	**M**A_2_							11.2^f^	8.66(9)	8.72(2)^g^
									8.73(3)^h^	
AcO^–^	**M**A	3.36(1)^i^	3.31(1)^i^	3.22(5)^i^	5.58(1)^i^	6.16(1)^i^	5.70(5)^i^		4.8^j^	3.8^g^
	**M**A_2_								8.0^j^	7.5^g^
fum^2–^	**M**A	4.30(2)	3.52(1)	4.22(3)	7.5^c^	7.5^c^	7.5(2)	8.01^c^	8.01^c^	8.01(4)^k^
	**M**_2_A				12.49(3)	12.6(1)	12.7(2)	13.73(6)	12.2(1)	11.9(1)^k^
										12.04(4)^l^

a Uncertainties of the last digit are given in parentheses as standard error of the mean (*N* = 3 or 4) or standard deviation in the case of ^1^H NMR titrations.

b Too high dilution enthalpies for reliable quantitative characterization.

c Fixed value (based on the results of other methods) in the fitting process.

d Irreproducible titration results.

e The spectral changes were in line with the binding model developed by other methods but the combination of slow and fast exchange hindered quantitative data processing.

f Values should be considered as assessments due to spectral overlap of the receptors with the benzoate anion.

g Simplified model including only anion binding applied.

h Value obtained from the titration of DBUH**3** with BzOH and then with TBABzO.

i Data published in our previous research.^71^

j Model used for data fitting included the p*K*_a_ value for AcOH (22.23) omitting the acetate homoconjugation.

k Obtained by competitive titration of **1** fum^2–^ with **3**.

l Obtained by direct titration of **3** with fum^2–^.

**Table 3:** Thermodynamic Parameters Related to Reactions *x***M** + *y*A → **M**_*x*_A_*y*_, Where A Stands for Anion and **M** for Calixarene, Determined by ITC in Acetonitrile at 25 °C^a^

	receptor	**1**		**2**		**3**	
A	complex	Δ_r_*H*/kJ mol^–1^	(−*T*·Δ_r_*S*)/kJ mol^–1^	Δ_r_*H*/kJ mol^–1^	(−*T*·Δ_r_*S*)/kJ mol^–1^	Δ_r_*H*/kJ mol^–1^	(−*T*·Δ_r_*S*)/kJ mol^–1^
Cl^–^	**M**A	–5(1)	–8.9(2)	–12.1(1)	–10.3(1)	–11(1)	–8.5(2)
HSO_4_^–^	**M**A	b	b	b	b	–11.1(4)	–5.4(6)
H_2_PO_4_^–^	**M**A	–18.8(4)	–3.0(3)	–26.1(6)	–3.0(6)	–23.7(6)	–13.6(6)
	**M**A_2_	–60(1)	19(2)	–96(5)	40(6)	–58.7(2)	–11.6(2)
	**M**A_3_					–87(2)	–13(2)
HP_2_O_7_^3–^	**M**A	c	c	–16(8)^d^	–18(9)^d^	c	c
	**M**_2_A			–139(3)^d^	76(3)^d^	c	c
BzO^–^	**M**A	–35.0(7)	18.2(9)	–27.9(3)	–4.8(6)	–22.87(6)	–9.0(1)
	**M**A_2_					–69.7(8)	20.0(6)
AcO^–^	**M**A	–26.5(1)^e^	8.1(1)^e^	–33.1(6)^e^	0.6(9)^e^	–30.1^f^	8.1^f^
	**M**A_2_					–71	27.4
fum^2–^	**M**A	–21.5(3)	–2.6(1)	–38(2)	–5(2)	–36.7(5)^g^	–8.9(6)^g^
						–34.8(5)^h^	–10.9(2)^h^
	**M**_2_A			–55(2)	–18(2)	–51.1(4)^g^	–17.6(6)^g^
						–49.4(3)^h^	–18.5(2)^h^

a Uncertainties of the last digit are given in parentheses as standard error of the mean (*N* = 3 or 4).

b Too high dilution enthalpies for reliable quantitative characterization.

c Irreproducible titration curves.

d Somewhat higher uncertainties are due to small heat changes. This experimental issue could not be avoided due to limiting solubility of **2**.

e Data published in our previous research.^71^

f Obtained by a simplified model omitting (de)protonation and acetate homoconjugation from the model.

g Obtained by competitive titration of **1** fum^2–^ with **3**.

h Obtained by direct titration of **3** with fum^2–^.

#### Chloride

The ^1^H NMR titration of **1** with TEACl was accompanied by a downfield shift of urea protons (Figure S10a) which indicated that complexation between **1** and Cl^–^ occurred, stabilized by hydrogen bonds with urea NH acting as the donor. The chemical shifts of other proton signals were also taken into account in the data processing (Figure S10b,c) yielding the value of log β for the 1:1 association reaction (Table 2). The moderate binding of chloride with **1** was confirmed by UV (Figure S11) and ITC (Figure S12) titrations. Both standard reaction entropy and enthalpy were favorable but somewhat modest, resulting in relatively low complex stability (Table 3).

5 to 8 times fewer equivalents of chloride were required in the ^1^H NMR titrations of receptors **2** (Figure S13) and **3** (Figure S16) to obtain the titration curves optimal for quantitative characterization of their complex stabilities. The related stability constants were found to be almost 2 orders of magnitude higher than in the case of **1**Cl^–^ (Table 2). The UV (Figures S14 and S17) and ITC (Figures S15 and 18) results were in agreement with the NMR data. These results highlighted the fact that the number of H-bond-donating moieties played the key role in determining the affinity of studied receptors for chloride. Furthermore, calorimetric measurements revealed that the improvement in stability is due to a change in reaction enthalpy for **2** and **3** vs **1**, whereas the standard reaction entropy remained practically the same for all receptors (Figure 2a). It could be argued that the dominant contribution to the reaction entropy of complexation, in addition to loss of translation entropy, is its increase due to the desolvation of chloride. This effect is almost certainly more pronounced than removal of solvent molecules from the binding sites, since these are relatively confined and the NH groups are involved in intramolecular H bonds. In all three cases, the chloride anion is expected to be (almost) fully desolvated; hence, the entropy change resulting from the binding is not differentiated among the receptors. On the other hand, **2** and **3** can establish a larger number of H bonds with the anion which results in a more significant enthalpy change due to complexation. Interestingly, urea analogue **2** exhibited slightly stronger coordination of chloride compared to **3** (Figure 1), despite the fact that thiourea is generally considered a stronger H-bond donor.

#### Hydrogen Sulfate

The results of NMR (Figure S19) and UV titrations (Figure S20) of receptor **1** with TBAHSO_4_ enabled the characterization of the **1**HSO_4_^–^ complex. Its stability is approximately 3 times lower than that of **1**Cl^–^ (Table 2). Calixarene **2** also forms a complex of 1:1 stoichiometry with HSO_4_^–^ featuring a more than 120 times larger stability constant (Figures S21 and S22) compared to **1**HSO_4_^–^. Unfortunately, highly endothermic dilution of TBAHSO_4_ in MeCN hindered complete thermodynamic insight into the complexation processes for both **1** and **2** by ITC.

The affinity of the tetrathioureido macrocycle **3** for hydrogen sulfate is exactly halfway between the affinities of **2** and **1** for the same anion (Table 2 and Figures S23 and S24). Microcalorimetric titration was successful in the case of **3** (Figure S25) revealing that the formation of **3**HSO_4_^–^ is enthalpically driven with a favorable entropic contribution. Enthalpies of Cl^–^ and HSO_4_^–^ binding are almost the same, while the entropic contribution to the standard Gibbs energy for 3 kJ mol^–1^ is less beneficial for HSO_4_^–^ than in the case of **3**Cl^–^, making **3** a better receptor for chloride (Table 3). Lower stability of all **M**HSO_4_^–^ complexes in comparison to **M**Cl^–^ complexes could be ascribed to the higher basicity of chloride in MeCN [p*K*_bH_(Cl^–^) = 10.3 vs p*K*_bH_(HSO_4_^–^) = 7.6].^69^

#### Dihydrogen Phosphate

Before discussing the results regarding complexation of H_2_PO_4_^–^, a peculiar property of this anion, i.e., its ability to dimerize, should be recalled. This phenomenon was fully thermodynamically characterized by us [log *K*((H_2_PO_4_)_2_^2–^) = 3.38, Δ_r_*H*° = −27.5 kJ mol^–1^].^77^ In a typical titration experiment, when TBAH_2_PO_4_ titrant solution is added to a solution of the receptor, dissociation of dihydrogen phosphate dimers takes place, and this must be accounted for. The incorporation of the related effects in the fitting procedure of the ITC titration data was devised by Horvat et al.^78^ and was applied in this work.

After the above-described pretreatment of the data, the shape of the microcalorimetric titration curve (Figure S28b) related to the system including **1** and TBAH_2_PO_4_ clearly indicated a formation of two types of complexes, most likely **1**H_2_PO_4_^–^ and **1**(H_2_PO_4_^–^)_2_. Unfortunately, the convergence of the fit could not be achieved when all parameters, i.e., the related stability constants and reaction enthalpies, were unknown. On the other hand, ^1^H NMR titration data (Figure S26) could be fitted, confirming that two quite stable complexes (**1**H_2_PO_4_^–^ and **1**(H_2_PO_4_^–^)_2_) occurred in the solution and affording the related stability constants. With the equilibrium constant of the 1:1 complex formation at hand, we were able to fix this value in the process of ITC data fitting. This resulted in convergence of the fit and complete thermodynamic characterization of the equilibria. Thus, the multiple-method strategy in exploring the anion-binding processes was particularly rewarding for this system. Both methods indicated that the binding of the first anion was thermodynamically a bit more favorable compared to the association of the second anion (Table 2). The stability of both types of complexes was ensured by favorable reaction enthalpies (Table 3), despite the substantial unfavorable entropic term in the case of binding the second anion. Further on, the UV titration results (Figure S27) agreed with the model, although providing somewhat different complex stability constants, which can be attributed to reduced reliability due to rather similar characteristic spectra of the two complexes formed.

NMR titration of receptor **2** with TBAH_2_PO_4_ clearly demonstrated the existence of multiple complexes (**M**A and **M**A_2_) in the solution, as evidenced by the changes in the trend of the titration curve (Figure S29). A decrease in the chemical shift at low *n*(H_2_PO_4_^–^)/*n*(**2**) followed by an increase as excess of anion was added afforded unambiguous identification of the underlying processes, i.e., complex stoichiometries. The stability of the **2**(H_2_PO_4_^–^)_2_ was additionally determined by UV and ITC titrations (Figures S30 and S31), whereby the log *K*(**M**A) had to be fixed at the value obtained by NMR, again pointing out the value of the multimethod approach. The tetraureido analogue outperformed the host containing only two binding moieties, albeit the difference in the stability of the **M**A complex was lower than in the case of chloride and hydrogen sulfate (Table 2).

The results regarding H_2_PO_4_^–^ binding by thioureido-calixarene **3** are especially interesting. Namely, the results of the corresponding UV titration (Figure S32) could be explained only by including three complexes in the fitting model: **M**A, **M**A_2_, and **M**A_3_ (Table 2). High stability constants were determined for all three complexes. Although we do not have specific insight into the structural features of the resulting complexes, it is plausible that two thiourea moieties coordinate the dihydrogen phosphate dimer (two anions interconnected by a hydrogen bond), while the third anion is bound to the two remaining binding sites. The existence of three different complexes of H_2_PO_4_^–^ with **3** in MeCN has also been corroborated by NMR and ITC titrations (Figures S33 and S34). It is worth noting that the same complex stoichiometry has also been discovered within our research group recently, using hexalysine cyclopeptide (**P**) with Boc-protected side chains,^79^ pointing out that H_2_PO_4_^–^ is prone to formation of these complexes if provided with the right binding site. An interesting observation regarding the entropy contribution to the formation of complexes of higher stoichiometries for the investigated calixarenes with H_2_PO_4_^–^ is that in cases of urea receptors, this contribution is (very) unfavorable, whereas for the thiourea receptor, the entropy term is favorable. This can be, at least partially, explained by the (experimentally confirmed) better solubility of receptor **3**, i.e., greater entropic disruption of MeCN around **3** caused by its association with H_2_PO_4_^–^.

**Figure 3:**
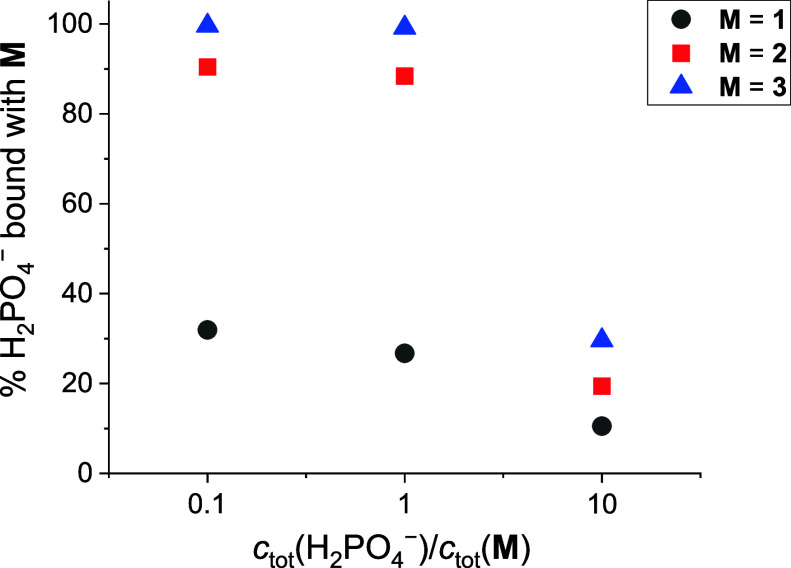
Comparison of the dihydrogen phosphate complexation percentage in acetonitrile solutions containing receptors (**M**) at 25 °C. *c*_tot_(**M**) = 7.5 × 10^–5^ mol dm^–3^ (which corresponds to the solubility of **2**).

The binding power of the investigated calixarenes toward dihydrogen phosphate is also depicted via the percentage of the bound anion (Figure 3), demonstrating practically the effect of variation in stability constants. The supremacy of **3** in the context of interactions with H_2_PO_4_^–^ is obvious, especially when the greater solubility of this receptor (vs ureido-calixarenes **2** and **1**) is considered.

#### Hydrogen Pyrophosphate

The UV titration of receptor **1** with TBA_3_HP_2_O_7_ was accompanied by a hyperchromic shift which could be accounted for by assuming the formation of the 1:1 complex (Figure S35). The stability of the **1**HP_2_O_7_^3–^ complex was 2 times greater than that of the analogous complex with H_2_PO_4_^–^ (Table 2), which can be attributed to multiple negatively charged oxygen atoms at hydrogen pyrophosphate available for the interaction with urea moieties. NMR titration (Figure S36) confirmed the conclusions drawn from the UV result, but the ITC titrations were somewhat irreproducible (Figure S37), which disabled further insight into the thermodynamics of complexation for this system. The high charge of the tested anion, in combination with a significant difference in size between **1** and HP_2_O_7_^3–^, motivated us to perform a conductometric titration of the TBA_3_HP_2_O_7_ solution with the receptor. Indeed, a significant decrease in molar conductivity of salt solution was observed upon the addition of **1** (Figure S38), which agreed with the assumption of the complex being less mobile than the free anion. A high-quality fit of the conductometric titration curve was obtained by applying the 1:1 binding model, yielding the association constant which was in excellent agreement with the values acquired by NMR and UV (log β = 4.09(2)).

Further on, it was found that the tetraureido receptor **2** bound hydrogen pyrophosphate more efficiently than **1** (Table 2) forming **M**A and an additional complex of interesting stoichiometry, namely, **M**_2_A. This was detected by UV, ITC, and NMR experiments (Figures S39–S41), whereby the former two enabled quantitative characterization of the complexes. The formation of **M**_2_A can be rationalized by considering the fact that hydrogen pyrophosphate features two distinct H-bond acceptor sites and is large enough for two hosts to approach it without significant steric hindrances. Consequently, hydrogen pyrophosphate acts as a supramolecular linker between two calix[4]arene units.

The highest affinity toward HP_2_O_7_^3–^ was obtained in the case of tetrathioureido-calix **3** (Table 2) which exhibited analogous behavior in terms of complex stoichiometry. Although quantitative results for the stability of the corresponding complexes emerged only from UV experiments (Figure S42), the application of these values for modeling the NMR titration data provided a reasonable agreement of the experimental and calculated data (Figure S43). Namely, fast exchange was attributed to the equilibrium between the **M** and **M**_2_A complexes, whereas slow exchange characterized the equilibrium between **M**_2_A and **M**A species.

Examples of thermodynamic characterization of the complexation of hydrogen pyrophosphate with neutral macrocyclic receptors in acetonitrile are rare. However, Danil de Namor et al. reported ITC results for HP_2_O_7_^3–^ binding by several calix[4]pyrrole derivatives and a hydroxyamide derivative of calix[4]arene (Figure S46; Table S17).^80−82^ For all four receptors, both **M**_2_A and **M**A types of associates were found. Furthermore, a highly unfavorable entropy term overcompensated with a very favorable enthalpy term characterized the formation of **M**_2_A which is in line with the above-described results for **2** and **3**.

#### Fumarate

The titration curves for the system involving **1** and fumarate obtained by NMR (Figure S49), UV (Figure S50), and ITC (Figure S51) all suggested the formation of a very stable complex of 1:1 stoichiometry (Table 2). This complexation process was enthalpically driven, with the reaction enthalpy less favorable than for other explored carboxylic anions. A specific feature of fumarate complexation was the favorable entropic contribution, which was significantly unfavorable for other carboxylate complexes (Figure 2). This can again be attributed to variations in solvation thermodynamics related to both the anion and complex.

**Figure 4:**
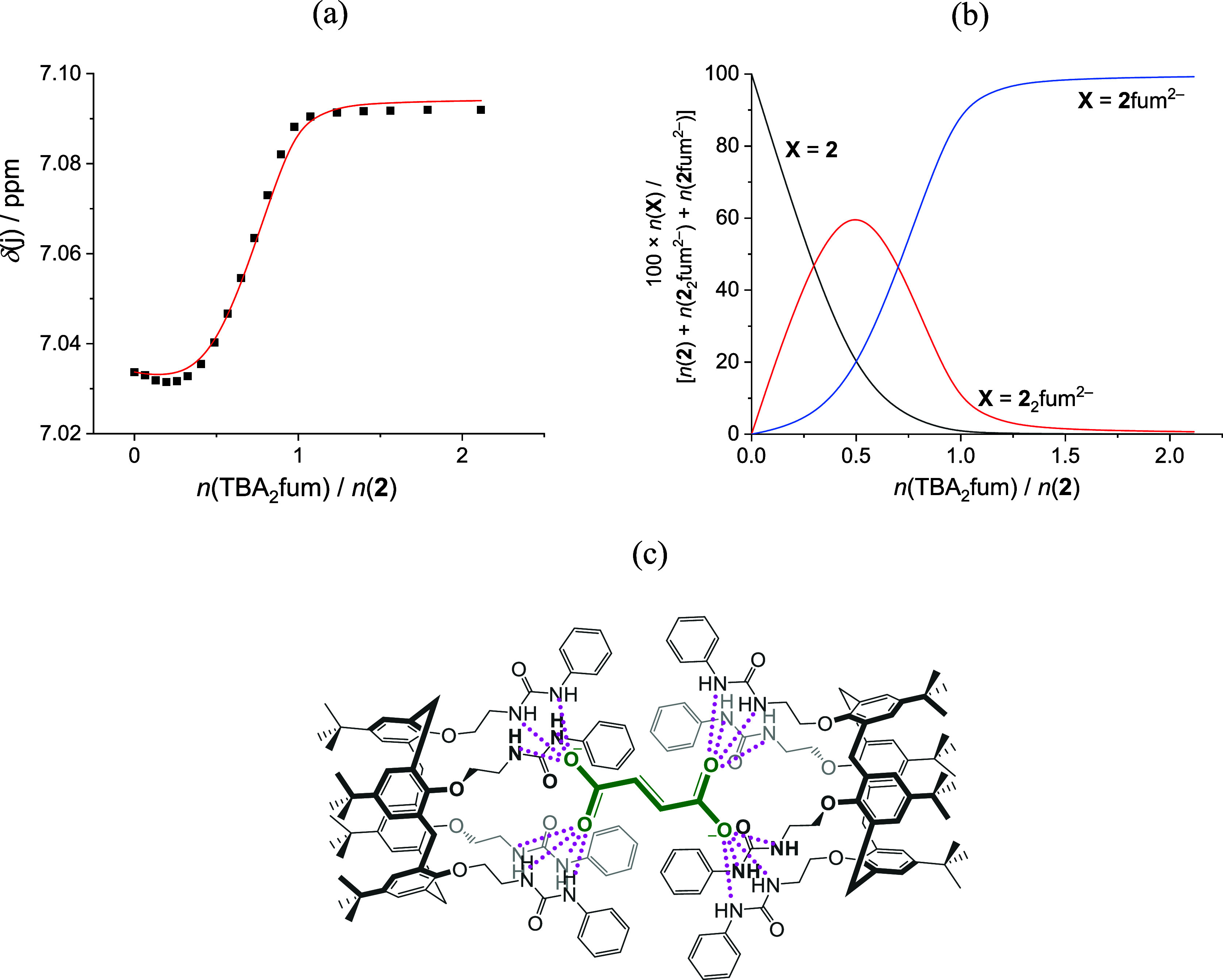
^1^H NMR spectroscopy titration of **2** (*c* = 7.28 × 10^–5^ mol dm^–3^, *V*_0_ = 500 μL) with TBA_2_fum (*c* = 2.37 × 10^–4^ mol dm^–3^) in CD_3_CN at 25 °C. Spectra available in Figure S54. (a) Experimental [■ (black)] and calculated [— (red)] chemical shifts for the selected nucleus at **2**. (b) Distribution of **2** and its complexes with fumarate. (c) Proposed structure of complex **2**_2_fum^2–^. Hydrogen bonds are given in magenta.

Similar to the results regarding hydrogen pyrophosphate, in the cases of **2** and **3**, the two carboxylate groups of fumarate encouraged the formation of a complex comprising two calixarene molecules bridged by the dianion (Figures S52 and S55). The binding of the second receptor was characterized by a lower successive binding constant compared to the simple **M**A complex. Still **M**_2_A was a dominant species at the appropriate receptor-to-anion molar ratio (2:1). The presence of two types of complexes between tetrasubstituted hosts and fumarate was confirmed by UV (Figures S53 and S58) and NMR titrations (Figures 4 and S57). The approach applying multiple methods for determining complexation equilibrium constants proved to be of particular value in this case. Namely, the log β(**M**A) obtained by ITC had to be incorporated (fixed) in the models used for fitting UV and NMR titration data, resulting in excellent agreement of the remaining stability constant and alignment of the calculated data with the experimental ones.

The stability of the complexes comprising **3** and fumarate is very high and lies at the upper limit for reliable determination by direct titration experiments. Therefore, to validate these results, competitive microcalorimetric titration of the fumarate complex of **1** with **3** was performed (Figure S56). All thermodynamic parameters determined in this way matched those calculated from the direct ITC titration. The stability of both **M**_2_A and **M**A complexes was ensured by favorable enthalpic and entropic contributions. Given the particular stability of the fumarate complexes, these systems could be considered as a basis for the development of more elaborate supramolecular systems such as polymers or pseudomacrocycles with the fumarate anion acting as the link between (thio)urea-containing fragments.

#### Acetate

Complexation of acetate by urea derivatives of *p*-*tert*-butylcalix[4]arene in MeCN was elaborated in our previous paper.^71^ By performing the receptor acidity measurements in this work (Table 1), we confirmed the validity of the previously reported data. Namely, the p*K*_a_ values for both hosts are high enough to maintain acetate in its deprotonated state (Δp*K*_a_(**1** vs AcOH) = 9.6, Δp*K*_a_(**2** vs AcOH) = 2.5) in acetonitrile solution. Therefore, the incorporation of proton transfer into the fitting model for the results of the corresponding titrations with acetate was indeed not required. On the other hand, higher acidity of **3** suggested that the experiments regarding solutions of acetate and **3** should be treated with more scrutiny. At first, all known proton dissociation processes were taken into account in the course of the NMR (Table S21 and Figure S60) and UV (Figure S61) data processing.^83^ A reasonably good fit of the NMR data was obtained only when the homoconjugation of acetate was ignored (Figure S60). Thus, obtained complex stability constants resembled those determined by processing the ITC titration curve using a simplified model (Tables 2 and 3, and Figure S62). However, the results provided by UV were in strong disagreement with the above-described data, regardless of the model used, and suggested much more tight binding of the anion. We did not find the stability constants obtained by UV titrations realistic and thus did not list them. Still, the fact that the data was not consistent among the methods remained unresolved. Undoubtedly, we have shown that the thiourea calixarene does interact with acetate in MeCN, and we have assessed the stabilities of the related complexes, but consistent thermodynamic description with the desired level of reliability was not achieved in this case.

#### Benzoate

The affinity of bisureido receptor **1** for BzO^–^ was found to be almost 10 times lower than for H_2_PO_4_^–^ (Table 2 and Figures S63–S65) in spite of the pronounced basicity of benzoate (p*K*_a_ = 21.5).^69^ The cause of this relation was substantially unfavorable entropic contribution to the formation of **1**BzO^–^ which lowered the very favorable complexation enthalpy (Table 2). On the other hand, calixarene with four urea moieties (**2**) bound benzoate with almost the same affinity as H_2_PO_4_^–^ (when comparing **M**A complexes). Namely, the multivariate fit of the NMR titration of **2** with TBABzO (Figure S66) enabled the calculation of the association constant for **2**BzO^–^: log β = 5.18 (Table 2). This value was in excellent agreement with the corresponding value obtained by UV titration (Figure S7). Microcalorimetry revealed that the complexation of BzO^–^ with **2** was mainly enthalpically driven, but the entropic term was also favorable, unlike in the case of **1** (Figure S68). As such a difference between complexation entropies for **1** and **2** with Cl^–^ and H_2_PO_4_^–^ was not observed (Figure 2), the above observation implies better solvation of **1**BzO^–^ relative to **2**BzO^–^.

Before the investigation of interactions between benzoate and the most acidic of the herein used receptors, i.e., **3**, the homoconjugation of benzoate in MeCN was tested. To be clearer, the p*K*_a_ difference between **3** and benzoic acid is 1.2, with **3** being the weaker acid. Arguably, this value did not imply severe proton transfer from the receptor to anion. However, we suspected that another process, potentially playing an important role in MeCN solutions containing benzoate and benzoic acid, has been overlooked in prior research. Namely, we found that in aprotic solvents, homoconjugation is often a rather favorable process,^83^ and the formation of BzOHBzO^–^ could be an important factor defining the speciation in the solutions of **3** and BzO^–^. To investigate the homoconjugation reaction, we performed ^1^H NMR titration of benzoic acid with TBABzO (Figure S69) which enabled the calculation of the stability constant for BzOHBzO^–^ (log *K* = 3.30(3)). Formation of BzOHBzO^–^ species was also detected by ITC, although this method provided a somewhat higher value of the homoconjugation constant (log *K* = 3.80(2), Δ_r_*H*° = −27.3(4) kJ mol^–1^, Figure S70). By accessing these parameters, we were able to treat the experimental data regarding the reactions between **3** and BzO^–^ with much more scrutiny as described below.

The binding of BzO^–^ by **3** was first investigated via ITC titrations (Figure S71). The successive exothermic heat signals could be fitted by assuming the formation of complexes **M**A and **M**A_2_ (Table 2, model A in Table S26) exclusively. The formation of both complexes was enthalpically driven with the entropy contribution being favorable in the case of **3**BzO^–^ and unfavorable for the **M**A_2_ complex (Table 3). Following the chemical shifts of protons of **3** in the NMR titration with TBABzO confirmed the presence of two types of complexes between **3** and BzO^–^ (Figure S72) detected also by ITC. This was especially clear by inspecting the aryl proton signals which featured an intricate trend, i.e., initial decrease followed by an increase in the chemical shift due to the relation δ(**M**A) < δ(**M**) < δ(**M**A_2_) (Table S27). The fitting of the NMR titration data using model A required fixing the stability constant related to **M**A, which was done by using the result gained from ITC. The resulting stability constant for **M**A_2_ matched very well to the ITC value, and the quality of the fit was again satisfactory. In order to evaluate the impact of the possible protonation and homoconjugation of the anion on the speciation of the solution containing **3** and BzO^–^, a global model B (Table S26) was applied to the NMR data (Figure S73). It is evident from the corresponding speciation diagram (Figure S73b) that proton transfer is practically negligible (≤2%) despite the relatively favorable BzO^–^ homoconjugation. Consequently, these processes had almost no effect on the titration curves within the used BzO^–^ concentration range of. The same conclusion could be derived from the comparison of β(**M**A_2_) values obtained by the two methods, which were virtually the same. UV titration of **3** with BzO^–^ was performed (Figure S74) and although the data could be fitted according to the simplified procedure (model A in Table S23), this yielded much higher stability constants compared to the ones obtained by other methods, most likely due to the significant spectral overlap of titrand and titrant solutions. Therefore, we deemed the values unreliable and did not report them.

**Figure 5:**
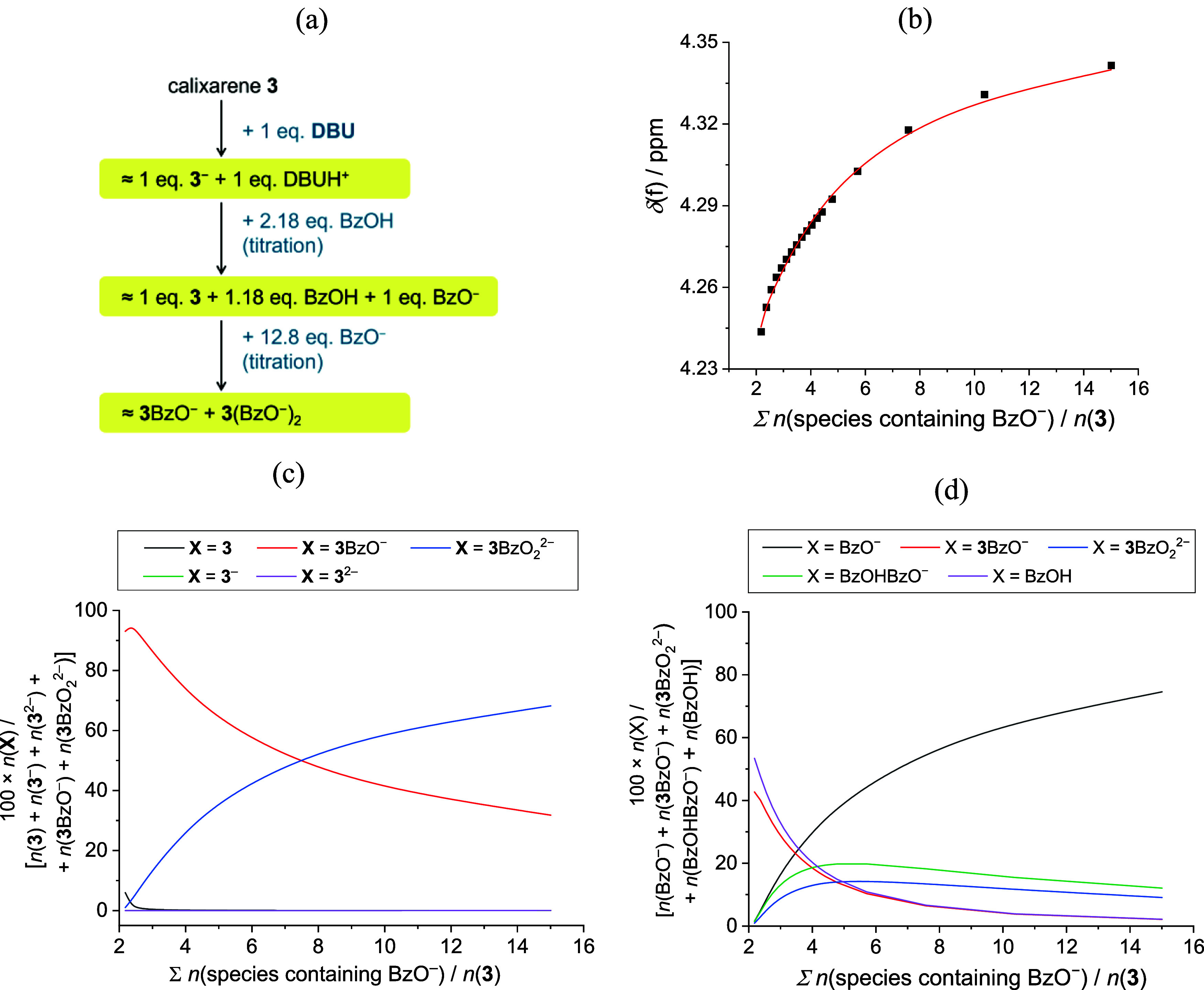
(a) ^1^H NMR titration of **3**^–^ (*c* = 5.96 × 10^–4^ mol dm^–3^, *V*_0_ = 450 μL, deprotonation executed with the addition of 1 mol equivalent DBU base in 35 μL) first with BzOH (*c* = 3.25 × 10^–2^ mol dm^–3^) and then with TBABzO (*c* = 9.96 × 10^–3^ mol dm^–3^) in CD_3_CN at 25 °C. NMR spectra available in Figure S71. (b) Experimental [■ (black)] and calculated [**—** (red); based on full model X, see Table S26] chemical shifts for the selected nucleus at **3**. Distribution of (c) calixarene **3** and (d) benzoate between all possible species containing them during the titration.

After characterizing the system in detail, we carried out an additional NMR experiment, which pointed out more profoundly the virtue of a thorough portrayal of an array of equilibria established in the solution. To a solution of **3** deprotonated with 1 equivalent of DBU base, we added 2.2 equivalent of BzOH (Figure 5). According to the equilibrium constants discussed above, this should result in quantitative reprotonation of the receptor and cause more than 90% of the calixarene to be in the form of an anion complex, with the 1:1 stoichiometry being the dominant one. The spectral changes measured during such experiment directly matched the described expectations.^a^ Further addition of benzoate salt was expected to push the equilibrium toward **M**A_2_. The spectral changes detected during this stage were fully in line with the described picture of interconnected equilibria (Figures 5 and S75). In order to quantitatively process these data, including the homoconjugation reaction was necessary, since the percentage of the homoconjugate was significant in this experimental setup. This yielded the stability constant for **M**A_2_, closely matching that obtained by previously discussed ITC and NMR experiments (Table 2), clearly demonstrating that the importance of side processes strongly depended on the conditions applied. Namely, the observations during this experiment could not be rationalized without knowing the acid–base properties and other supramolecular behaviors of all species involved. In other words, although the proton transfer did not play a significant role in the experimental conditions applied during the titrations of **3** with benzoate salt, it is necessary to take into account the related equilibria too when dealing with systems containing various bases or acids.

## Conclusions

In this work, we combined two essential motifs of supramolecular chemistry: calixarene scaffold and (thio)urea moiety as the binding site developing a series of anion receptors. The thermodynamics of anion binding of the resulting molecules in acetonitrile was comprehensively mapped, providing a detailed insight into their structure–reactivity relationship.

Within a series of tested anions, only Cl^–^ and HSO_4_^–^ formed simple 1:1 complexes with the studied receptors, whereas others gave rise to a variety of complex stoichiometries. Specifically, the tetraurea derivative was capable of binding up to three dihydrogen phosphate anions. Such a complex is rarely encountered, and its existence showcases the extent of favorable interactions between the bound dihydrogen phosphate anions. Further on, the fumarate and hydrogen pyrophosphate could bridge two host molecules, resulting in 2:1 (receptor/anion) complexes with both prepared tetrasubstituted receptors. This finding was in line with the structural features of the anions, since they contain two distinct acceptor sites. Their stability suggested that analogous interactions could be used for the construction of larger supramolecular oligomers (and polymers) bridged by divalent anions. When comparing the performance of all three studied hosts, the one containing only two NH groups binds anions significantly less strongly compared to tetrasubstituted ones. On the other hand, the stability of complexes with urea and thiourea derivatives was rather similar in most cases despite the greater acidity of the thioureas. Exceptions were dihydrogen phosphate and pyrophosphate, which we found to prefer thioureas as the binding site. Standard reaction entropy was positive for most of the studied cases, but generally, the main driving force for complex formation was the exothermicity of the studied processes.

It should be pointed out that the characterization of several complexation reactions taking place simultaneously in a solution requires significant effort, and often application of several methods is necessary to obtain reliable data. This was indeed the case throughout our study, so we performed NMR, ITC, UV, and conductometric experiments, analyzing the related data in a synergistic manner. This means that the information provided by one method was included in the processing of data obtained by others, leading to a consistent model. Having mentioned the complexity of equilibrium systems, an important point of this work is that one should be aware of the possible proton transfer from receptors containing NH groups to basic anions. All related experimental and fitting procedures are described in detail, adding a methodical value to the reported research.

To conclude, by thorough analysis of anion binding by relatively simple calix[4]arene-based hosts bearing (thio)urea binding sites, we discovered a variety of complex stoichiometries, confirming their existence by several methods. The thermodynamic driving force affording their stability was determined by calorimetric measurement, and their formation rationalized by considering the structural features of both anions and the receptors. Thus, we provided a comprehensive description of fundamental supramolecular interactions, which could be exploited in designing numerous elaborate systems in the future.

## Experimental Section

### Synthesis

The synthetic procedures for urea derivatives of calixarene, **1** and **2**, are available in our recent publication,^71^ whereas the synthesis and characterization of thioureido-calixarene **3** is given in the Supporting Information.

Preparation of tetrabutylammonium fumarate was done according to a slightly modified procedure known from the literature.^84^ Namely, to a solution of fumaric acid (173.0 mg, 1.49 mmol; S. Aldrich, ≥99%) in methanol (15 mL), TBAOH (2.98 mmol; 2.120 mL of 40 wt % TBAOH in MeOH solution produced by Acros Organics)* was added. Commercial TBAOH solution in methanol was standardized before use in the synthesis (Figure S47). The resulting mixture was sonicated for 5 min, i.e., until complete dissolution. The MeOH was then evaporated under reduced pressure, and the resulting salt was dried over P_2_O_5_ for 24 h before use (drying with an oil–vacuum pump was also tried and had the same effect). The assay of TBA_2_fum in the dried whitish oily product was 79.98%. The identity of the product was confirmed by ^1^H NMR (Figure S48).

### Physicochemical Measurements

The solvents, acetonitrile (MeCN; J. T. Baker, HPLC grade, ≤0.05% water, and Fluka, HPLC grade, 0.01% water), and deuterated acetonitrile (Eurisotop, +0.03% TMS, 99.80% D, <0.05% water) were used without further purification. For the characterization of the acidity of calixarenes, two bases were used: 1,8-diazabicyclo[5.4.0]undec-7-ene (DBU; S. Aldrich, 98%) and 1-ethyl-2,2,4,4,4-pentakis(dimethylamino)-2λ^5^,4λ^5^-catenadi(phosphazene) (P_2_Et; S. Aldrich, >98.0%). Due to the inertness of tetraalkylammonium cations regarding ion association, salts used for anion-binding investigations contained these bulky cations. Namely, the following salts were used: TEACl (S. Aldrich, ≥98.0%), TBAHSO_4_ (S. Aldrich, ≥99.0%), TBAH_2_PO_4_ (S. Aldrich, ≥99.0%), TBA_3_HP_2_O_7_ (S. Aldrich, ≥97.0%), TBABzO (S. Aldrich, ≥99.0%), TBAOAc (Merck, 97%), and TBA_2_fumarate (prepared in-house).

Notation for titration experiments described below: titrand = **1**, **2**, and **3**; titrant = TEACl, TBAHSO_4_, TBAH_2_PO_4_, TBA_3_HP_2_O_7_, TBABzO, TBAOAc, and TBA_2_fumarate.

In all cases, the results of the titrations were graphically presented by using OriginPro 2021.

### ^1^H NMR

^1^H NMR spectra were recorded by means of Bruker AVANCE III HD 400 MHz/54 mm and Bruker AVANCE Neo 600 MHz/54 mm NMR spectrometers, equipped with an inverse broadband room-temperature probe (5 mm PA BBI 1H/D-BB) and inverse triple-resonance TCl Prodigy cryoprobe (5 mm CPP1.1 TCl 600S3 H&F-CIN-D-05 XT), respectively. All proton spectra were acquired at 25.0 °C by using 64 K data points, a spectral width of 20 ppm, a recycle delay of 1.0 s, and 16, 32, or 64 scans. CD_3_CN was used as a solvent, and TMS was used as an internal standard for proton chemical shifts. ^1^H NMR titrations were performed by recording the spectral changes in titrand solutions (*c*_0_ = 0.5 to 0.9 mmol dm^–3^ for **1** and **3**; *c*_0_ = 0.05 to 0.09 mmol dm^–3^ for **2**, *V*_0_ ≈ 0.5 mL) upon stepwise addition of titrant solutions (*c* = 1 mmol dm^–3^ to 0.4 mol dm^–3^, depending on the system). Experimental conditions for (de)protonation and homoconjugation experiments are provided in Figures S6–S8, S66, and S72. The dependences of selected proton chemical shifts on the concentrations of reactants were processed using the HYPNMR2008 program,^73^ whereas for the presentation of the results, MestReNova was used along with OriginPro 2021.

### Microcalorimetry

Microcalorimetric measurements were performed by isothermal titration calorimeters MicroCal VP-ITC (*V*_cell_ = 1.43 to 1.45 mL) at 25.0 °C. The enthalpy changes were recorded upon stepwise, automatic addition of titrant solution (*c* = 0.1 to 0.2 mmol dm^–3^ for **1**, *c* = 0.2 to 0.8 mmol dm^–3^ for **3**, and *c* = 0.07 mmol dm^–3^ for **2**) to titrand solution (*c* = 0.8 mmol dm^–3^ to 0.03 mol dm^–3^; depending on the system). The conditions for one competitive ITC titration and homoconjugation titration are described in Figures S56 and S67. Blank experiments were carried out in order to make corrections for the enthalpy changes corresponding to dilution of the titrant solution in the pure solvent. The dependence of successive enthalpy change on the titrant volume was processed using Microcal OriginPro 7.0 in the case of direct titration, whereas data obtained by competitive titration, as well as data from titrations where complexes of stoichiometry higher than 1:1 were detected, were processed using the HypDH program.^85^ Titrations for each system were repeated at least three times.

### UV Spectrophotometry

Spectrophotometric titrations were carried out at (25.0 ± 0.1) °C by means of Agilent Cary 5000 and Agilent Cary 60 spectrophotometers equipped with thermostatting devices. The spectral changes of titrand solutions (*c*_0_ = 0.1 to 0.2 mmol dm^–3^ for **1**, *c*_0_ = 0.05 to 0.1 mmol dm^–3^ for **2**, and *c*_0_ ≈ 0.02 mmol dm^–3^ for **3**; *V*_0_ = 2.3 mL) were recorded upon stepwise addition of titrant solutions (*c* = 2 × 10^–4^ to 2 × 10^–2^ mol dm^–3^; depending on the system) into the measuring quartz cell (Hellma, Suprasil QX, *l* = 1 cm). Absorbances were sampled at 1 nm intervals with an integration time of 0.2 s. The obtained spectrophotometric data were processed using the HypSpec program.^85^

### Conductometry

The conductometry titration (details in Figure S38) was performed using a MettlerToledo InLab 741-ISM conductivity cell (*K*_cell_ = 0.09806 cm^–1^) calibrated with a standard KCl solution (Merck, κ = 84.00 mS cm^–1^) connected to a MettlerToledo SevenExcellence measuring device. Conductivity data were collected automatically via the MettlerToledo EasyDirect program every 10 s, whereas the time gap between two consecutive titrant additions was 5 min. The temperature of the sample was kept constant at 25.0 °C using a JULABO thermostat.

### Single-Crystal X-ray Diffraction Experiments

Crystals of **3**C_2_H_5_OH were obtained by crystallization from ethanol. The X-ray diffraction data were collected via ω scans on a Rigaku Synergy diffractometer equipped with a 4-circle kappa geometry goniometer, HyPIX-6000 detector, and microfocus Cu Kα source (λ = 1.54184 Å) at 298(2) K. The data processing was performed using the CrysAlisPro software package.^86^ The structure was solved with dual space methods using SHELXT.^87^ The refinement procedure by full-matrix least-squares methods based on *F*^2^ values against all reflections included anisotropic displacement parameters for all non-H atoms. The most disordered *t*-butoxy group was modeled with two fragments of occupancy 0.5. Hydrogen atoms attached to carbon, nitrogen, and oxygen atoms were placed in geometrically idealized positions and were refined using the riding model with *U*_iso_ = 1.2*U*_equiv_ of the connected carbon atom or as ideal CH_3_ groups with *U*_iso_ = 1.5*U*_equiv_. Determination of hydrogen atom positions from the difference map was unsuccessful. All refinements were performed using SHELXL.^88^ The SHELX programs operated within the Olex2 suite.^89^

CCDC 2372841 contains the supplementary crystallographic data for this paper. These data can be obtained free of charge via http://www.ccdc.cam.ac.uk/conts/retrieving.html (or from the Cambridge Crystallographic Data Centre, 12, Union Road, Cambridge CB2 1EZ, UK; fax: +44 1223 336033).

## Supporting Information Available

Calixarene **3**—synthesis and characterization; proof of absence of self-association of **3** in MeCN; assignment of proton NMR signals of receptors **1** and **2**; acidity of receptors; dealing with high values of protonation constants in HYPNMR; anion-binding investigation (chloride, hydrogen sulfate, dihydrogen phosphate, hydrogen pyrophosphate, and fumarate); and test of binding of several acids with **3** (acetate and benzoate) (PDF)

## Footnotes

a To make sure there is no interaction of **3** with the protonated form of the anions, we performed the titrations of **3** with benzoic, acetic, and phosphoric acid and expectedly saw no evidence of any interactions.

## References

[ref1] Chen L.; Berry S. N.; Wu X.; Howe E. N. W.; Gale P. A. Advances in Anion Receptor Chemistry. Chem 2020, 6 (1), 61–141. 10.1016/j.chempr.2019.12.002.

[ref2] Macreadie L. K.; Gilchrist A. M.; McNaughton D. A.; Ryder W. G.; Fares M.; Gale P. A. Progress in Anion Receptor Chemistry. Chem 2022, 8 (1), 46–118. 10.1016/j.chempr.2021.10.029.

[ref3] Jolliffe K. K. A.; Gale P. A. The Supramolecular Chemistry of Anions. Org. Biomol. Chem. 2022, 20 (4), 713–714. 10.1039/D1OB90183D. 35024711

[ref4] de Jong J.; Bos J. E.; Wezenberg S. J. Stimulus-Controlled Anion Binding and Transport by Synthetic Receptors. Chem. Rev. 2023, 123 (13), 8530–8574. 10.1021/acs.chemrev.3c00039. 37342028 PMC10347431

[ref5] Gloe K.; Stephan H.; Grotjahn M. Where Is the Anion Extraction Going?. Chem. Eng. Technol. 2003, 26 (11), 1107–1117. 10.1002/ceat.200306105.

[ref6] Zhang Q.; Zhou Y.; Ahmed M.; Khashab N. M.; Han W.; Wang H.; Page Z. A.; Sessler J. L. Anion Extractants Constructed by Macrocycle-Based Anion Recognition. J. Mater. Chem. A 2022, 10 (29), 15297–15308. 10.1039/D2TA03791B.

[ref7] Trotta A.; Jacobsen E. N. Chiral Ureas, Thioureas, and Squaramides in Anion-Binding Catalysis with Co-Catalytic Brønsted/Lewis Acids. Anion-Binding Catalysis; John Wiley & Sons, Ltd, 2022; pp 141–159.

[ref8] Chwastek M.; Cmoch P.; Szumna A. Anion-Based Self-Assembly of Resorcin[4]Arenes and Pyrogallol[4]Arenes. J. Am. Chem. Soc. 2022, 144 (12), 5350–5358. 10.1021/jacs.1c11793. 35274940 PMC8972256

[ref9] Danil de Namor A. F. D.; Hamdan W. A.; Webb O.; Bance-Soualhi R.; Howlin B.; Al Hakawati N. Calix[4]Arene Urea Derivatives: The Pathway from Fundamental Studies to the Selective Removal of Fluorides and Phosphates from Water. J. Hazard. Mater. 2019, 364, 733–741. 10.1016/j.jhazmat.2018.07.025. 30419542

[ref10] Tzioumis N. A.; Cullen D. A.; Jolliffe K. A.; White N. G. Selective Removal of Sulfate from Water by Precipitation with a Rigid Bis-Amidinium Compound. Angew. Chem., Int. Ed. 2023, 62 (12), e202218360 10.1002/anie.202218360. 36702770

[ref11] Jackson D. T.; Nelson P. N. Preparation and Properties of Some Ion Selective Membranes: A Review. J. Mol. Struct. 2019, 1182, 241–259. 10.1016/j.molstruc.2019.01.050.

[ref12] Butler S. M.; Jolliffe K. A. Molecular Recognition and Sensing of Dicarboxylates and Dicarboxylic Acids. Org. Biomol. Chem. 2020, 18, 8236–8254. 10.1039/D0OB01761B. 33001119

[ref13] Busschaert N.; Caltagirone C.; Van Rossom W.; Gale P. A. Applications of Supramolecular Anion Recognition. Chem. Rev. 2015, 115 (15), 8038–8155. 10.1021/acs.chemrev.5b00099. 25996028

[ref14] Amendola V.; Fabbrizzi L.; Mosca L. Anion Recognition by Hydrogen Bonding: Urea-Based Receptors. Chem. Soc. Rev. 2010, 39 (10), 3889–3915. 10.1039/b822552b. 20818452

[ref15] Manna U.; Das G. An Overview of Anion Coordination by Hydroxyl, Amine and Amide Based Rigid and Symmetric Neutral Dipodal Receptors. Coord. Chem. Rev. 2021, 427, 213547 10.1016/j.ccr.2020.213547.

[ref16] Kundu S.; Egboluche T. K.; Hossain M. A. Urea- and Thiourea-Based Receptors for Anion Binding. Acc. Chem. Res. 2023, 56 (11), 1320–1329. 10.1021/acs.accounts.2c00701. 36913317

[ref17] Picci G.; Montis R.; Lippolis V.; Caltagirone C. Squaramide-Based Receptors in Anion Supramolecular Chemistry: Insights into Anion Binding, Sensing, Transport and Extraction. Chem. Soc. Rev. 2024, 53, 3952–3975. 10.1039/D3CS01165H. 38465875

[ref18] Řezanková M.; Budka J.; Mikšátko J.; Eigner V.; Císařová I.; Cuřínová P.; Lhoták P. Anion Receptors Based on Intramolecularly Bridged Calix[4]Arenes Bearing Ureido Functions. Tetrahedron 2017, 73 (6), 742–749. 10.1016/j.tet.2016.12.054.

[ref19] Van Craen D.; Kalarikkal M. G.; Holstein J. J. A Charge-Neutral Self-Assembled L2Zn2Helicate as Bench-Stable Receptor for Anion Recognition at Nanomolar Concentration. J. Am. Chem. Soc. 2022, 144 (39), 18135–18143. 10.1021/jacs.2c08579. 36137546

[ref20] Mercer D. J.; Loeb S. J. Metal-Based Anion Receptors: An Application of Second-Sphere Coordination. Chem. Soc. Rev. 2010, 39 (10), 3612–3620. 10.1039/b926226c. 20617246

[ref21] Cai J.; Sessler J. L. Neutral CH and Cationic CH Donor Groups as Anion Receptors. Chem. Soc. Rev. 2014, 43 (17), 6198–6213. 10.1039/C4CS00115J. 24871377

[ref22] Abdurakhmanova E. R.; Mondal D.; Jędrzejewska H.; Cmoch P.; Danylyuk O.; Chmielewski M. J.; Szumna A. Supramolecular Umpolung: Converting Electron-Rich Resorcin[4]Arenes into Potent CH-Bonding Anion Receptors and Transporters. Chem 2024, 10 (6), 1910–1924. 10.1016/j.chempr.2024.03.003.

[ref23] Wang D. X.; Wang M. X. Exploring Anion-Π Interactions and Their Applications in Supramolecular Chemistry. Acc. Chem. Res. 2020, 53 (7), 1364–1380. 10.1021/acs.accounts.0c00243. 32559061

[ref24] Brown A.; Beer P. D. Halogen Bonding Anion Recognition. Chem. Commun. 2016, 52 (56), 8645–8658. 10.1039/C6CC03638D. 27273600

[ref25] Tepper R.; Schubert U. S. Halogen Bonding in Solution: Anion Recognition, Templated Self-Assembly, and Organocatalysis. Angew. Chem., Int. Ed. 2018, 57 (21), 6004–6016. 10.1002/anie.201707986. 29341377

[ref26] Hein R.; Beer P. D. Halogen Bonding and Chalcogen Bonding Mediated Sensing. Chem. Sci. 2022, 13 (24), 7098–7125. 10.1039/D2SC01800D. 35799814 PMC9214886

[ref27] Lim J. Y. C.; Beer P. D. Sigma-Hole Interactions in Anion Recognition. Chem 2018, 4 (4), 731–783. 10.1016/j.chempr.2018.02.022.

[ref28] Elmes R. B. P.; Jolliffe K. A. Anion Recognition by Cyclic Peptides. Chem. Commun. 2015, 51 (24), 4951–4968. 10.1039/C4CC10095F. 25647007

[ref29] Jing L.; Deplazes E.; Clegg J. K.; Wu X. A Charge-Neutral Organic Cage Selectively Binds Strongly Hydrated Sulfate Anions in Water. Nat. Chem. 2024, 16, 335–342. 10.1038/s41557-024-01457-5. 38351381

[ref30] Li H.; Kou L.; Liang L.; Li B.; Zhao W.; Yang X. J.; Wu B. Anion-Coordination-Driven Single-Double Helix Switching and Chiroptical Molecular Switching Based on Oligoureas. Chem. Sci. 2022, 13 (17), 4915–4921. 10.1039/D2SC00876A. 35655878 PMC9067589

[ref31] Narikiyo H.; Gon M.; Tanaka K.; Chujo Y. Development of Fluorescence Sensors for Quantifying Anions Based on Polyhedral Oligomeric Silsesquioxane That Contains Flexible Side Chains with Urea Structures. Polym. J. 2024, 56, 661–666. 10.1038/s41428-024-00909-6.

[ref32] Park Y.; Harper K. C.; Kuhl N.; Kwan E. E.; Liu R. Y.; Jacobsen E. N. Macrocyclic Bis-Thioureas Catalyze Stereospecific Glycosylation Reactions. Science 2017, 355 (6321), 162–166. 10.1126/science.aal1875. 28082586 PMC5671764

[ref33] Neri P.; Sessler J. L.; Wang M.-X. Calixarenes and Beyond; Neri P., Sessler J. L., Wang M.-X., Eds.; Springer, 2016.

[ref34] Rebilly J. N.; Colasson B.; Bistri O.; Over D.; Reinaud O. Biomimetic Cavity-Based Metal Complexes. Chem. Soc. Rev. 2015, 44 (2), 467–489. 10.1039/C4CS00211C. 25319612

[ref35] Coquière D.; Le Gac S.; Darbost U.; Sénèque O.; Jabin I.; Reinaud O. Biomimetic and Self-Assembled Calix[6]Arene-Based Receptors for Neutral Molecules. Org. Biomol. Chem. 2009, 7 (12), 2485–2500. 10.1039/b902456e. 19503918

[ref36] Danil De Namor A. F.; Aparicio-Aragon W.; Nwogu N.; El Gamouz A.; Piro O. E.; Castellano E. E. Calixarene and Resorcarene Based Receptors: From Structural and Thermodynamic Studies to the Synthesis of a New Mercury(II) Selective Material. J. Phys. Chem. B 2011, 115 (21), 6922–6934. 10.1021/jp110195f. 21545094

[ref37] Danil de Namor A. F.; Chahine S.; Castellano E. E.; Piro O. E. Thermodynamics of Host-Guest Interactions in Lower Rim Functionalized Calix[4]Arenes and Metal Cations: The Medium Effect. J. Phys. Chem. B 2004, 108 (31), 11384–11392. 10.1021/jp040069j.

[ref38] Kumar R.; Sharma A.; Singh H.; Suating P.; Kim H. S.; Sunwoo K.; Shim I.; Gibb B. C.; Kim J. S. Revisiting Fluorescent Calixarenes: From Molecular Sensors to Smart Materials. Chem. Rev. 2019, 119 (16), 9657–9721. 10.1021/acs.chemrev.8b00605. 31306015

[ref39] Sliwa W.; Girek T. Calixarene Complexes with Metal Ions. J. Inclusion Phenom. Macrocyclic Chem. 2010, 66 (1–2), 15–41. 10.1007/s10847-009-9678-7.

[ref40] Požar J.; Nikšić-Franjić I.; Cvetnić M.; Leko K.; Cindro N.; Pičuljan K.; Borilović I.; Frkanec L.; Tomišić V. Solvation Effect on Complexation of Alkali Metal Cations by a Calix[4]Arene Ketone Derivative. J. Phys. Chem. B 2017, 121 (36), 8539–8550. 10.1021/acs.jpcb.7b05093. 28805386

[ref41] Požar J.; Cvetnić M.; Usenik A.; Cindro N.; Horvat G.; Leko K.; Modrušan M.; Tomišić V. The Role of Triazole and Glucose Moieties in Alkali Metal Cation Complexation by Lower-Rim Tertiary-Amide Calix[4]Arene Derivatives. Molecules 2022, 27 (2), 470 10.3390/molecules27020470. 35056784 PMC8780480

[ref42] Leko K.; Usenik A.; Cindro N.; Modrušan M.; Požar J.; Horvat G.; Stilinović V.; Hrenar T.; Tomišić V. Enhancing the Cation-Binding Ability of Fluorescent Calixarene Derivatives: Structural, Thermodynamic, and Computational Studies. ACS Omega 2023, 8 (45), 43074–43087. 10.1021/acsomega.3c06509. 38024729 PMC10652827

[ref43] Scheerder J.; Fochi M.; Engbersen J. F. J.; Reinhoudt D. N. Urea-Derivatized p-tert-Butylcalix[4]arenes: Neutral Ligands for Selective Anion Complexation. J. Org. Chem. 1994, 59 (25), 7815–7820. 10.1021/jo00104a044.

[ref44] Scheerder J.; Engbersen J. F. J.; Casnati A.; Ungaro R.; Reinhoudt D. N. Complexation of Halide Anions and Tricarboxylate Anions by Neutral Urea-Derivatized p-Tert-Butylcalix[6]Arenes. J. Org. Chem. 1995, 60, 6448–6454. 10.1021/jo00125a035.

[ref45] Horáčková T.; Budka J.; Eigner V.; Chung W.-S.; Cuřínová P.; Lhoták P. Chiral Anion Recognition Using Calix[4]Arene-Based Ureido Receptors in a *1,3-Alternate* Conformation. Beilstein J. Org. Chem. 2020, 16, 2999–3007. 10.3762/bjoc.16.249. 33363668 PMC7736684

[ref46] Schazmann B.; Diamond D. Improved Nitrate Sensing Using Ion Selective Electrodes Based on Urea-Calixarene Ionophores. New J. Chem. 2007, 31 (4), 587–592. 10.1039/B702841P.

[ref47] Babu J. N.; Bhalla V.; Kumar M.; Mahajan R. K.; Puri R. K. A Chloride Selective Sensor Based on a Calix[4]Arene Possessing a Urea Moiety. Tetrahedron Lett. 2008, 49 (17), 2772–2775. 10.1016/j.tetlet.2008.02.133.

[ref48] Babu J. N.; Bhalla V.; Kumar M.; Puri R. K.; Mahajan R. K. Chloride Ion Recognition Using Thiourea/Urea Based Receptors Incorporated into 1,3-Disubstituted Calix[4]Arenes. New J. Chem. 2009, 33 (3), 675–681. 10.1039/b816610b.

[ref49] Sansone F.; Chierici E.; Casnati A.; Ungaro R. Thiourea-Linked Upper Rim Calix[4]Arene Neoglycoconjugates: Synthesis, Conformations and Binding Properties. Org. Biomol. Chem. 2003, 1 (10), 1802–1809. 10.1039/B301595E. 12926373

[ref50] Bozkurt S.; Turkmen M. B. Synthesis of Calix[4]Arene-Based Thiourea Derivatives for Extraction of Toxic Dichromate and Arsenate Ions. Polycyclic Aromat. Compd. 2018, 38 (2), 157–167. 10.1080/10406638.2016.1174719.

[ref51] Durmaz M.; Acikbas Y.; Bozkurt S.; Capan R.; Erdogan M.; Ozkaya C. A Novel Calix[4]Arene Thiourea Decorated with 2-(2-Aminophenyl)Benzothiazole Moiety as Highly Selective Chemical Gas Sensor for Dichloromethane Vapor. ChemistrySelect 2021, 6 (19), 4670–4676. 10.1002/slct.202100631.

[ref52] Hamon M.; Ménand M.; Le Gac S.; Luhmer M.; Dalla V.; Jabin I. Calix[6]Tris(Thio)Ureas: Heteroditopic Receptors for the Cooperative Binding of Organic Ion Pairs. J. Org. Chem. 2008, 73 (18), 7067–7071. 10.1021/jo800712q. 18712925

[ref53] Ménand M.; Jabin I. Synthesis of the First Calix[6]Crypturea via a Versatile Tris-Azide Precursor. Org. Lett. 2009, 11 (3), 673–676. 10.1021/ol8027384. 19138121

[ref54] Moerkerke S.; Wouters J.; Jabin I. Selective Recognition of Phosphatidylcholine Lipids by a Biomimetic Calix[6]Tube Receptor. J. Org. Chem. 2015, 80 (17), 8720–8726. 10.1021/acs.joc.5b01531. 26258943

[ref55] Grauwels G.; Valkenier H.; Davis A. P.; Jabin I.; Bartik K. Repositioning Chloride Transmembrane Transporters: Transport of Organic Ion Pairs. Angew. Chem., Int. Ed. 2019, 58 (21), 6921–6925. 10.1002/anie.201900818. 30925004

[ref56] Bregović N.; Cindro N.; Frkanec L.; Tomišić V. Complexation of Fluoride Anion and Its Ion Pairs with Alkali Metal Cations by Tetra-Substituted Lower Rim Calix[4]Arene Tryptophan Derivative. Supramol. Chem. 2016, 28 (5–6), 608–615. 10.1080/10610278.2016.1154147.

[ref57] Cera G.; Bazzoni M.; Andreoni L.; Cester Bonati F.; Massera C.; Silvi S.; Credi A.; Secchi A.; Arduini A. Thioureidocalix[6]Arenes Pseudorotaxanes. Eur. J. Org Chem. 2021, 2021 (42), 5788–5798. 10.1002/ejoc.202101080.

[ref58] Marcos P. M.; Teixeira F. A.; Segurado M. A. P.; Ascenso J. R.; Bernardino R. J.; Michel S.; Hubscher-Bruder V. Bidentate Urea Derivatives of P-Tert-Butyldihomooxacalix[4]Arene: Neutral Receptors for Anion Complexation. J. Org. Chem. 2014, 79 (2), 742–751. 10.1021/jo4026012. 24358937

[ref59] Marcos P. M.; Teixeira F. A.; Segurado M. A. P.; Ascenso J. R.; Bernardino R. J.; Brancatelli G.; Geremia S. Synthesis and Anion Binding Properties of New Dihomooxacalix[4]Arene Diurea and Dithiourea Receptors. Tetrahedron 2014, 70 (37), 6497–6505. 10.1016/j.tet.2014.07.020.

[ref60] Teixeira F. A.; Marcos P. M.; Ascenso J. R.; Brancatelli G.; Hickey N.; Geremia S. Selective Binding of Spherical and Linear Anions by Tetraphenyl(Thio)Urea-Based Dihomooxacalix[4]Arene Receptors. J. Org. Chem. 2017, 82 (21), 11383–11390. 10.1021/acs.joc.7b01801. 28990384

[ref61] Augusto A. S.; Miranda A. S.; Ascenso J. R.; Miranda M. Q.; Félix V.; Brancatelli G.; Hickey N.; Geremia S.; Marcos P. M. Anion Recognition by Partial Cone Dihomooxacalix[4]Arene-Based Receptors Bearing Urea Groups: Remarkable Affinity for Benzoate Ion. Eur. J. Org Chem. 2018, 2018 (41), 5657–5667. 10.1002/ejoc.201800880.

[ref62] Miranda A. S.; Marcos P. M.; Ascenso J. R.; Berberan-Santos M. N.; Schurhammer R.; Hickey N.; Geremia S. Dihomooxacalix[4]Arene-Based Fluorescent Receptors for Anion and Organic Ion Pair Recognition. Molecules 2020, 25 (20), 4708 10.3390/molecules25204708. 33066580 PMC7587342

[ref63] Amendola V.; Esteban-Gómez D.; Fabbrizzi L.; Licchelli M. What Anions Do to N-H-Containing Receptors. Acc. Chem. Res. 2006, 39 (5), 343–353. 10.1021/ar050195l. 16700533

[ref64] Kim Y. J.; Kwak H.; Lee S. J.; Lee J. S.; Kwon H. J.; Nam S. H.; Lee K.; Kim C. Urea/Thiourea-Based Colorimetric Chemosensors for the Biologically Important Ions: Efficient and Simple Sensors. Tetrahedron 2006, 62 (41), 9635–9640. 10.1016/j.tet.2006.07.081.

[ref65] Amendola V.; Fabbrizzi L.; Mosca L.; Schmidtchen F. P. Urea-Squaramide-and Sulfonamide-Based Anion Receptors: A Thermodynamic Study. Chem.—Eur. J. 2011, 17 (21), 5972–5981. 10.1002/chem.201003411. 21472802

[ref66] Caltagirone C.; Bazzicalupi C.; Isaia F.; Light M. E.; Lippolis V.; Montis R.; Murgia S.; Olivari M.; Picci G. A New Family of Bis-Ureidic Receptors for Pyrophosphate Optical Sensing. Org. Biomol. Chem. 2013, 11 (15), 2445–2451. 10.1039/c3ob27244c. 23385302

[ref67] Barišić D.; Cindro N.; Kulcsár M. J.; Tireli M.; Užarević K.; Bregović N.; Tomišić V. Protonation and Anion Binding Properties of Aromatic Bis-Urea Derivatives—Comprehending the Proton Transfer. Chem.—Eur. J. 2019, 25 (18), 4695–4706. 10.1002/chem.201805633. 30657616

[ref68] Barišić D.; Cindro N.; Vidović N.; Bregović N.; Tomišić V. Protonation and Anion-Binding Properties of Aromatic Sulfonylurea Derivatives. RSC Adv. 2021, 11 (39), 23992–24000. 10.1039/D1RA04738H. 35479025 PMC9039416

[ref69] Kütt A.; Tshepelevitsh S.; Saame J.; Lõkov M.; Kaljurand I.; Selberg S.; Leito I. Strengths of Acids in Acetonitrile. Eur. J. Org Chem. 2021, 2021 (9), 1407–1419. 10.1002/ejoc.202001649.

[ref70] Bregović N.; Cindro N.; Bertoša B.; Barišić D.; Frkanec L.; Užarević K.; Tomišić V. Dehydroacetic Acid Derivatives Bearing Amide or Urea Moieties as Effective Anion Receptors. Chem.—Eur. J. 2017, 23 (43), 10396–10406. 10.1002/chem.201701677. 28493492

[ref71] Cvetnić M.; Cindro N.; Topić E.; Bregović N.; Tomišić V. Supramolecular Handshakes: Characterization of Urea-Carboxylate Interactions Within Calixarene Frameworks. ChemPlusChem 2024, 89, e202400130 10.1002/cplu.202400130. 38526220

[ref72] Schwesinger R.; Schlemper H.; Hasenfratz C.; Willaredt J.; Dambacher T.; Breuer T.; Ottaway C.; Fletschinger M.; Boele J.; Fritz H.; Putzas D.; Rotter H. W.; Bordwell F. G.; Satish A. V.; Ji G. Z.; Peters E. M.; Peters K.; Von Schnering H. G.; Walz L. Extremely Strong, Uncharged Auxiliary Bases; Monomeric and Polymer-Supported Polyaminophosphazenes (P2-P5). Liebigs Ann. 1996, 1996 (7), 1055–1081. 10.1002/jlac.199619960705.

[ref73] Frassineti C.; Ghelli S.; Gans P.; Sabatini A.; Moruzzi M. S.; Vacca A. Nuclear Magnetic Resonance as a Tool for Determining Protonation Constants of Natural Polyprotic Bases in Solution. Anal. Biochem. 1995, 231, 374–382. 10.1006/abio.1995.9984. 8594988

[ref74] Bordwell F. G. Equilibrium Acidities in Dimethyl Sulfoxide Solution. Acc. Chem. Res. 1988, 21, 456–463. 10.1021/ar00156a004.

[ref75] Kaljurand I.; Kütt A.; Sooväli L.; Rodima T.; Mäemets V.; Leito I.; Koppel I. A. Extension of the Self-Consistent Spectrophotometric Basicity Scale in Acetonitrile to a Full Span of 28 PKa Units: Unification of Different Basicity Scales. J. Org. Chem. 2005, 70 (3), 1019–1028. 10.1021/jo048252w. 15675863

[ref76] Tshepelevitsh S.; Kütt A.; Lõkov M.; Kaljurand I.; Saame J.; Heering A.; Plieger P. G.; Vianello R.; Leito I. On the Basicity of Organic Bases in Different Media. Eur. J. Org Chem. 2019, 2019 (40), 6735–6748. 10.1002/ejoc.201900956.

[ref77] Bregović N.; Cindro N.; Frkanec L.; Užarević K.; Tomišić V. Thermodynamic Study of Dihydrogen Phosphate Dimerisation and Complexation with Novel Urea- and Thiourea-Based Receptors. Chem.—Eur. J. 2014, 20 (48), 15863–15871. 10.1002/chem.201404091. 25283787

[ref78] Horvat G.; Tarana S.; Vidović N.; Cindro N.; Speranza G.; Tomišić V. Thermodynamic and MD Studies of Anion Complexation by Cyclopentaleucine in Acetonitrile and Dimethyl Sulfoxide. J. Mol. Liq. 2021, 340, 116848 10.1016/j.molliq.2021.116848.

[ref79] Rinkovec T. Thermodynamic and Structural Studies of the Complexation of Homocyclopeptides with Halide and Oxoanions in Acetonitrile, Ph.D. Thesis, Faculty of Science, University of Zagreb, Zagreb, 2018.

[ref80] Danil de Namor A. F.; Shehab M. Recognition of Biologically and Environmentally Important Phosphate Anions by Calix[4]Pyrrole: Thermodynamic Aspects. J. Phys. Chem. A 2004, 108 (35), 7324–7330. 10.1021/jp031343x.

[ref81] Danil De Namor A. F.; Shehab M.; Abbas I.; Withams M. V.; Zvietcovich-Guerra J. New Insights on Anion Recognition by Isomers of a Calix Pyrrole Derivative. J. Phys. Chem. B 2006, 110 (25), 12653–12659. 10.1021/jp060859o. 16800598

[ref82] Danil De Namor A. F.; Chaaban J. K.; Abbas I. Cation/Anion Recognition by a Partially Substituted Lower Rim Calix[4]Arene Hydroxyamide, a Ditopic Receptor. J. Phys. Chem. A 2006, 110 (31), 9575–9584. 10.1021/jp062154s. 16884190

[ref83] Barišić D.; Tomišić V.; Bregović N. Acid-Base Properties of Phosphoric and Acetic Acid in Aprotic Organic Solvents - A Complete Thermodynamic Characterisation. Anal. Chim. Acta 2019, 1046, 77–92. 10.1016/j.aca.2018.09.026. 30482305

[ref84] Santos M. M.; Marques I.; Carvalho S.; Moiteiro C.; Félix V. Recognition of Bio-Relevant Dicarboxylate Anions by an Azacalix[2]Arene[2]Triazine Derivative Decorated with Urea Moieties. Org. Biomol. Chem. 2015, 13 (10), 3070–3085. 10.1039/C4OB02283A. 25624063

[ref85] Gans P.; Sabatini A.; Vacca A. Investigation of Equilibria in Solution. Determination of Equilibrium Constants with the HYPERQUAD Suite of Programs. Talanta 1996, 43, 1739–1753. 10.1016/0039-9140(96)01958-3. 18966661

[ref86] Rigaku Oxford Diffraction, Gemini CCD System, CrysAlis Pro Software, Version 171.41.93a, 2020.

[ref87] Sheldrick G. M. SHELXT - Integrated Space-Group and Crystal-Structure Determination. Acta Crystallogr., Sect. A: Found. Adv. 2015, 71 (1), 3–8. 10.1107/S2053273314026370. 25537383 PMC4283466

[ref88] Sheldrick G. M. Crystal Structure Refinement with SHELXL. Acta Crystallogr., Sect. C: Struct. Chem. 2015, 71 (1), 3–8. 10.1107/S2053229614024218. 25567568 PMC4294323

[ref89] Dolomanov O. V.; Bourhis L. J.; Gildea R. J.; Howard J. A. K.; Puschmann H. OLEX2: A Complete Structure Solution, Refinement and Analysis Program. J. Appl. Crystallogr. 2009, 42 (2), 339–341. 10.1107/S0021889808042726.

