# Supplementary material for: Thermodynamics of Anion Binding by (Thio)ureido-calix[4]arene Derivatives in Acetonitrile
Source: ACS Phys Chem Au. 2024 Oct 15;4(6):773–86. doi: 10.1021/acsphyschemau.4c00077 (PMC11613299; doi:10.1021/acsphyschemau.4c00077)
Supplement: Supplementary file 1 — pg4c00077_si_001.pdf [file pg4c00077_si_001.pdf]

Thermodynamics of anion binding by (thio)ureido-calix[4]arene derivatives in acetonitrile

Marija Cvetnić, Nikola Cindro, Nikola Bregović*, and Vladislav Tomišić

Department of Chemistry, Faculty of Science, University of Zagreb, Horvatovac 102a, 10000 Zagreb, Croatia

*Correspondence: nbregovic@chem.pmf.hr

SUPPORTING INFORMATION

CONTENTS:

Calixarene 3 – synthesis and characterization **3**

Proof of absence of self-association of 3 in MeCN **7**

Assignment of proton NMR signals of receptors 1 and 2 .... **9**

Acidity of receptors **10**

Dealing with high values of protonation constants in HYPNMR **10**

Results **12**

Anion-binding investigation **19**

Chloride **19**

*Complexation of chloride with **1*** 19

*Complexation of chloride with **2*** 22

*Complexation of chloride with **3*** 25

Hydrogen sulfate **29**

*Complexation of hydrogen sulfate with **1*** 29

*Complexation of hydrogen sulfate with **2*** 31

*Complexation of hydrogen sulfate with **3*** 33

Dihydrogen phosphate **37**

*Complexation of dihydrogen phosphate with **1*** 37

*Complexation of dihydrogen phosphate with **2*** 41

*Complexation of dihydrogen phosphate with **3*** 45

Hydrogen pyrophosphate **49**

*Complexation of hydrogen pyrophosphate with **1*** 49

*Complexation of hydrogen pyrophosphate with **2*** 53

*Complexation of hydrogen pyrophosphate with **3*** 56

Comparison with other known relevant complexes 59

Fumarate **60**

Preparation of TBA_2fum salt 60

*Complexation of fumarate with **1*** 61

*Complexation of fumarate with **2*** 65

*Complexation of fumarate with **3*** 69

Test of binding of several acids (protonated forms of investigated anions) with 3 .... **74**

Acetate **75**

*Complexation of acetate with **3*** 75

Benzoate **80**

*Complexation of benzoate with **1*** 80

*Complexation of benzoate with **2*** 83

Homoconjugation of benzoate 85

*Complexation of benzoate with **3*** 87

References **96**

Calixarene 3 – synthesis and characterization

All chemicals and solvents were, at least, reagent grade. They were used without further purification and were purchased from commercial sources. NMR spectra were recorded on a Bruker Ascend 400 MHz spectrometer in CD_3CN. Chemical shifts are reported in ppm and referenced to TMS. HRMS analyses were carried out on Q Exactive™ Plus Hybrid Quadrupole-Orbitrap™ Mass Spectrometer (Electrospray Ionization).

Synthesis of macrocycle **3** was performed by applying procedures used in the synthesis of macrocycle **2** (Scheme S1: from **P** to **Q3**) which is described in our previous work.[1]

The last step of the synthesis of **3** (Scheme S1: from **Q3** to **3**) was done in the following way. To a solution of compound **Q3** (500 mg, 0.517 mmol) and DIPEA (450 µL, 2.59 mmol) in dry CH_2Cl_2 (20 mL), phenyl isothiocyanate (218 µL, 2.07 mmol) was added. The reaction solution was mixed for 29 h at 25 °C under Ar atmosphere and evaporated under reduced pressure. The crude product was dissolved in boiling ethanol (30 mL), followed by cooling to 25 °C. After a few days, the resulting crystals were filtered and washed with ethanol. Analytically pure white solid product was obtained in 36 % (256 mg) yield. The structure of final host molecule (**3**) was confirmed using HRMS, NMR and SCXRD techniques (Figures S1−S3, Table S1).

^{1}H NMR (400 MHz, CD_3CN) δ 8.40 (s, 4H), 7.34 (t + d, J_1 = 14.9 Hz, J_2 = 8 Hz, 16H), 7.22 (t, J = 6.9 Hz, 4H), 7.15 (s, 4H), 7.00 (s, 8H), 4.36 (d, J = 12.4 Hz, 4H), 4.16 (s = t + q, 16H), 3.20 (d, 12.4 Hz, 4H), 1.14 (s, 36H); **^{13}C NMR** (101 MHz, CD_3CN) δ 182.38, 161.82, 154.29, 146.43, 134.71, 130.29, 126.94, 126.57, 125.95, 73.42, 46.11, 34.76, 31.76, 31.64; **HRMS** m/z $[M{=}C_{80}H_{96}N_8O_4S_4 + H]^+$ calculated = 1361.6510, found = 1361.6530.

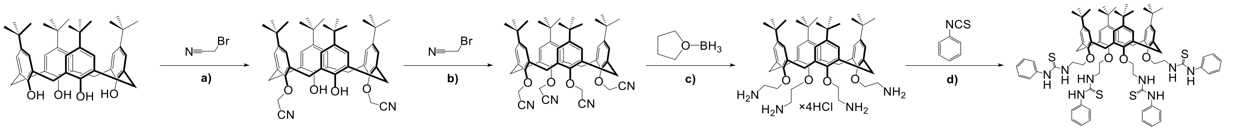

Scheme S1. Synthesis of calix[4]arene **3**. Conditions and yields: a) K_2CO_3, dry MeCN, reflux, 6 h (51 %), b) NaH, dry DMF, 75 °C, 30 h (43 %), c) dry THF, reflux, 16 h (87 %), d) DIPEA, dry DCM, 25 °C, 29 h (36 %).

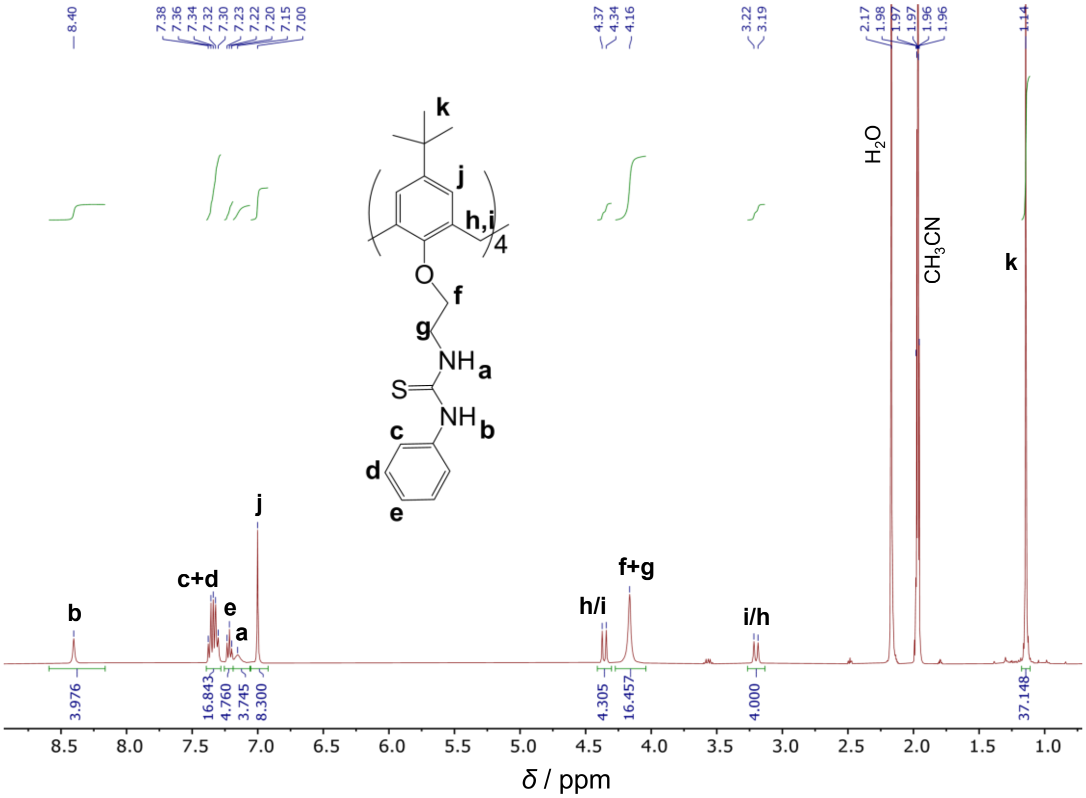

Figure S1. ^{1}H NMR spectrum of compound **3** in CD_3CN (400 MHz, 298 K).

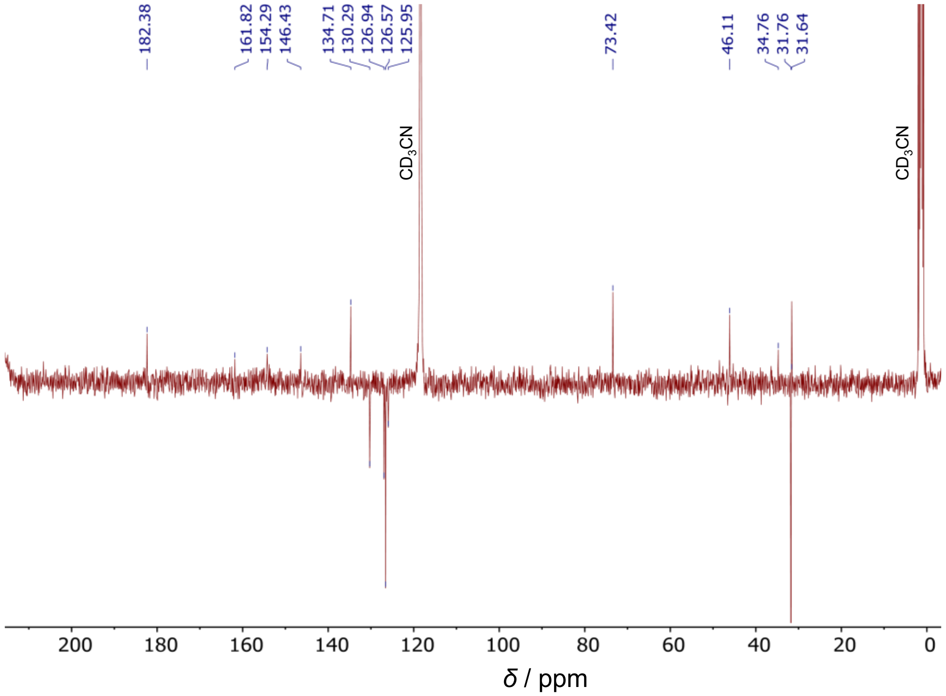

Figure S2. ^{13}C NMR spectrum of compound **3** in CD_3CN (101 MHz, 298 K).

(a) (b)

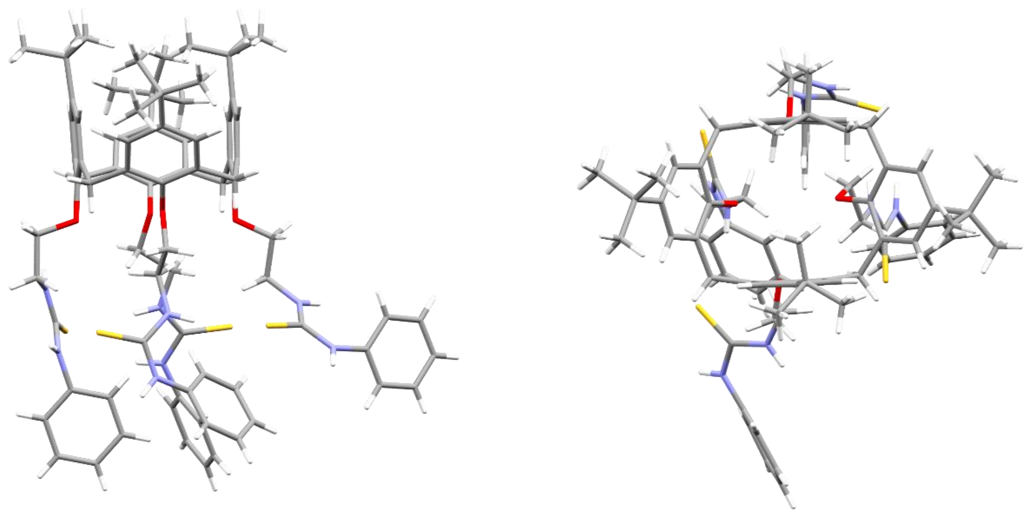

Figure S3. The molecular structure of **3** obtained by single-crystal X-ray diffraction: a) side view of the cavity, b) top view into the cavity. Solvent molecules are omitted for clarity.

Table S1. Experimental and crystallographic data for **3**·C_2H_5OH.

Compound	**3**·C_2H_5OH
Sum formula	$C_{82}H_{102}N_8O_5S_4$
M_r	1407.95
T/K	298
Crystal system	triclinic
Space group	$P\bar{1}$
a/Å	10.15885(4)
b/Å	11.78461(5)
c/Å	34.49059(12)
α/°	87.4749(3)
β/°	83.3967(3)
γ/°	76.1967(3)
V/Å^3	3982.71(3)
Z	2
ρ_{calc}/g cm^{-3}	1.174
μ/mm^{-1}	1.518
F(000)	1508.0
Crystal size/mm^3	0.25 × 0.2 × 0.15
Radiation	Cu Kα (λ = 1.54184)
2Θ range/°	5.16 to 159.91
Index ranges	$-12 \leq h \leq 12$, $-15 \leq k \leq 15$, $-43 \leq l \leq 43$
Reflections collected	177774
Independent reflections	17136 [R_{sigma} = 1.44%, R_{int} = 3.49 %]
Data/restraints/ parameters	17136/-/926
Goodness–of–fit on F^2, S^b	1.042
Final R and wR^c values [$I \geq 2\sigma(I)$]	R_1 = 6.74%, wR_2 = 19.81%
Final R and wR^c values [all data]	R_1 = 7.06%, wR_2 = 20.12%
Largest diff. peak/hole / e Å^{-3}	0.85/–0.73

$^a w = 1/[\sigma^2(F_o^2) + (g_1P)^2 + g_2P]$ where $P = (F_o^2 + 2F_c^2)/3$

$^b S = \{\Sigma[w(F_o^2 - F_c^2)^2]/(N_r - N_p)\}^{1/2}$ where N_r = number of independent reflections, N_p = number of refined parameters.

$^c R = \Sigma||F_o| - |F_c||/ \Sigma|F_o|$; $wR = \{\Sigma[w(F_o^2 - F_c^2)^2]/\Sigma[w(F_o^2)^2]\}^{1/2}$

Proof of absence of self-association of 3 in MeCN

Before investigating the thermodynamics of anion complexation with the prepared receptor **3** in acetonitrile, the possibility of its self-association was investigated by ^{1}H NMR spectroscopy (Figure S4). In the concentration range $5\times10^{-5} - 8\times10^{-3}$ mol dm^{-3}, the chemical shift changes are less than 0.01 ppm, which indicates the absence of **3** self-association under the conditions of interest.

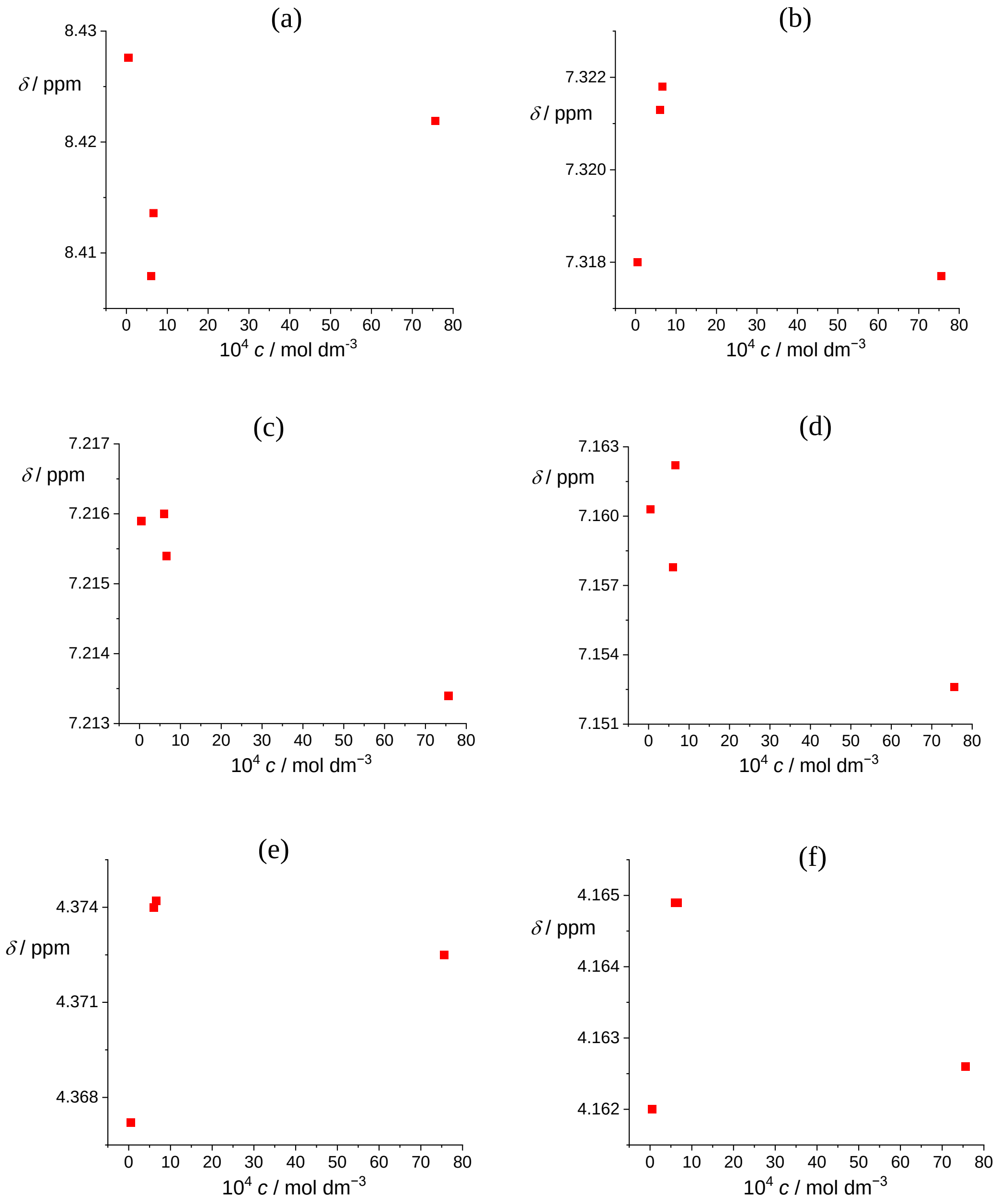

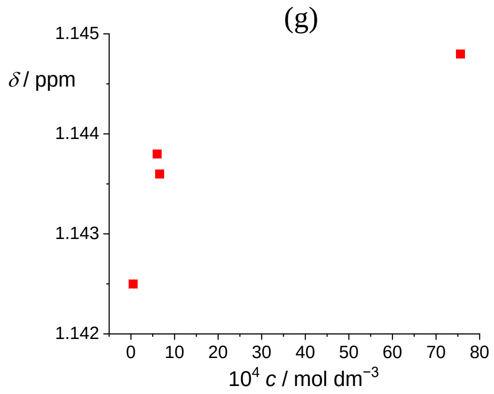

Figure S4. Chemical shifts of protons at **3**: a) „b“, b) „c“, c) „d“, d) „a“, e) „hi-left“, f) „f+g“, g) „k“) at different concentrations of **3** in CD_3CN at 25 °C. The assignment of protons is depicted in Figure S1. For the "j" and "hi-right" protons, the dependence of chemical shift on the concentration of **3** is even smaller than those shown for the other protons.

Assignment of proton NMR signals of receptors 1 and 2

(a)

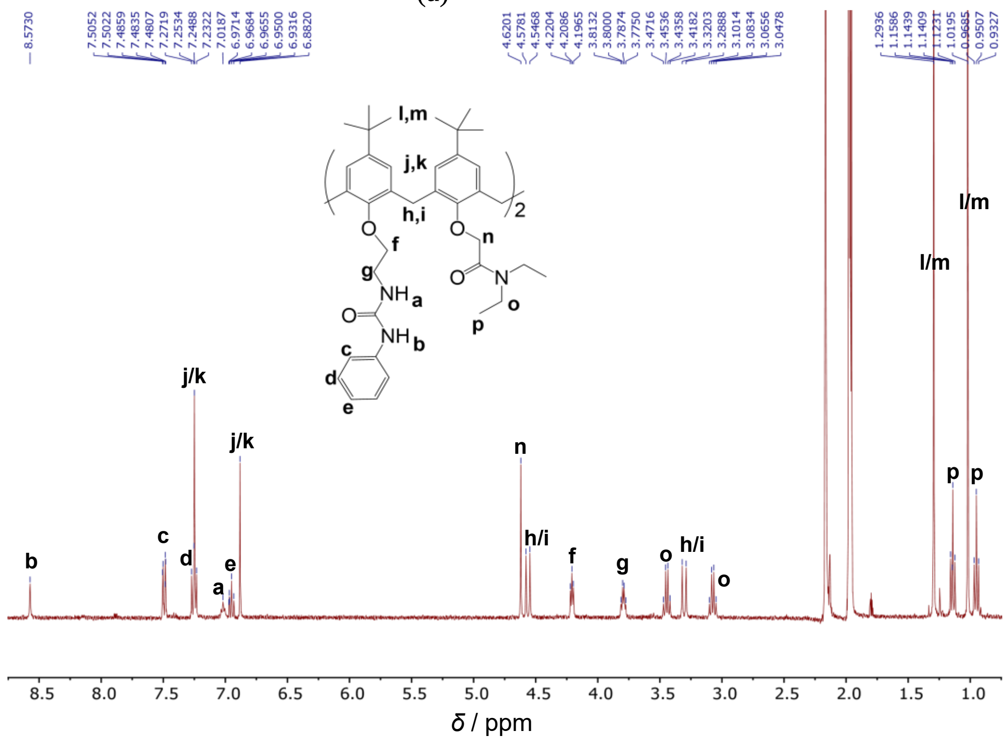

(b)

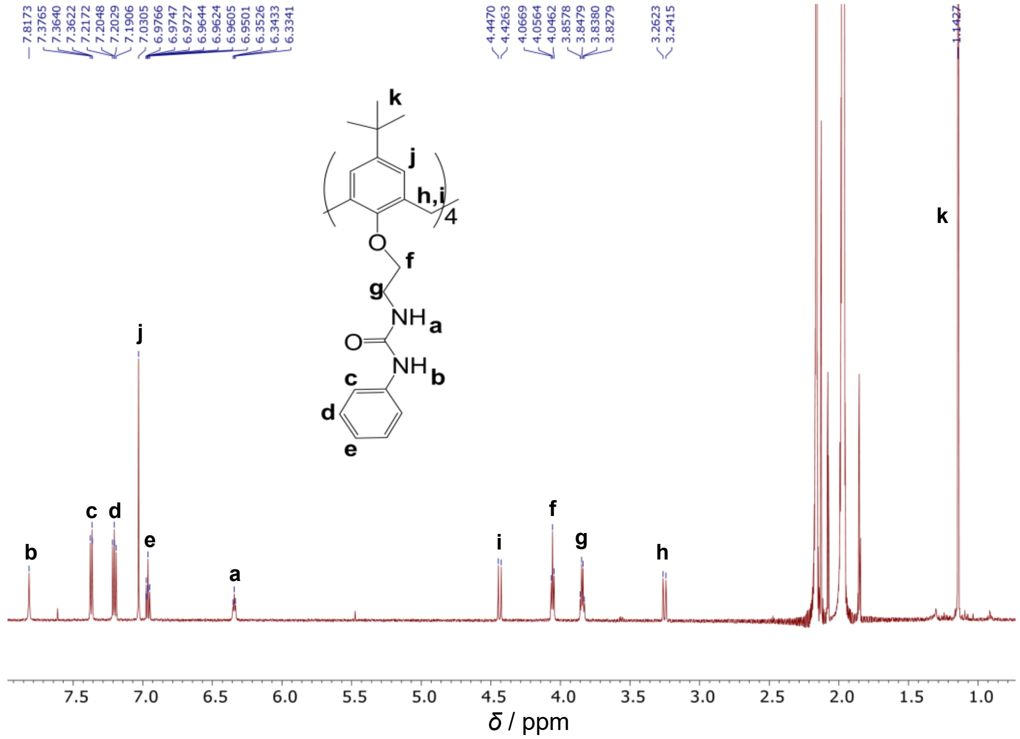

Figure S5. Assignment of proton NMR signals of receptors a) **1** (400 MHz) and b) **2** (600 MHz) in CD_3CN at 298 K.

Acidity of receptors

Dealing with high values of protonation constants in HYPNMR

The model used for fitting NMR titration data (depicted in Figures S6−S9) related to determination of acidity of calixarenes **1**, **2** and **3** in MeCN contained the following equilibria:

$$\text{calixarene}^{2-} + \text{H}^{+} \rightleftarrows \text{calixarene}^{-} \ldots K_1 \tag{1}$$

$$\text{calixarene}^{2-} + 2\text{H}^{+} \rightleftarrows \text{calixarene} \ldots \beta_2 \tag{2}$$

$$\text{base} + \text{H}^{+} \rightleftarrows \text{baseH}^{+} \ldots K_3 \tag{3}$$

$$\text{solvent} \rightleftarrows \text{solvent}^{-} + \text{H}^{+} \ldots K_4 \tag{4}$$

An estimation of autoprotolysis constant of MeCN (log $K_4 \approx 39$) was obtained from the work of Himmel et al.,[2] whereas the basicity evaluation of organic bases used in this investigation was downloaded from the self-consistent pK_a scale made, and constantly updated by the Leito group (log $K_3(P_2Et) = 32.94$, log $K_3(DBU) = 24.34$).[3–5]

The high values of association constants used in the model disabled the HYPNMR fitting procedure. To circumvent this problem, we reduced the association constants by the conversion constant of arbitrary value (K^{conv}) without chemical meaning. We introduced this constant through thermodynamic description of the protonation of base (eqs 3 and 5).

$$K_3 = \frac{[\text{baseH}^+]}{[\text{base}] \cdot [\text{H}^+]} = K_3^{\text{red}} \cdot K^{\text{conv}} \tag{5}$$

In the above equation K_3 is the real concentration association constant regarding the protonation of base, whereas K_3^{red} is the reduced concentration association constant.

If the method for the reduction of association constants for equilibria 1 – 4 is founded with the equation 5, then other relevant association constants can be written in the following way:

$$K_1 = \frac{[\text{calixarene}^-]}{[\text{calixarene}^{2-}] \cdot [\text{H}^+]} = K_1^{\text{red}} \cdot K^{\text{conv}} \tag{6}$$

$$\begin{aligned} \beta_2 = \frac{[\text{calixarene}]}{[\text{calixarene}^{2-}] \cdot [\text{H}^+]^2} &= \frac{[\text{calixarene}]}{[\text{calixarene}^{-}] \cdot [\text{H}^+]} \cdot \frac{[\text{calixarene}^-]}{[\text{calixarene}^{2-}] \cdot [\text{H}^+]} \\ &= K_2 \cdot K_1 = (K_2^{\text{red}} \cdot K^{\text{conv}}) \cdot (K_1^{\text{red}} \cdot K^{\text{conv}}) \\ &= \left(K_2^{\text{red}} \cdot K_1^{\text{red}}\right) \cdot (K^{\text{conv}} \cdot K^{\text{conv}}) = \beta_2^{\text{red}} \cdot (K^{\text{conv}})^2 \end{aligned} \tag{7}$$

$$K_4 = \frac{[\text{solvent}^-] \cdot [\text{H}^+]}{[\text{solvent}]} = \frac{K_4^{\text{red}}}{K^{\text{conv}}} \tag{8}$$

In logarithmic terms, appropriate for HYPNMR input, the equations 5 – 8 take the following forms:

$$\log K_1 = \log K_1^{\text{red}} + \log K^{\text{conv}} \tag{9}$$

$$\log \beta_2 = \log \beta_2^{\text{red}} + 2 \log K^{\text{conv}} \tag{10}$$

$$\log K_3 = \log K_3^{\mathrm{red}} + \log K^{\mathrm{conv}} \tag{11}$$

$$\log K_4 = \log K_4^{\mathrm{red}} - \log K^{\mathrm{conv}} \tag{12}$$

The concrete input and output values, i.e. the reduced and real values of the association constants, used in the determination of the acidity of calixarenes **1**, **2**, and **3** are given in Table S2. It should be noted that fitting procedure using reduced stability constants was repeated with several different values of K^{conv} (paying attention that stability constants attain high enough values after reduction; log β > 8) always providing the same refined values for the targeted beta, which approved the validity of the above-described method of dealing with high values of stability constants in HYPNMR.

The same procedure of dealing with high protonation constants was applied in handling the UV titration data regarding the acidity of **3**, using HypSpec program.

Table S2. Values of the association constants, for the equilibria defined through equations 1 – 4, used as parameters (fixed values) or obtained as results (refined values) of the HYPNMR-fitting of data that emerged from the titrations of calixarenes **1**, **2** and **3** with the appropriate base (P_2Et for **1** and DBU for **2** and **3**). All K_i constants are defined through equations 5 – 12.

	$\log K_\mathrm{i}$	**1**	**2**	**3**	Value fixed or refined during fitting?
"reduced" values	$\log K_1^{\mathrm{red}}$	11.9(3)	14.1(3)	12.94(4)	refined
	$\log \beta_2^{\mathrm{red}}$	24.6(1)	27.4(6)	24.44(1)	refined
	$\log K_3^{\mathrm{red}}$	13	13	13	fixed
	$\log K_4^{\mathrm{red}}$	−19.04	−27.66	−27.66	fixed
conversion factor	$\log K^{\mathrm{conv}}$	19.94	11.34	11.34	/
real values	$\log K_1$	31.8(3)	25.4(3)	24.28(4)	/
	$\log \beta_2$	64.5(1)	50.1(6)	47.12(1)	
	$\log K_3$	32.94	24.34	24.34	
	$\log K_4$	−39	−39	−39	

Uncertainties of the last digit are given in parentheses as standard deviation for the refined values.

Results

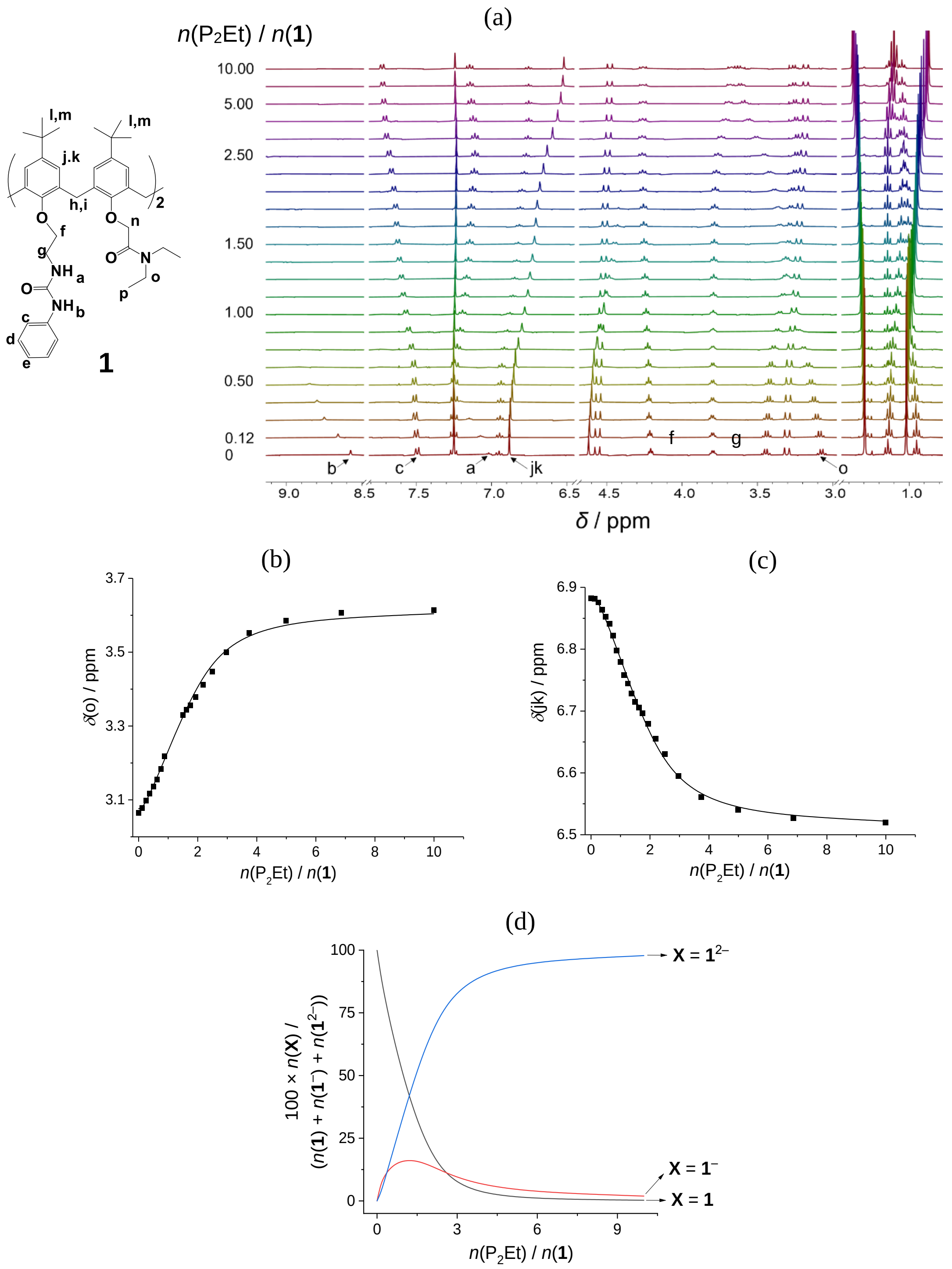

Figure S6. a) ^{1}H NMR spectroscopy titration of **1** (c = 8.00 ×10^{-4} mol dm^{-3}, V_0 = 500 µL) with P_2Et (c = 1.25×10^{-2} mol dm^{-3}) in CD_3CN at 25 °C. b), c) Experimental (■) and calculated (—) chemical shifts for selected nuclei at **1**. d) Distribution of (de)protonated species of **1** during the titration of **1** with P_2Et.

Table S3. Calculated ^1H NMR chemical shifts (in ppm) for **1** and its deprotonated forms in CD_3CN at 25 °C. Assignment of protons is depicted in Figure S5. Left/right assignments (= downfield/upfield, respectively) refer to the position of signal in NMR spectrum when assigning pairs of similar protons.

H	**1**	$\mathbf{1}^{-}$	$\mathbf{1}^{2-}$
c	7.4838	7.4748	7.7366
jk-right	6.8827	7.0775	6.51
o-right	3.0758	2.8794	3.6196
e	6.9711	6.8646	6.7362
o-left	3.4675	3.1497	3.2299
hi-right	3.3	3.2392	3.1544
lm-right	1.0182	1.1196	0.8658

(a)

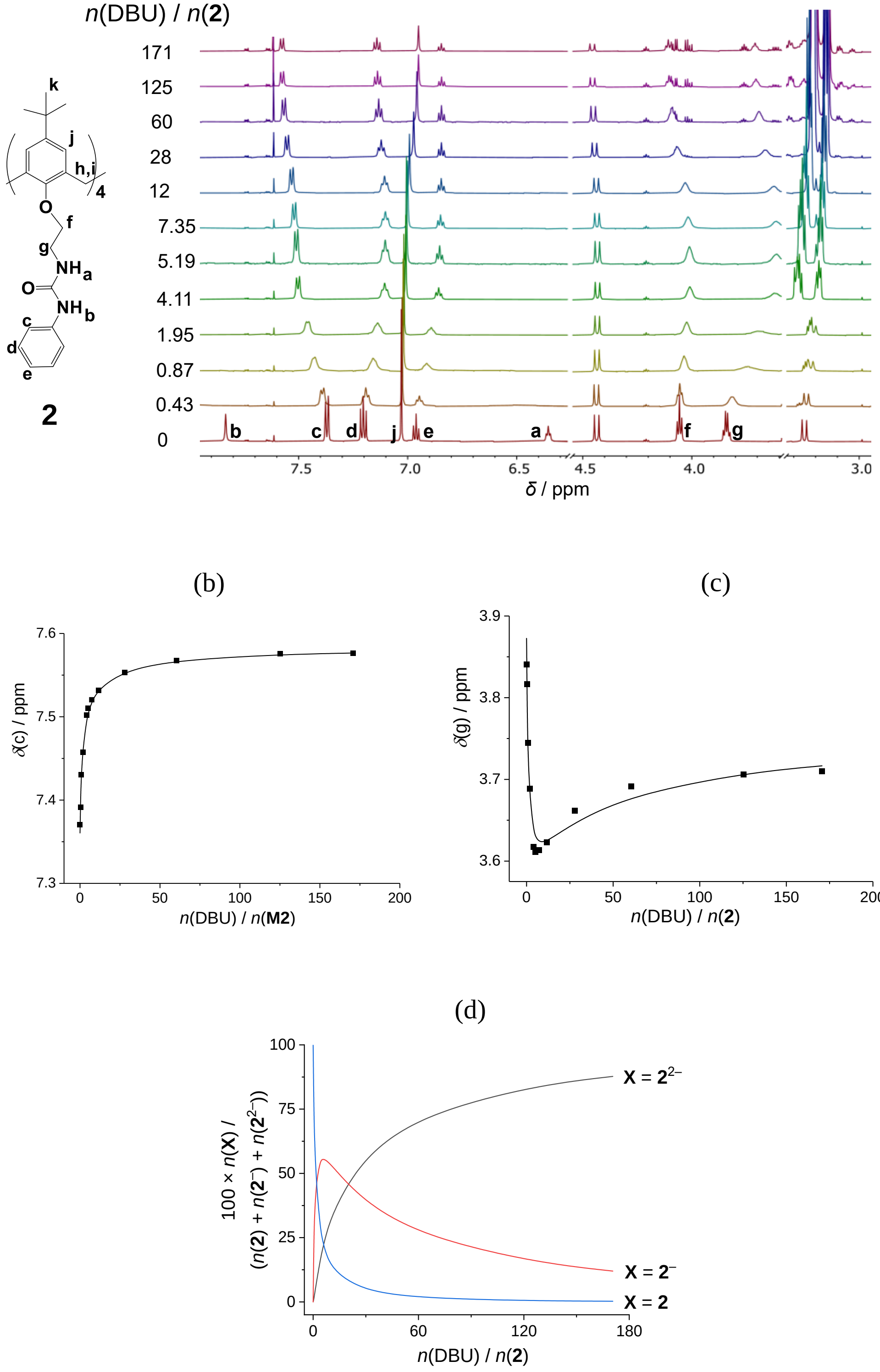

Figure S7. a) ^{1}H NMR spectroscopy titration of **2** (c = 6.94 ×10^{-5} mol dm^{-3}, V_0 = 500 μL) with DBU (c = 7.50×10^{-3} mol dm^{-3}) in CD_3CN at 25 °C. b), c) Experimental (■) and calculated (—) chemical shifts for selected nuclei at **2**. d) Distribution of (de)protonated species of **2** during the titration of **2** with DBU.

Table S4. Calculated ^{1}H NMR chemical shifts (in ppm) for **2** and its deprotonated forms in CD_3CN at 25 °C. Assignment of protons is depicted in Figure S5.

H	**2**	$\mathbf{2}^{-}$	$\mathbf{2}^{2-}$
c	7.3604	7.5399	7.5822
j	7.029	7.0191	6.9375
k	1.1423	1.1342	1.1217
d	7.216	7.0463	7.1559
e	6.9717	6.812	6.8511
f	4.0684	3.9528	4.1335
g	3.8726	3.4804	3.7485

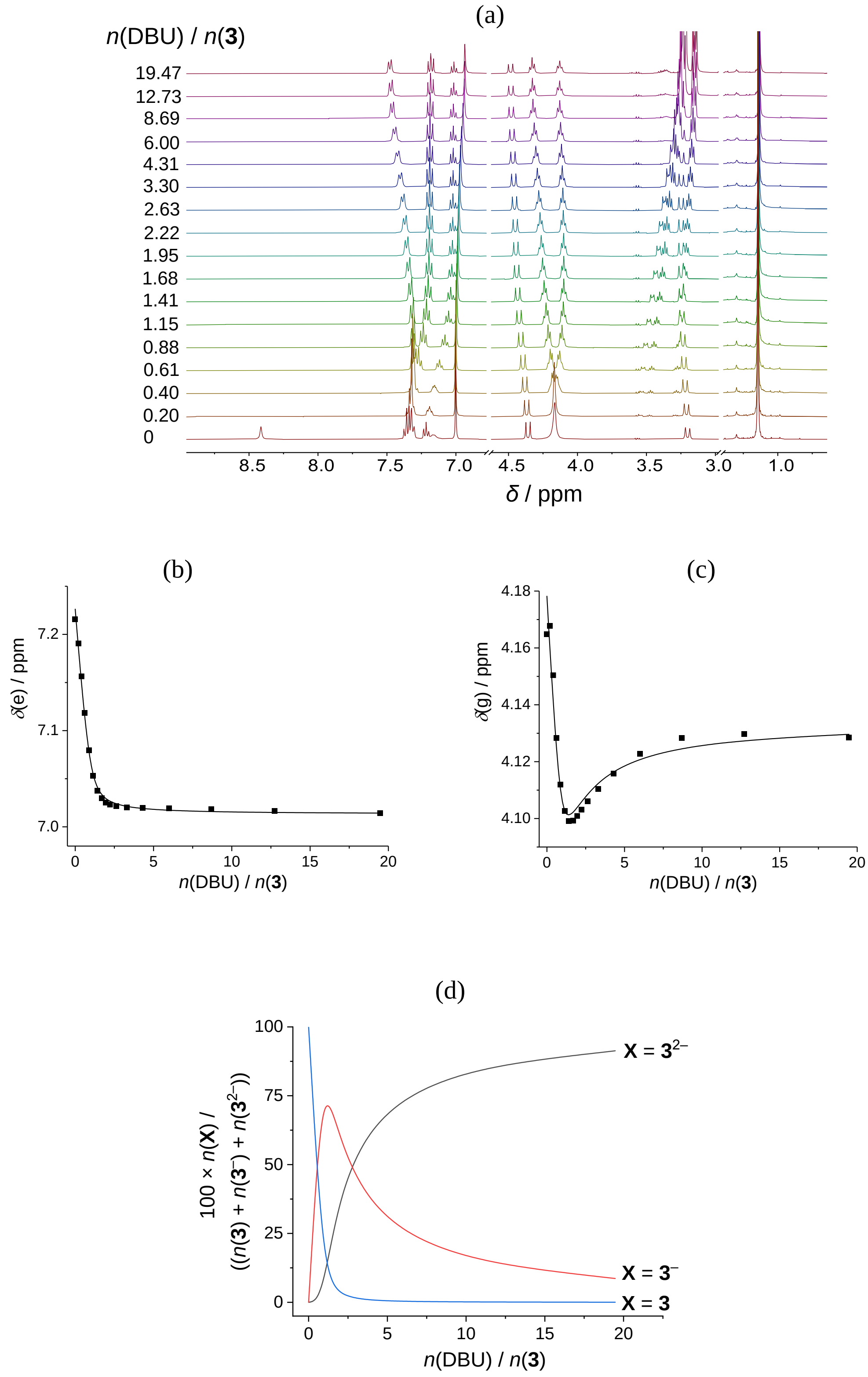

Figure S8. a) ^{1}H NMR spectroscopy titration of **3** (c = 6.62 ×10^{-4} mol dm^{-3}, V_0 = 450 μL) with DBU (c = 6.69×10^{-2} mol dm^{-3}) in CD_3CN at 25 °C. b), c) Experimental (■) and calculated (—) chemical shifts for selected nuclei at **3**. d) Distribution of (de)protonated species of **3** during the titration of **3** with DBU.

Table S5. Calculated ^{1}H NMR chemical shifts (in ppm) for **3** and its deprotonated forms in CD_3CN at 25 °C. Assignment of protons is depicted in Figure S1. Left/right assignments (= downfield/upfield, respectively) refer to the position of signal in NMR spectrum when assigning pairs of similar protons.

H	**3**	**3**$^{-}$	**3**$^{2-}$
c	7.3046	7.2741	7.4892
d	7.3641	7.1908	7.1839
e	7.2262	7.0257	7.013
j	7.0001	7.0096	6.9276
hi-left	4.3405	4.4097	4.4767
f	4.1589	4.2226	4.3446
g	4.1782	4.082	4.1341
hi-right	3.1839	3.2392	3.2237
k	1.1434	1.1472	1.1323

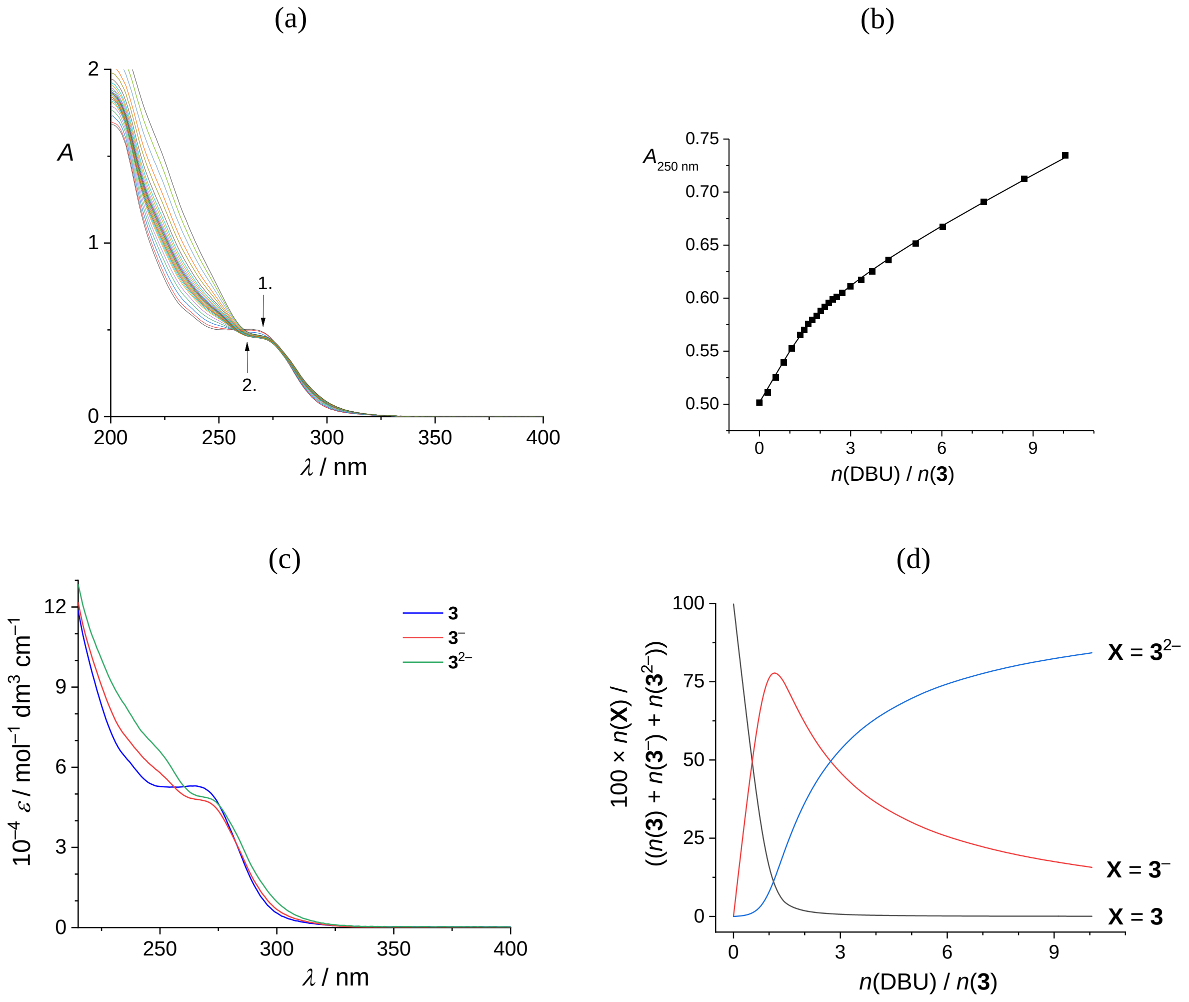

Figure S9. Spectrophotometric titration of **3** (c = 9,49×10^{-6} mol dm^{-3}, V_0 = 2,3 mL) with DBU (c = 3,91×10^{-4} mol dm^{-3}) in acetonitrile. l = 1 cm; ϑ = (25.0 ± 0.1) °C. a) The spectra corrected for dilution of **3**. b) Dependence of absorbance at 250 nm on n(DBU) / n(**3**) ratio. ■ Experimental; — calculated. c) Characteristic UV spectra of **3** and its (de)protonated forms. d) Distribution of (de)protonated forms of **3** during the titration with DBU.

Anion-binding investigation

Chloride

*Complexation of chloride with **1***

(a)

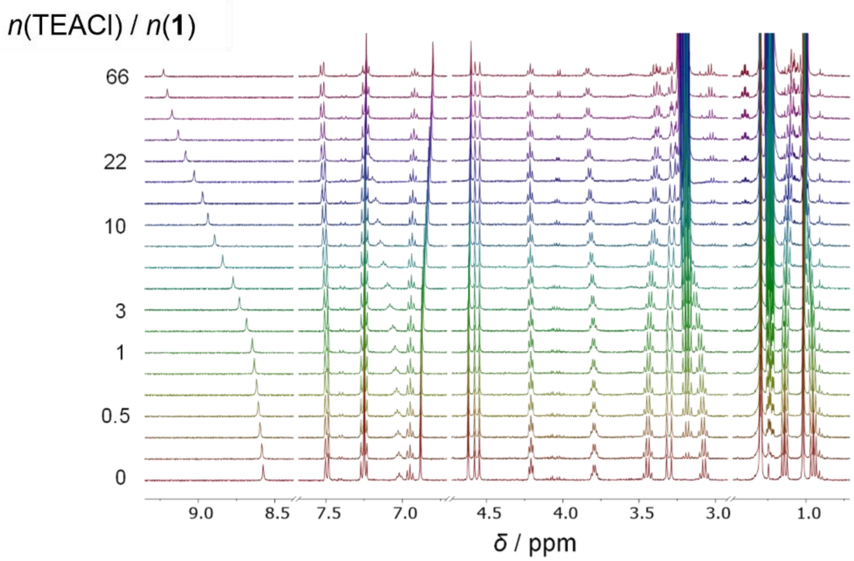

(b)

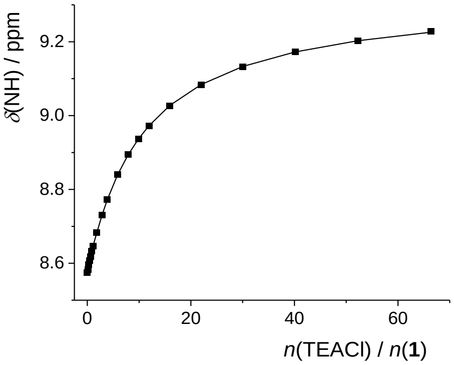

(c)

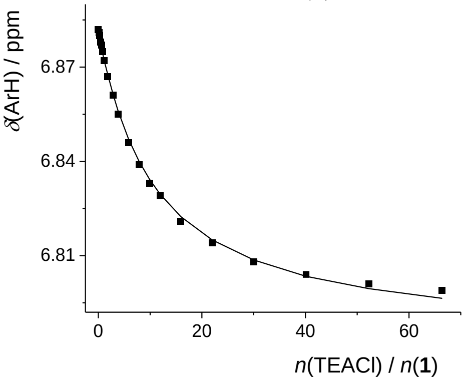

Figure S10. a) ^{1}H NMR spectroscopy titration of **1** (c = 8.89×10^{-4} mol dm^{-3}, V_0 = 450 μL) with TEACl (c = 9.60×10^{-3} mol dm^{-3} up to n(TEACl)/n(**1**) = 1.82; rest with c = 8.065×10^{-2} mol dm^{-3}) in CD_3CN at 25 °C. b), c) Experimental (■), and calculated (—) chemical shifts for selected nuclei at **1**.

Table S6. Calculated ^{1}H NMR chemical shifts (in ppm) for **1** and its complex with Cl^- in CD_3CN at 25 °C. Assignment of protons is depicted in Figure S5. Left/right assignments (= downfield/upfield, respectively) refer to the position of signal in NMR spectrum when assigning pairs of similar protons.

H	**1**	**1**Cl^-
NH-b	8.5742	9.4062
NH-a	7.0202	7.344
e	6.95	6.9111
jk-right	6.8818	6.7728
g	3.7874	3.8421
o-left	3.4354	3.3417
hi-right	3.2886	3.2433
o-right	3.0664	3.3609
p-left	1.1406	1.084
lm-right	1.0194	0.9805
p-right	0.9507	1.0609

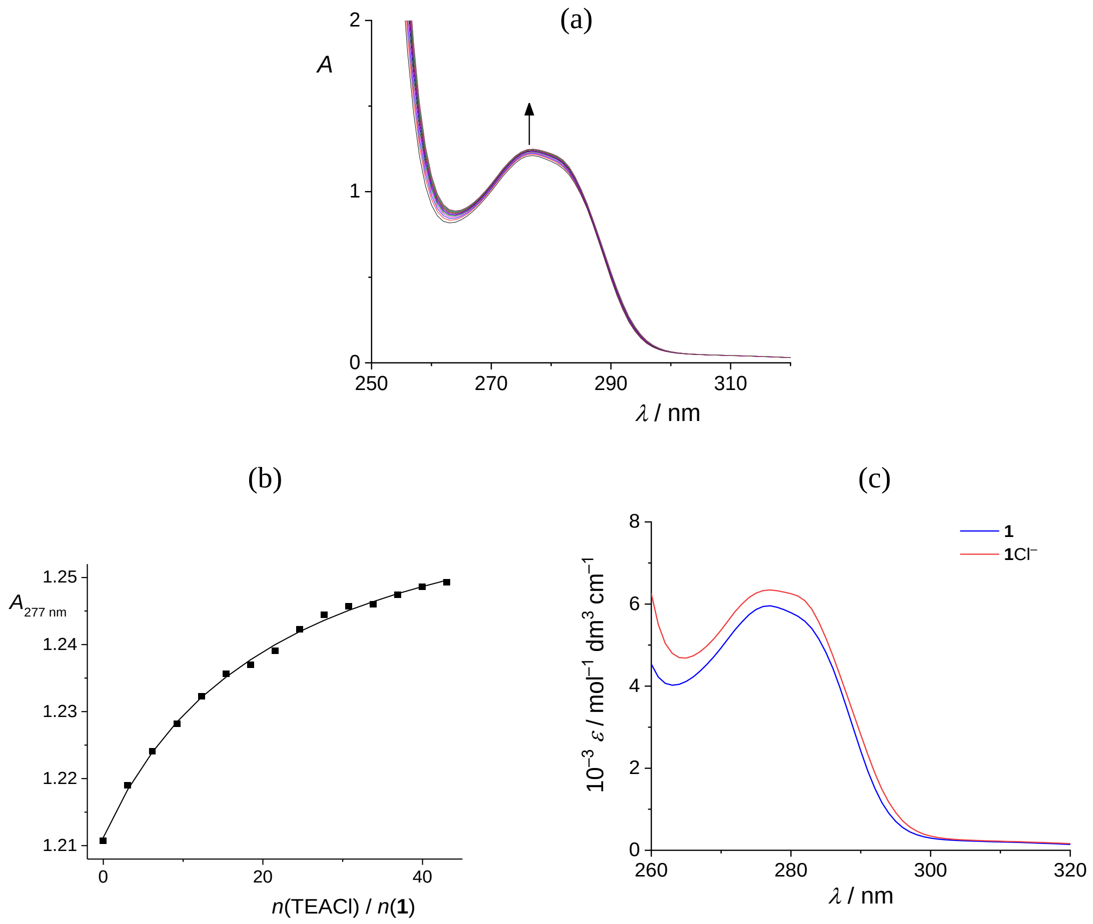

Figure S11. a) Spectrophotometric titration of **1** ($c = 2.03 \times 10^{-4}$ mol dm^{-3}, $V_0 = 2.2$ mL) with TEACl ($c = 1.96 \times 10^{-2}$ mol dm^{-3}) in acetonitrile. $l = 1$ cm; $\vartheta = (25.0 \pm 0.1)$ °C. The spectra are corrected for dilution. b) Dependence of absorbance at 277 nm on n(TEACl) / n(**1**) ratio. ■ experimental; — calculated. c) Characteristic UV/Vis spectra of **1** and its chloride complex.

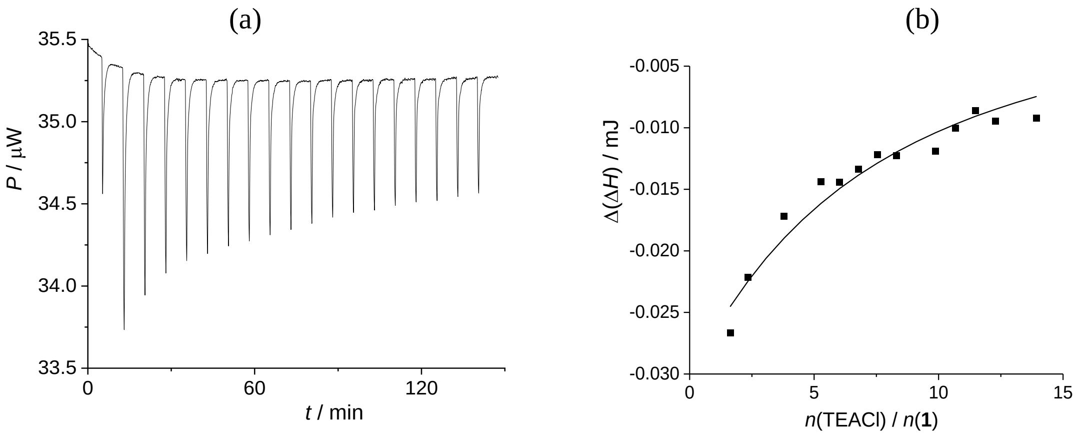

Figure S12. a) Microcalorimetric titration of **1** ($c = 1.53 \times 10^{-4}$ mol dm^{-3}, $V_0 = 1.425$ mL) with TEACl ($c = 1.01 \times 10^{-2}$ mol dm^{-3}) in acetonitrile at 25 °C; b) Dependence of successive enthalpy change on n(TEACl) / n(**1**) ratio. ■ experimental; — calculated.

*Complexation of chloride with **2***

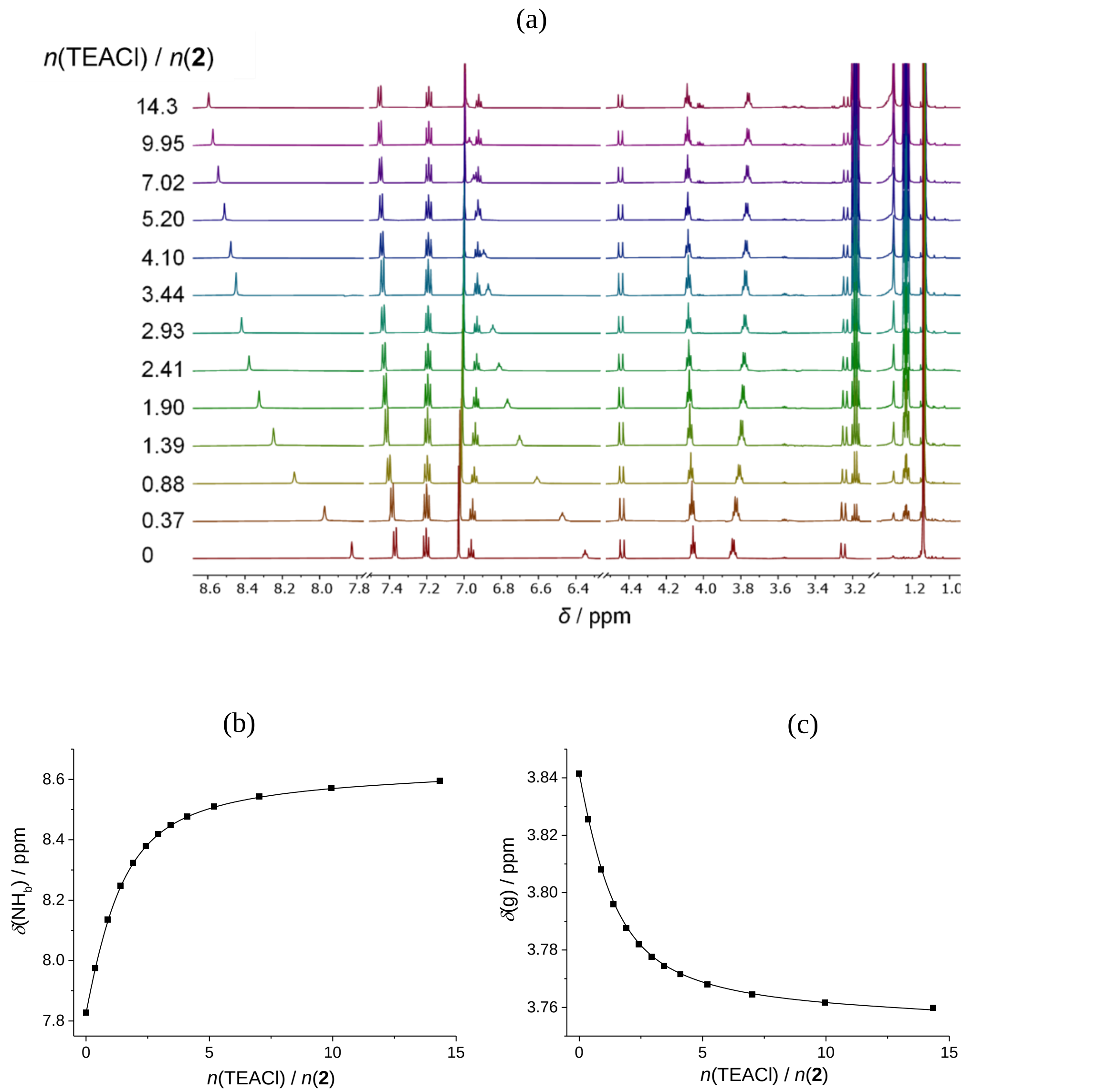

Figure S13. a) ^{1}H NMR spectroscopy titration of **2** ($c = 8.09\times10^{-5}$ mol dm^{-3}, $V_0 = 500$ μL) with TEACl ($c = 2.96\times10^{-3}$ mol dm^{-3}) in CD_3CN at 25 °C. b), c) Experimental (■), and calculated (—) chemical shifts for selected nuclei at **2**.

Table S7. Calculated ^{1}H NMR chemical shifts (in ppm) for **2** and **2**Cl^- in CD_3CN at 25 °C. Assignment of protons is depicted in Figure S5.

H	**2**	**2**Cl^-
a	6.3497	7.0461
b	7.8255	8.6597
c	7.3707	7.4611
d	7.2036	7.1874
e	6.962	6.9173
f	4.0566	4.0908
g	3.8414	3.752
h	3.2515	3.2345
i	4.4363	4.4476
j	7.0297	6.9908
k	1.1424	1.1286

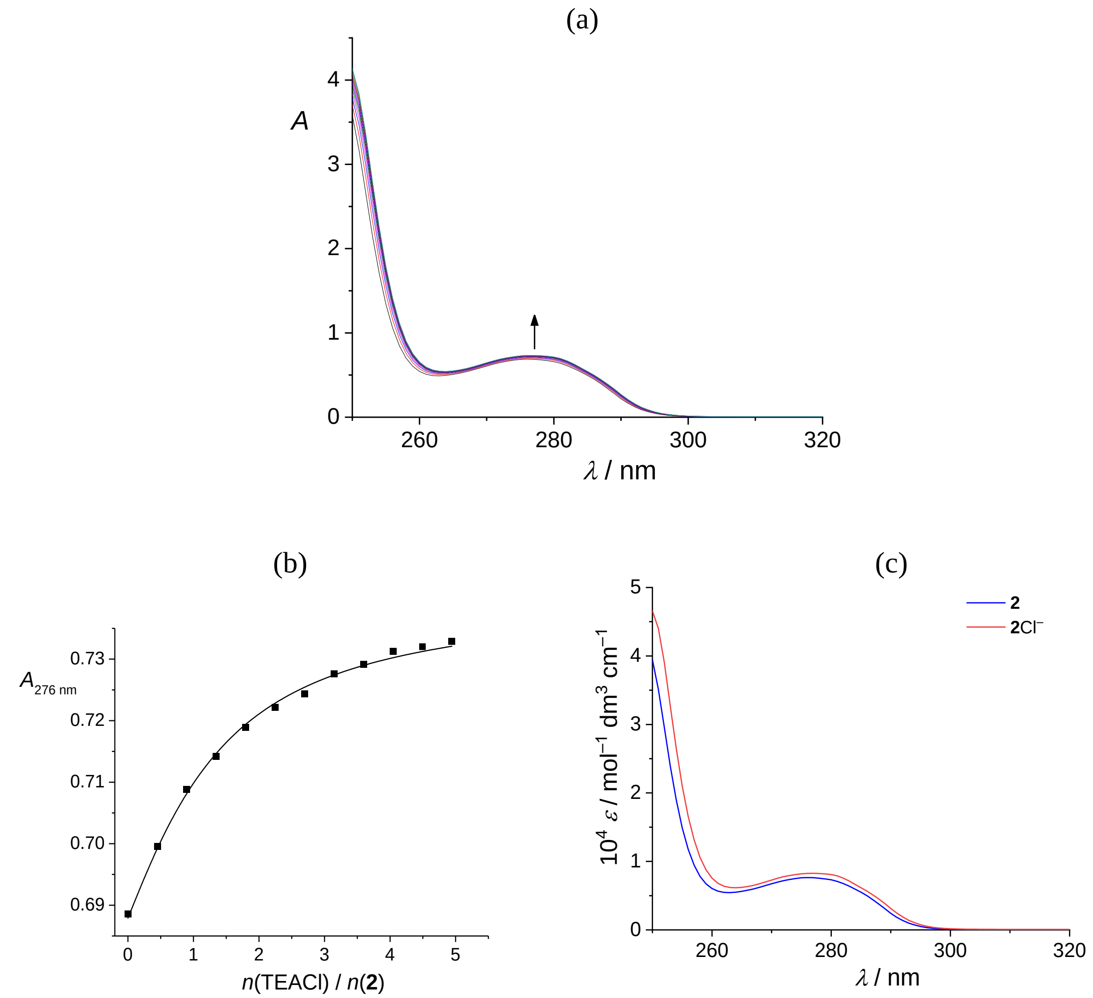

Figure S14. a) Spectrophotometric titration of **2** (c = 9.00×10^{-5} mol dm^{-3}, V_0 = 2.2 mL) with TEACl (c = 3.56×10^{-3} mol dm^{-3}) in acetonitrile. l = 1 cm; ϑ = (25.0 ± 0.1) °C. The spectra are corrected for dilution. b) Dependence of absorbance at 276 nm on n(TEACl) / n(**2**) ratio. ■ experimental; — calculated, c) Characteristic UV/Vis spectra of **2** and its chloride complex.

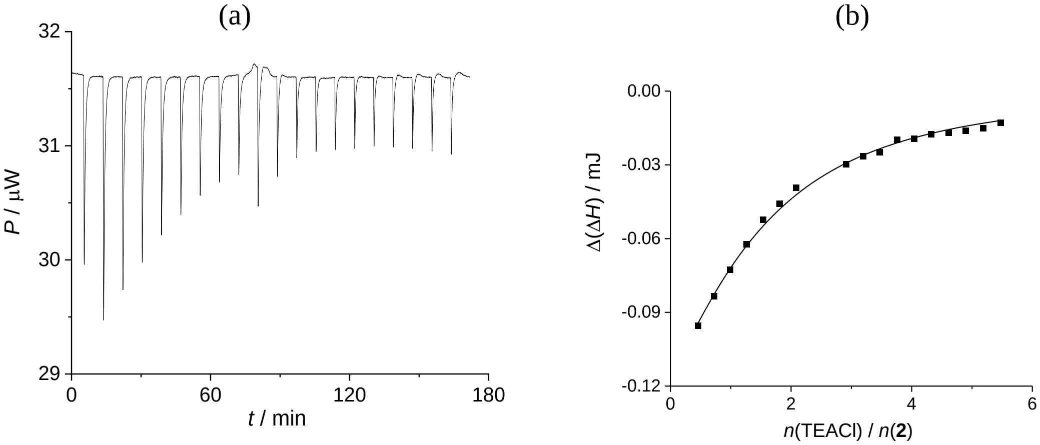

Figure S15. a) Microcalorimetric titration of **2** (c = 6.65×10^{-5} mol dm^{-3}, V_0 = 1.425 mL) with TEACl (c = 3.56×10^{-3} mol dm^{-3}) in acetonitrile at 25 °C; b) Dependence of successive enthalpy change on n(TEACl) / n(**2**) ratio. ■ experimental; — calculated.

*Complexation of chloride with **3***

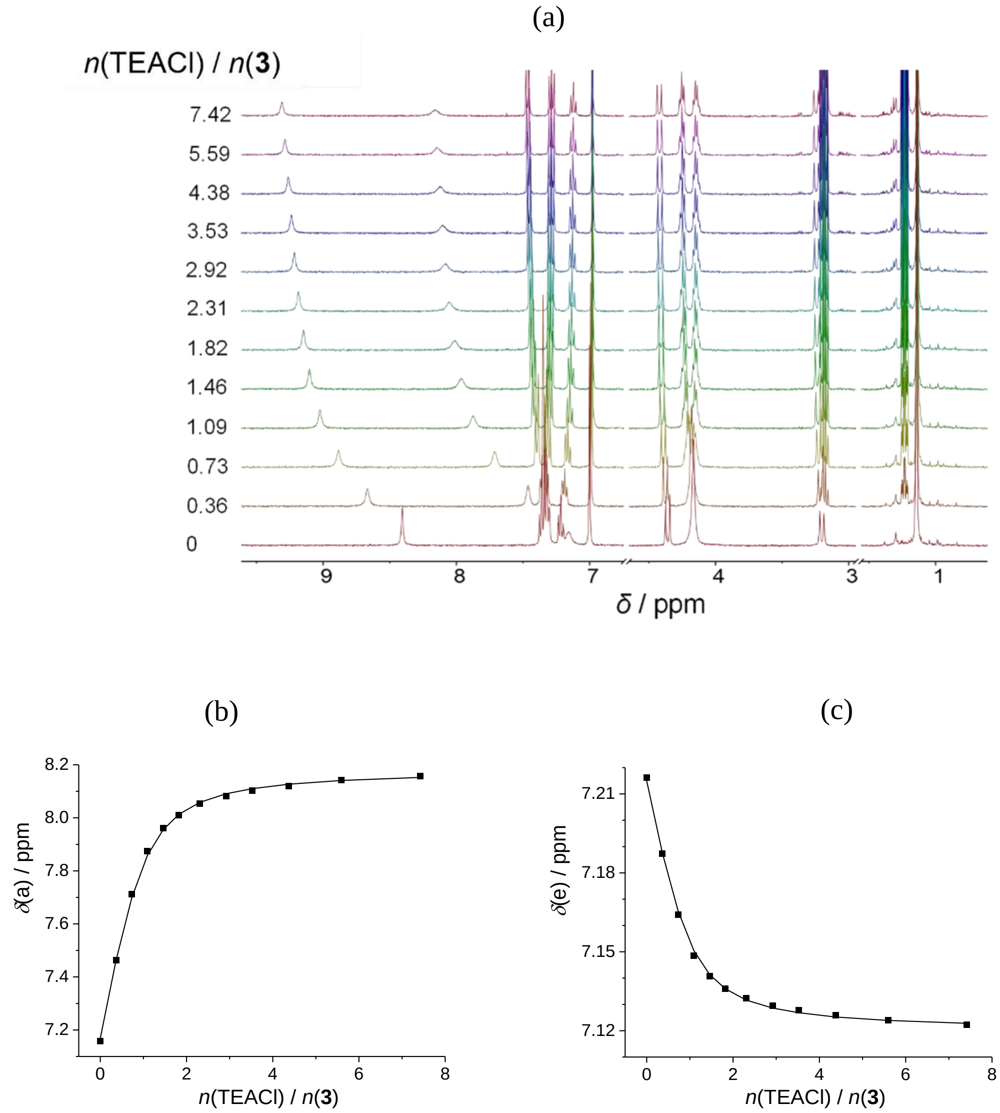

Figure S16. a) ^{1}H NMR spectroscopy titration of **3** (c = 6.02 ×10^{-4} mol dm^{-3}, V_0 = 500 μL) with TEACl (c = 7.32×10^{-3} mol dm^{-3}) in CD_3CN at 25 °C. b), c) Experimental (■), and calculated (—) chemical shifts for selected nuclei at **3**.

Table S8. Calculated ^{1}H NMR chemical shifts (in ppm) for **3** and **3**Cl^- in CD_3CN at 25 °C. Assignment of protons is depicted in Figure S1. Left/right assignments (= downfield/upfield, respectively) refer to the position of signal in NMR spectrum when assigning pair of similar protons.

H	**3**	**3**Cl^-
b	8.405	9.3255
c	7.3215	7.4789
d	7.3552	7.2829
e	7.215	7.1184
a	7.168	8.1997
hi-left	4.3735	4.4383
f	4.1652	4.2569
hi-right	3.2169	3.2625

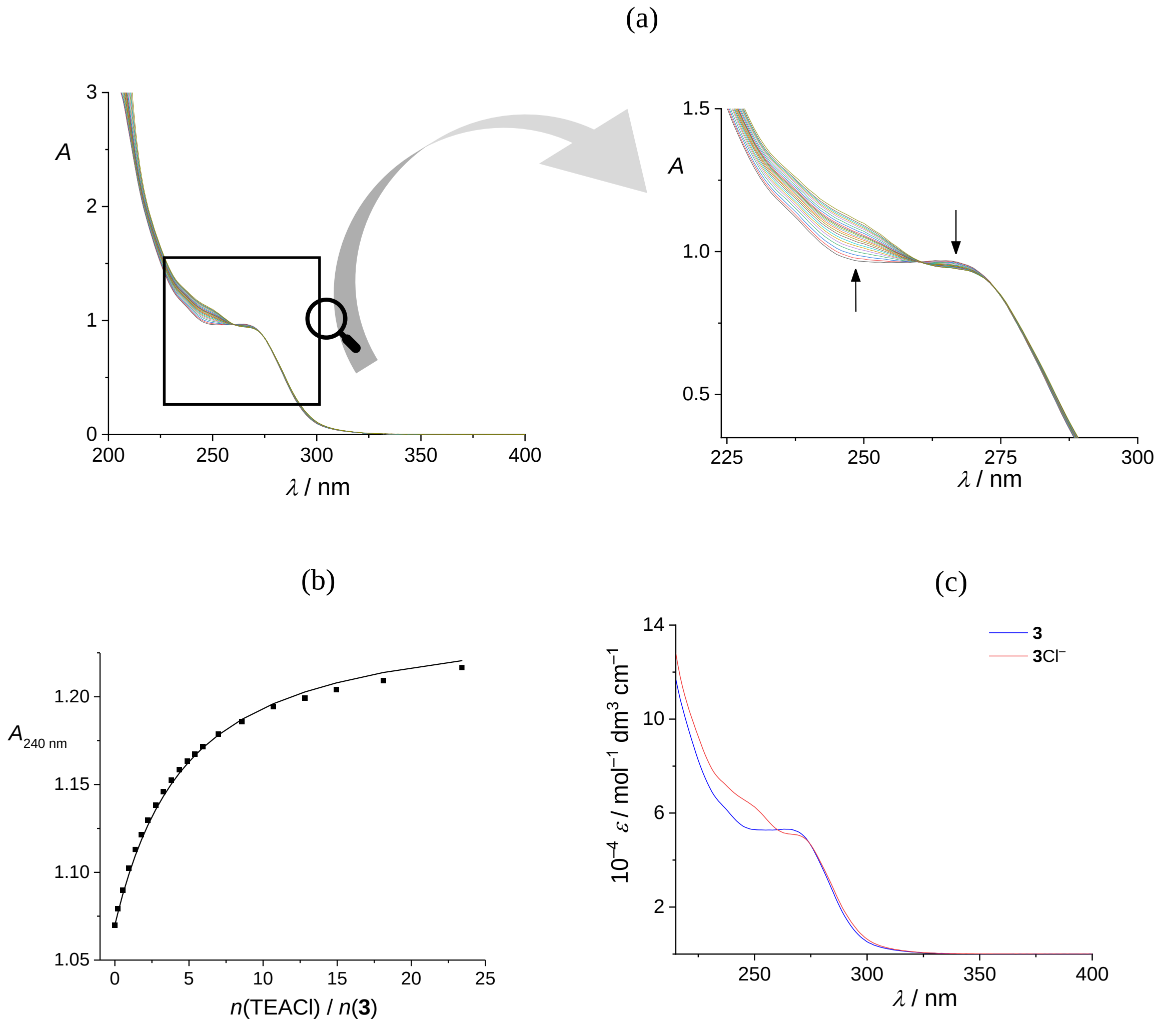

Figure S17. a) Spectrophotometric titration of **3** (c = 1.82×10^{-5} mol dm^{-3}, V_0 = 2.3 mL) with TEACl (c = 8.88×10^{-4} mol dm^{-3}) in acetonitrile. l = 1 cm; ϑ = (25.0 ± 0.1) °C. The spectra are corrected for dilution. b) Dependence of absorbance at 240 nm on n(TEACl) / n(**3**) ratio. ■ experimental; — calculated, c) Characteristic UV/Vis spectra of **3** and its chloride complex.

(a) (b)

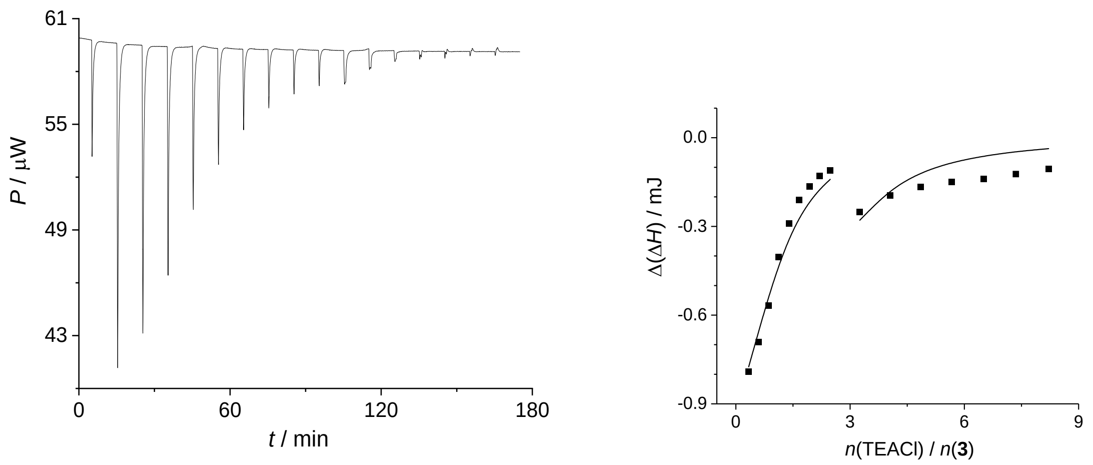

Figure S18. a) Microcalorimetric titration of **3** (c = 4.10×10^{-4} mol dm^{-3}, V_0 = 1.43 mL) with TEACl (c = 1.51×10^{-2} mol dm^{-3}) in acetonitrile at 25 °C. b) Dependence of successive enthalpy change on n(TEACl) / n(**3**) ratio. ■ experimental; — calculated. The discontinuity in the calculated titration curve is a result of the increased volume of titrant addition at $n(Cl^-)/n$(**3**) > 3. This was done with the intention of improving the precision of measuring small thermal effects that occur near the saturation of **3** with chloride.

Hydrogen sulfate

*Complexation of hydrogen sulfate with **1***

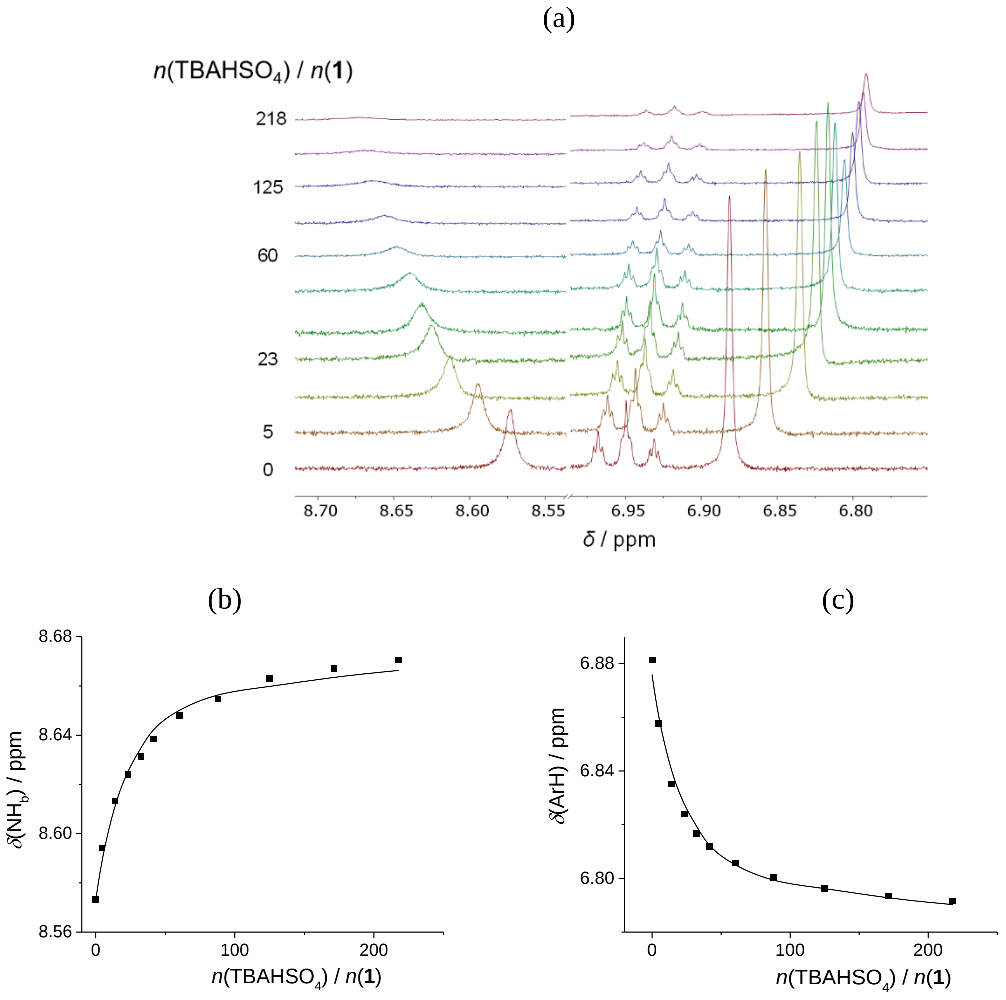

Figure S19. a) ^{1}H NMR spectroscopy titration of **1** (c = 7.93×10^{-4} mol dm^{-3}, V_0 = 500 µL) with $TBAHSO_4$ (c = 0.368 mol dm^{-3}) in CD_3CN at 25 °C. b), c) Experimental (■), and calculated (—) chemical shifts for selected nuclei at **1**.

Table S9. Calculated ^{1}H NMR chemical shifts (in ppm) of **1** and its complex with HSO_4^- in CD_3CN at 25 °C. Assignment of protons is depicted in Figure S5.

H	**1**	**1**HSO_4^-
c	8.5726	8.6834
e	6.876	6.7746
jk	6.9501	6.9147

(a)

(b)

(c)

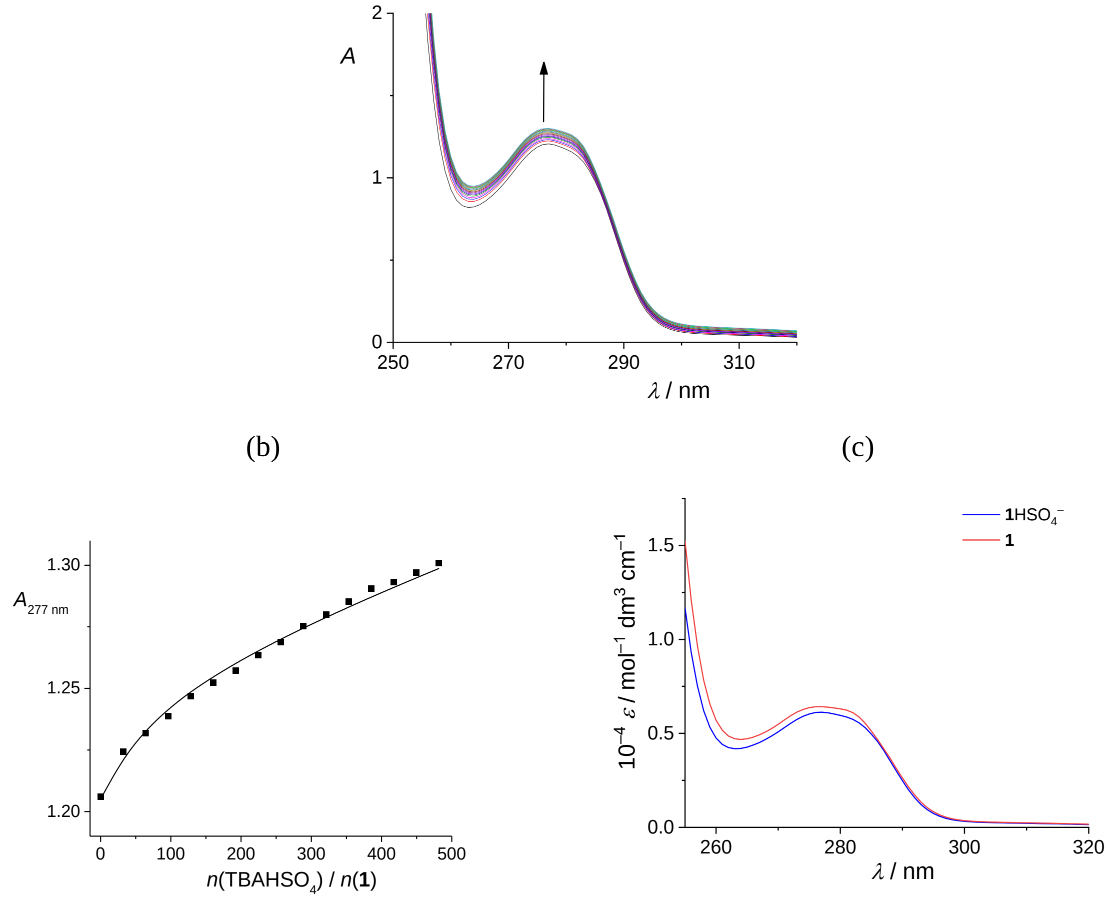

Figure S20. a) Spectrophotometric titration of **1** (c = 1.97×10^{-4} mol dm^{-3}, V_0 = 2.2 mL) with TBAHSO$_4$ (c = 1.98×10^{-1} mol dm^{-3}) in acetonitrile. l = 1 cm; ϑ = (25.0 ± 0.1) °C. The spectra are corrected for dilution. b) Dependence of absorbance at 277 nm on n(TBAHSO$_4$) / n(**1**) ratio. ■ experimental; — calculated. c) Characteristic UV/Vis spectra of **1** and its hydrogensulphate complex.

*Complexation of hydrogen sulfate with **2***

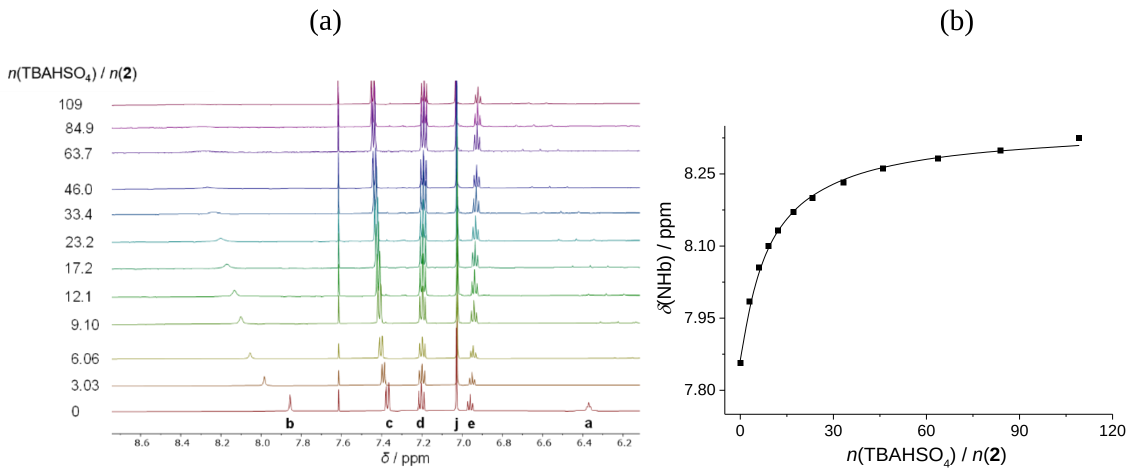

Figure S21. a) ^{1}H NMR spectroscopy titration of **2** (c = 8.09×10^{-5} mol dm^{-3}, V_0 = 500 μL) with TBAHSO$_4$ (c = 2.04×10^{-2} mol dm^{-3}) in CD$_3$CN at 25 °C. b) Experimental (■) and calculated (—) NH$_b$ proton chemical shift at **2**.

Table S10. Calculated ^{1}H NMR chemical shifts (in ppm) of **2** and **2**HSO$_4^-$ in CD$_3$CN at 25 °C. Assignment of protons is depicted in Figure S5.

H	**2**	**2**HSO$_4^-$
b	7.8623	8.3652
c	7.3734	7.4537
f	4.0584	4.0998
g	3.8356	3.7544
e	6.9607	6.9192

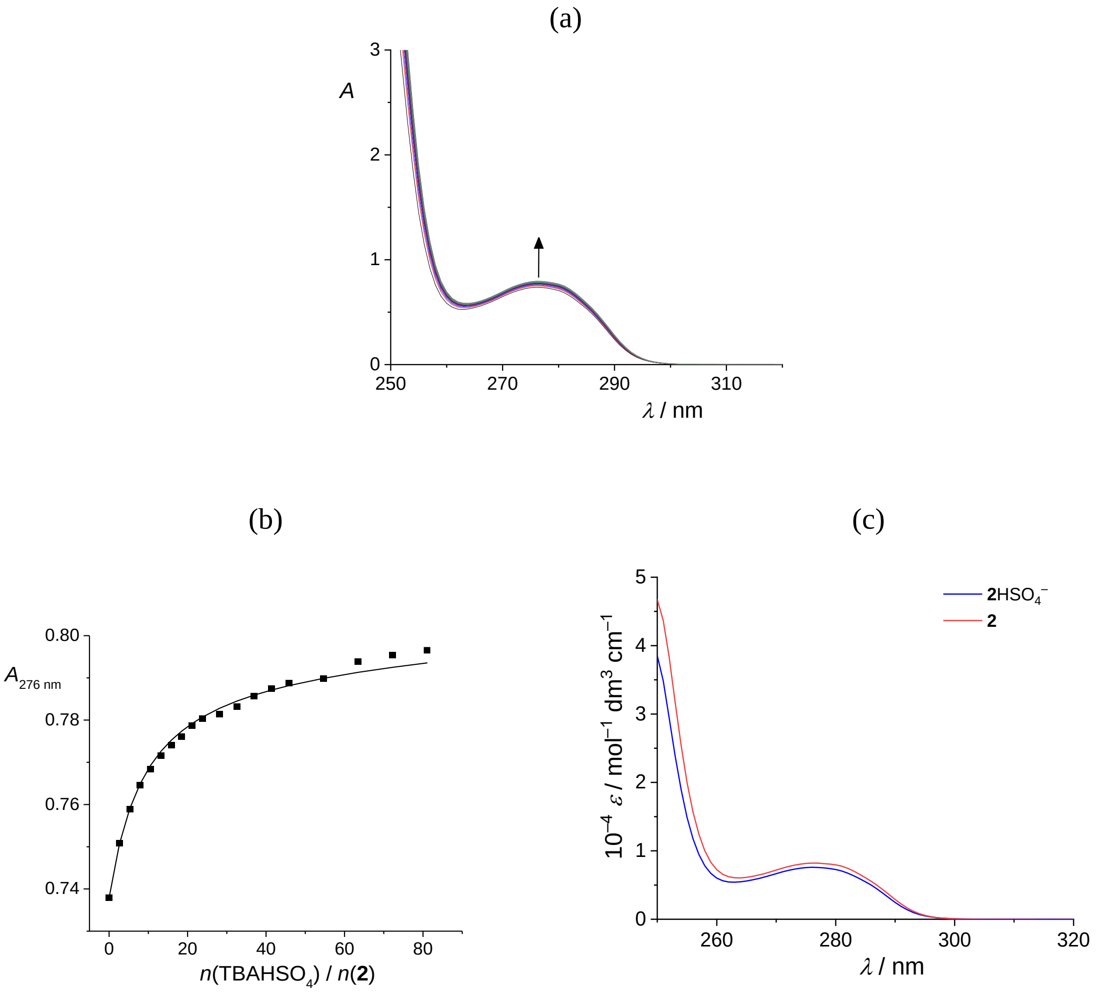

Figure S22. a) Spectrophotometric titration of **2** (c = 9.71×10^{-5} mol dm^{-3}, V_0 = 2.4 mL) with TBAHSO$_4$ (c = 2.05×10^{-2} mol dm^{-3}) in acetonitrile. l = 1 cm; ϑ = (25.0 ± 0.1) °C. The spectra are corrected for dilution. b) Dependence of absorbance at 276 nm on n(TBAHSO$_4$) / n(**2**) ratio. ■ experimental; — calculated (log K(**2**HSO$_4^-$) = 3.06(1)). c) Characteristic UV/Vis spectra of **2** and its hydrogensulphate complex.

*Complexation of hydrogen sulfate with **3***

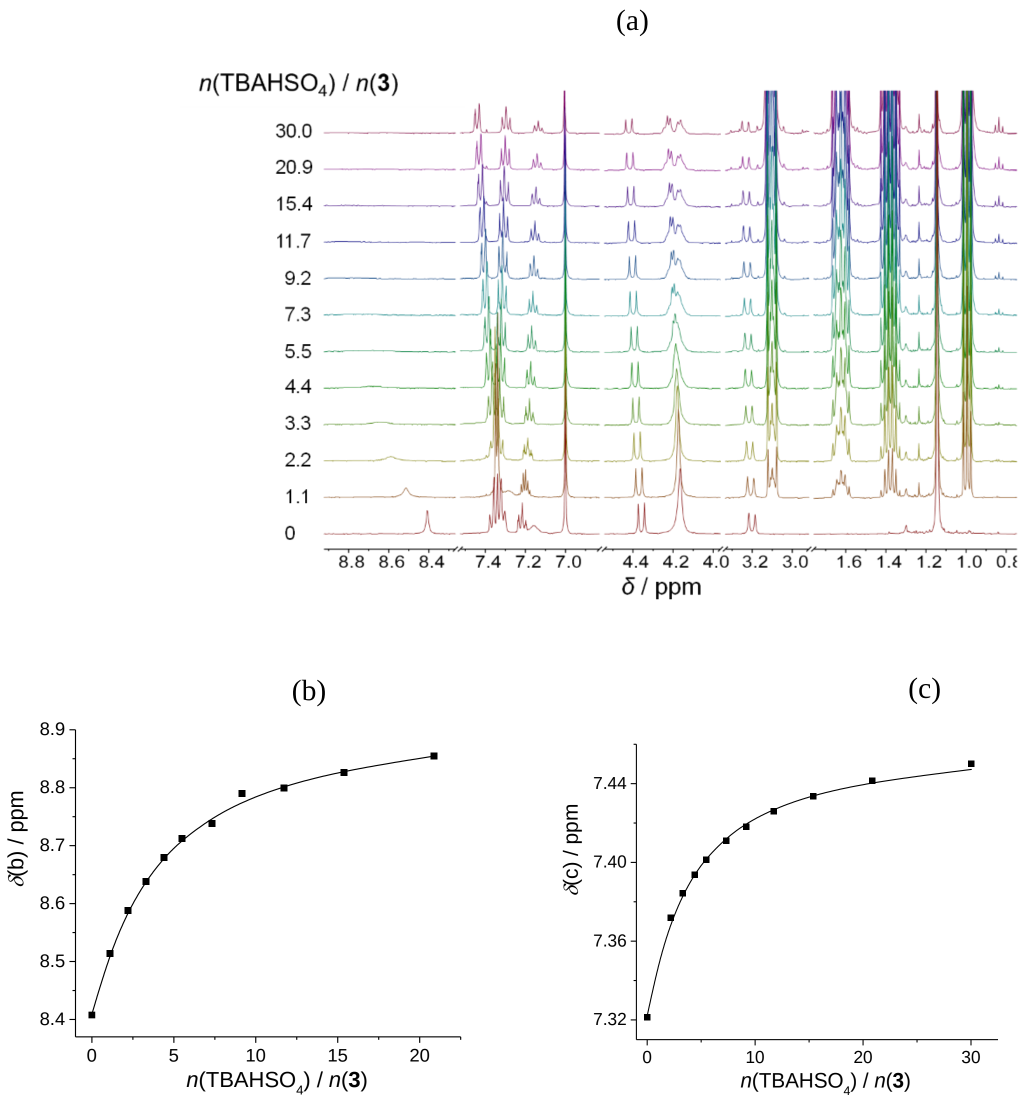

Figure S23. a) ^{1}H NMR spectroscopy titration of **3** (c = 6.02 ×10^{-4} mol dm^{-3}, V_0 = 500 µL) with $TBAHSO_4$ (c = 2.21×10^{-2} mol dm^{-3}) in CD_3CN at 25 °C. b), c Experimental (■), and calculated (—) chemical shifts for selected nuclei at **3**.

Table S11. Calculated ^{1}H NMR chemical shifts (in ppm) for **3** and **3**HSO_4^- in CD_3CN at 25 °C. Assignment of protons is depicted in Figure S1. Left/right assignments (= downfield/upfield, respectively) refer to the position of signal in NMR spectrum when assigning pair of similar protons.

H	**3**	**3**HSO_4^-
b	8.4092	9.0005
c	7.322	7.4799
d	7.3577	7.2841
e	7.2174	7.1188
hi-left	4.3718	4.4491
f	4.1509	4.2468
hi-right	3.2161	3.2575

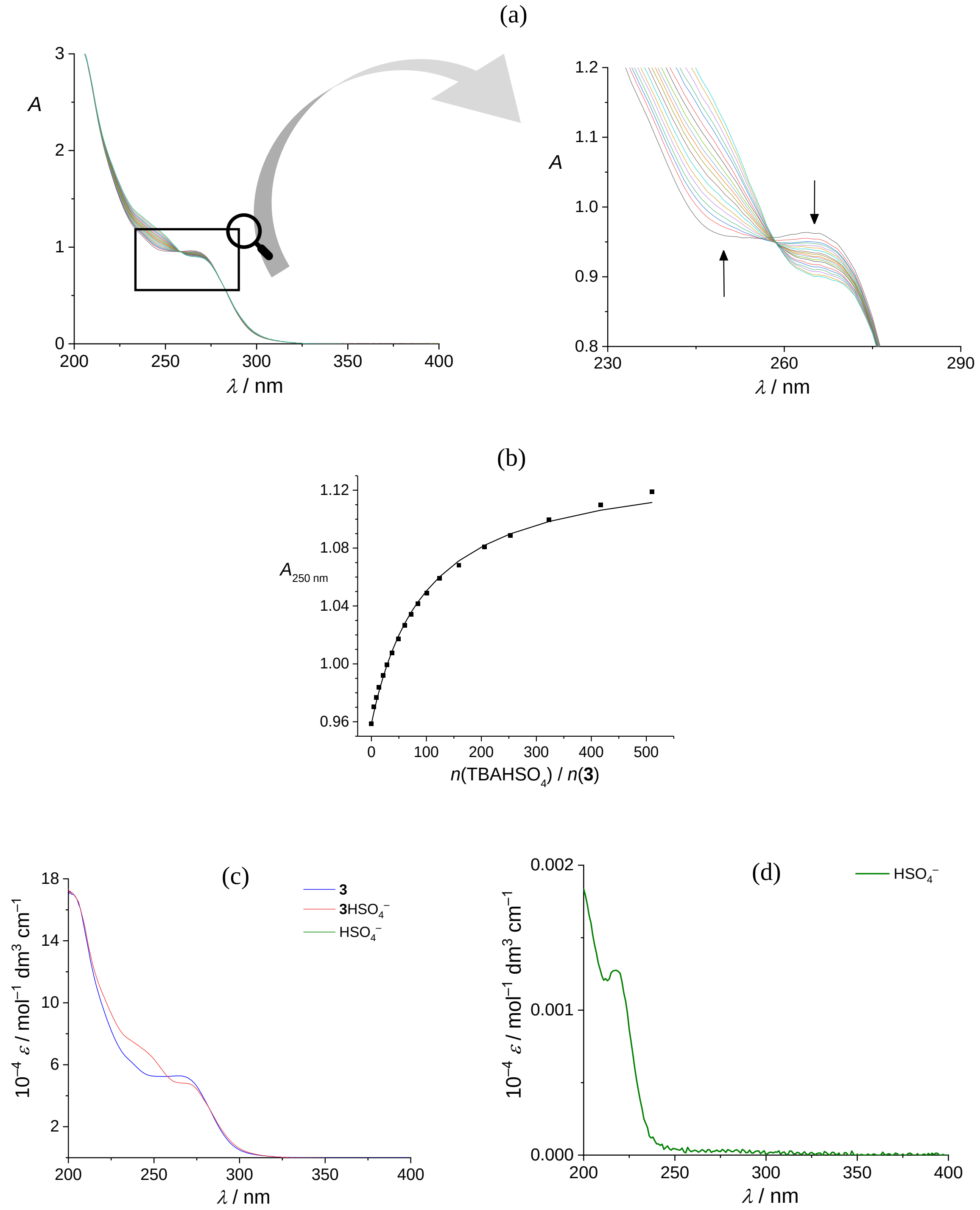

Figure S24. a) Spectrophotometric titration of **3** (c = 1.82×10^{-5} mol dm^{-3}, V_0 = 2.3 mL) with TBAHSO4 (c = 1.96×10^{-2} mol dm^{-3}) in acetonitrile. l = 1 cm; ϑ = (25.0 ± 0.1) °C. The spectra are corrected for the absorption of TBAHSO4 and for the dilution of **3**. b) Dependence of absorbance at 250 nm on n(TBAHSO4) / n(**3**) ratio. ■ experimental; — calculated. Characteristic UV spectra of c) **3** and **3**HSO4^{-}, and d) TBAHSO4.

(a) (b)

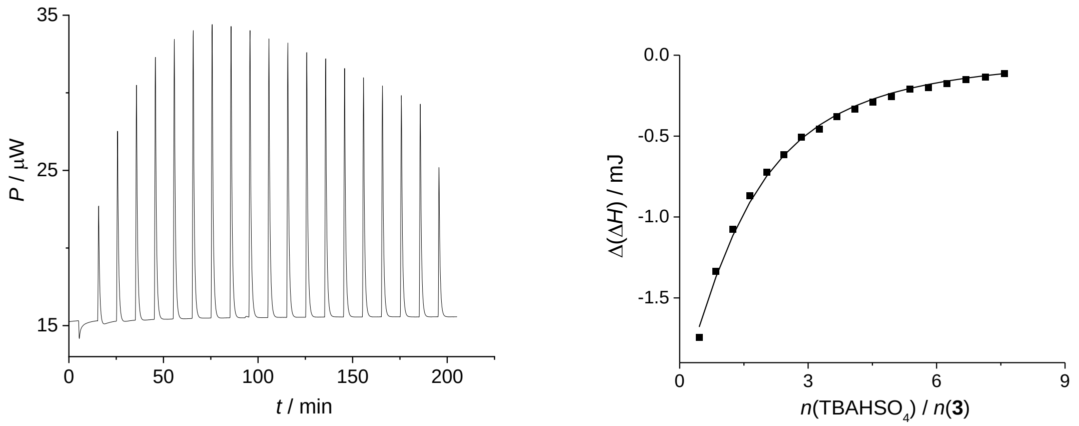

Figure S25. a) Microcalorimetric titration of **3** (c = 8.15×10^{-4} mol dm^{-3}, V_0 = 1.45 mL) with $TBAHSO_4$ (c = 3.01×10^{-2} mol dm^{-3}) in acetonitrile at 25 °C. b) Dependence of successive enthalpy change on n($TBAHSO_4$) / n(**3**) ratio. ■ experimental; — calculated.

Dihydrogen phosphate

*Complexation of dihydrogen phosphate with **1***

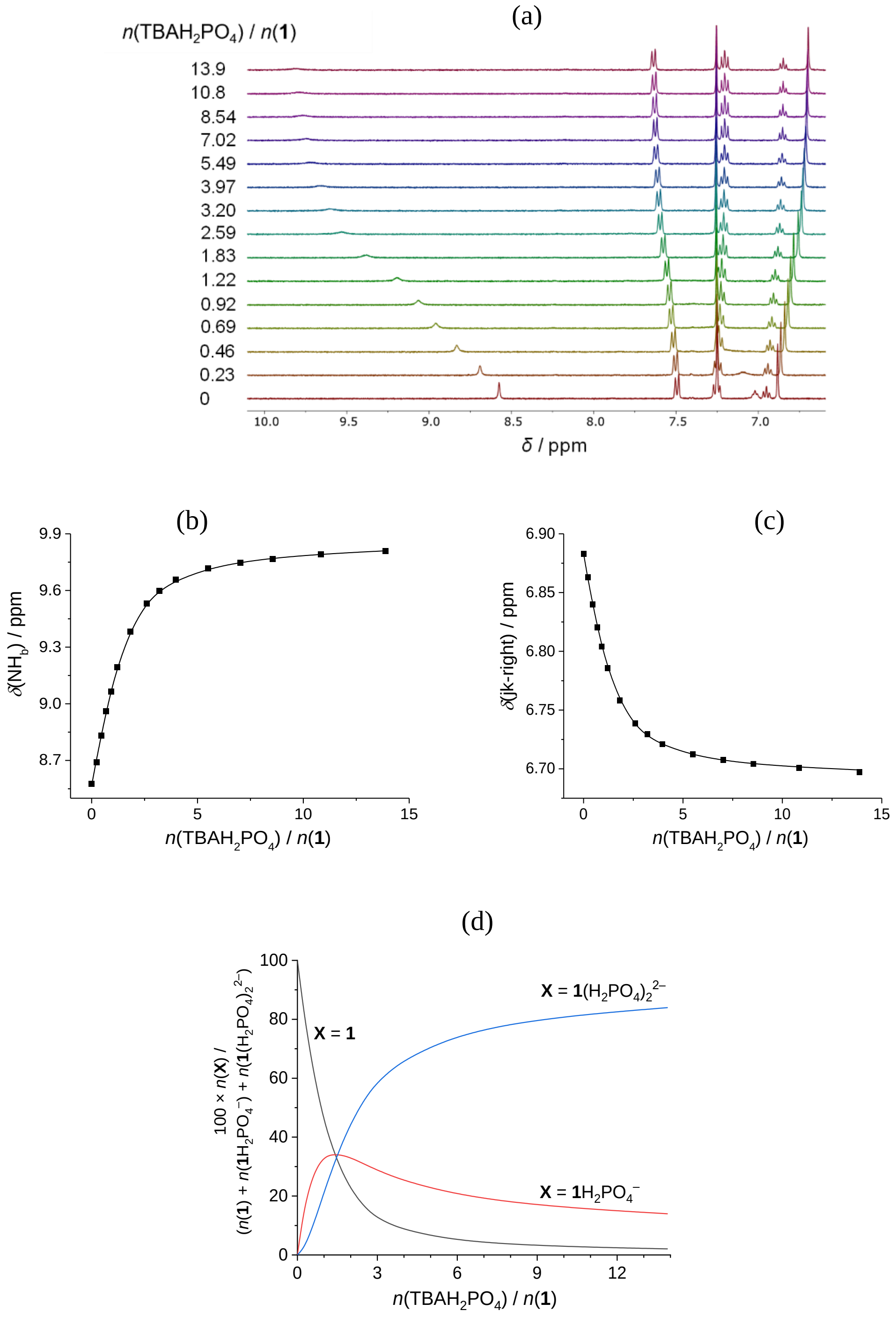

Figure S26. a) ^{1}H NMR spectroscopy titration of **1** (c = 9.67×10^{-4} mol dm^{-3}, V_0 = 500 μL) with $TBAH_2PO_4$ (c = 7.38×10^{-2} mol dm^{-3}) in CD_3CN at 25 °C. b), c) Experimental (■), and calculated (—) chemical shifts for selected nuclei at **1**. d) Distribution of **1** and its complexes with dihydrogen phosphate during the titration.

Table S12. Calculated ^{1}H NMR chemical shifts (in ppm) for **1** and its complexes with $H_2PO_4^-$ in CD_3CN at 25 °C. Assignment of protons is depicted in Figure S5. Left/right assignments (= downfield/upfield, respectively) refer to the position of signal in NMR spectrum when assigning pairs of similar protons.

H	**1**	**1**$H_2PO_4^-$	**1**$(H_2PO_4)_2^{2-}$
NH-b	8.5672	9.3126	9.9235
c	7.4833	7.5346	7.644
e	6.9525	6.8826	6.8398
jk-right	6.8832	6.758	6.6847
o-left	3.4366	3.3814	3.3001
o-right	3.068	3.1692	3.5251
p-right	0.9512	0.9963	1.1312
hi-right	3.2896	3.2483	3.2062
lm-left	1.2931	1.3328	1.3485
lm-right	1.02	0.9659	0.9449

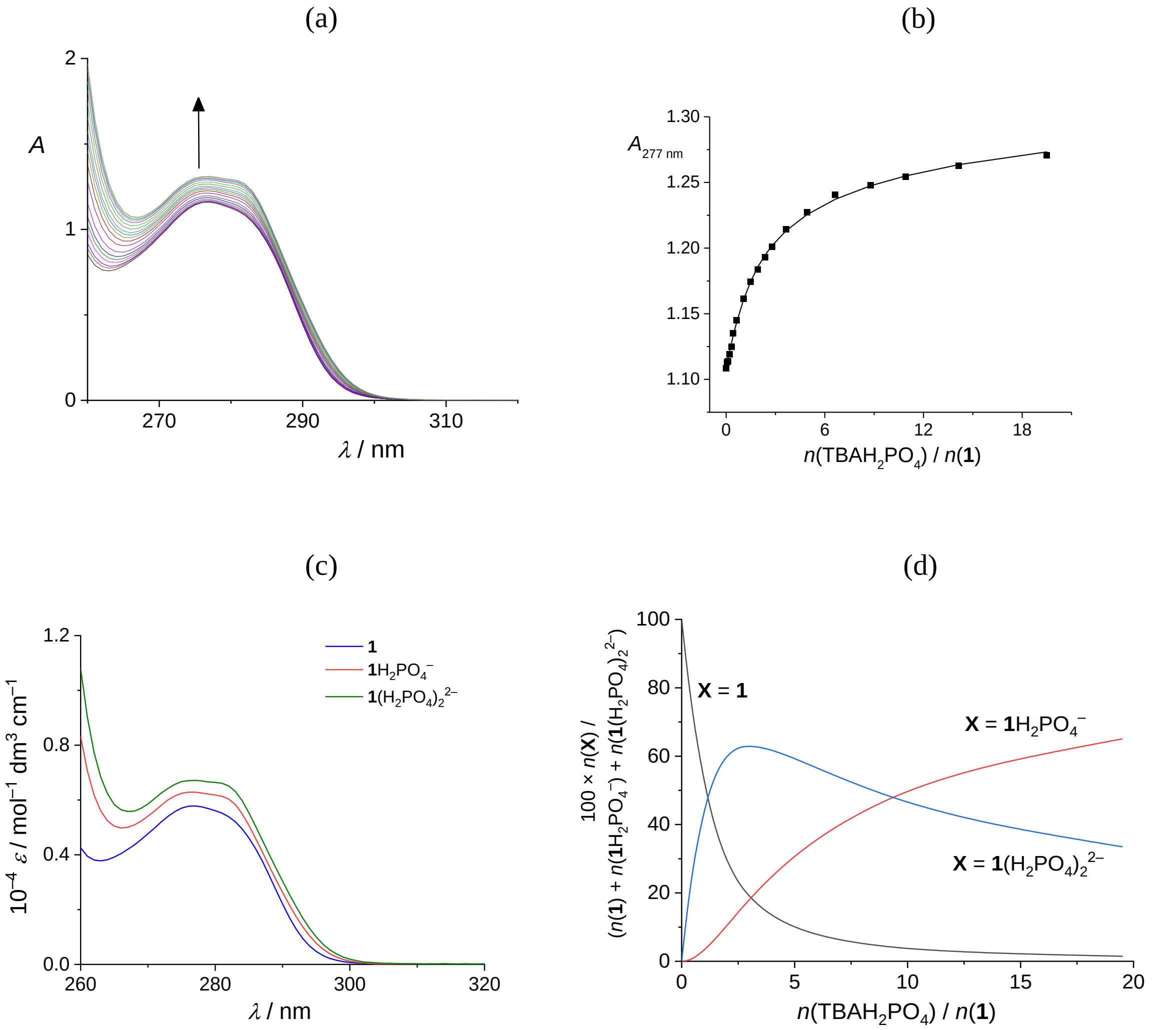

Figure S27. a) Spectrophotometric titration of **1** ($c = 2.00\times10^{-4}$ mol dm^{-3}, $V_0 = 2.3$ mL) with TBAH_2PO_4 ($c = 9.87\times10^{-3}$ mol dm^{-3}) in acetonitrile. $l = 1$ cm; $\vartheta = (25.0 \pm 0.1)$ °C. The spectra are corrected for dilution. b) Dependence of absorbance at 277 nm on n(TBAH_2PO_4) / n(**1**) ratio. ■ experimental; — calculated, c) Characteristic UV/Vis spectra of **1** and its dihydrogen phosphate complexes. d) Distribution of **1** and its complexes with dihydrogen phosphate during the titration of **1** with TBAH_2PO_4.

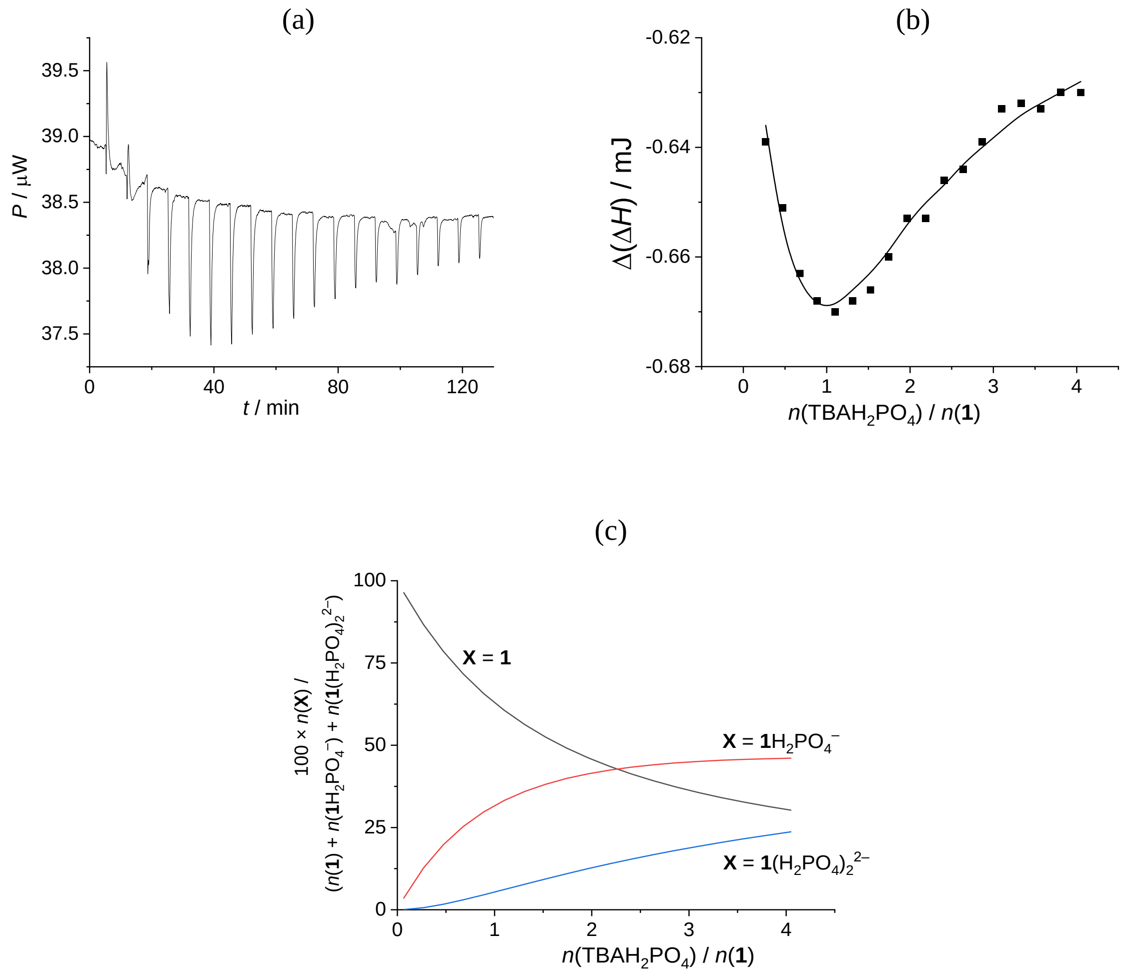

Figure S28. a) Microcalorimetric titration of **1** ($c = 1.99\times10^{-4}$ mol dm^{-3}, $V_0 = 1.432$ mL) with $TBAH_2PO_4$ ($c = 3.78\times10^{-3}$ mol dm^{-3}) in acetonitrile at 25 °C, not corrected for dilution. b) Dependence of successive enthalpy change (corrected for HypDH processing)* on $n(TBAH_2PO_4)$ / n(**1**) ratio. ■ Experimental; — calculated. c) Distribution of **1** and its complexes with dihydrogenphosphate during the titration.

* The correction consisted of the addition of the heat of formation of $(H_2PO_4)_2^{2-}$ present in the volume of the single injection to the measured successive enthalpy changes, according to the procedure described in the work of Horvat et al.[6]

*Complexation of dihydrogen phosphate with **2***

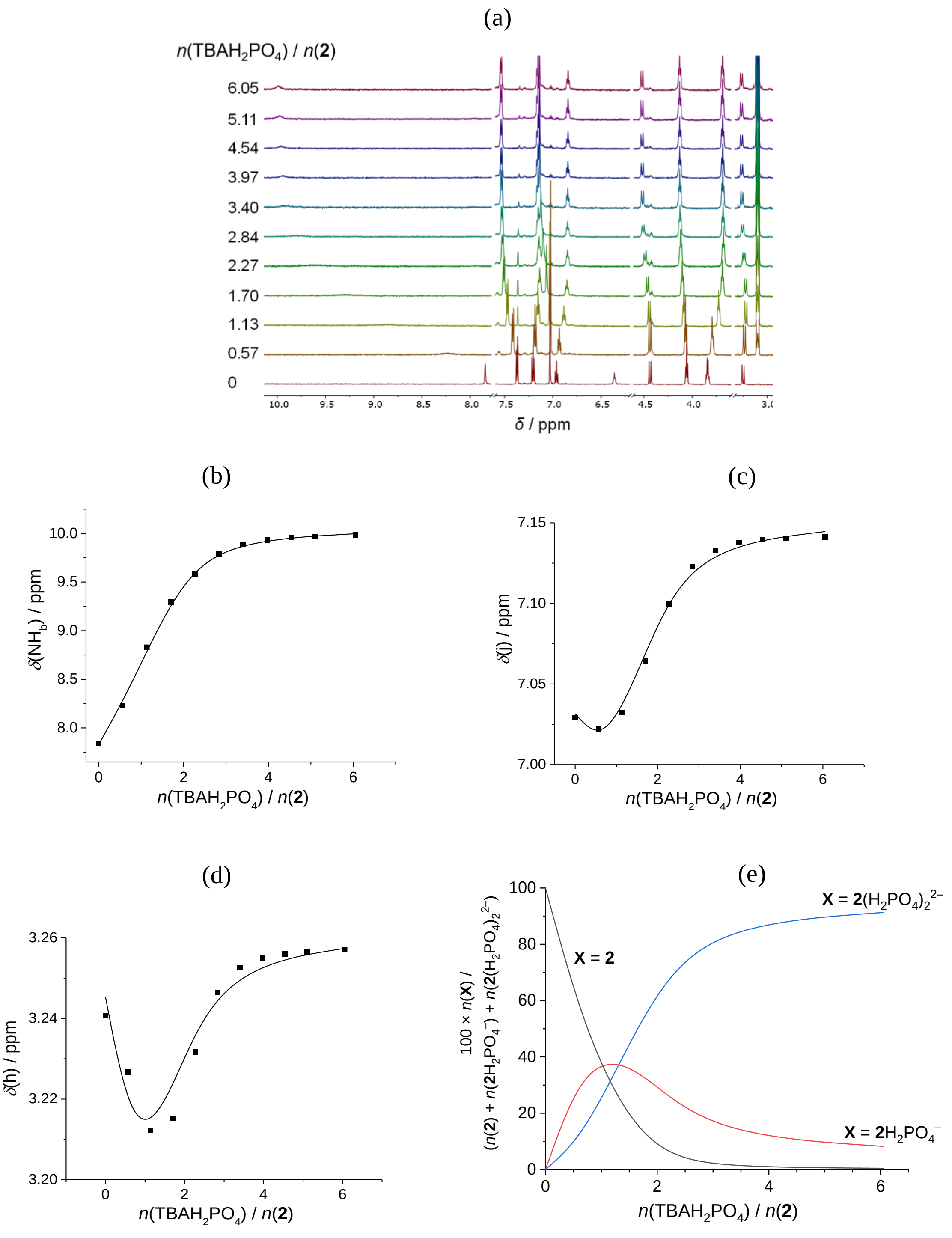

Figure S29. a) ^{1}H NMR spectroscopy titration of **2** (c = 8.09×10^{-5} mol dm^{-3}, V_0 = 500 μL) with $TBAH_2PO_4$ (c = 1.53×10^{-3} mol dm^{-3}) in CD_3CN at 25 °C. b), c), d) Experimental (■), and calculated (—) chemical shifts for selected nuclei at **2**. e) Distribution of **2** and its complexes with dihydrogen phosphate during the titration of **2** with $TBAH_2PO_4$.

Table S13. Calculated ^{1}H NMR chemical shifts (in ppm) of **2**, **2**$H_2PO_4^-$ and **2**$(H_2PO_4)_2^{2-}$ in CD_3CN at 25 °C. Assignment of protons is depicted in Figure S5.

H	**2**	**2**$H_2PO_4^-$	**2**$(H_2PO_4)_2^{2-}$
b	7.8296	8.5045	10.1424
c	7.3663	7.4886	7.5408
d	7.2082	7.1083	7.1559
j	7.0315	6.9389	7.1637
e	6.9659	6.8585	6.8373
i	4.4287	4.3697	4.5253
f	4.0561	4.0698	4.1373
g	3.8474	3.6667	3.6798
h	3.2452	3.147	3.2674
k	1.1428	1.1088	1.1936

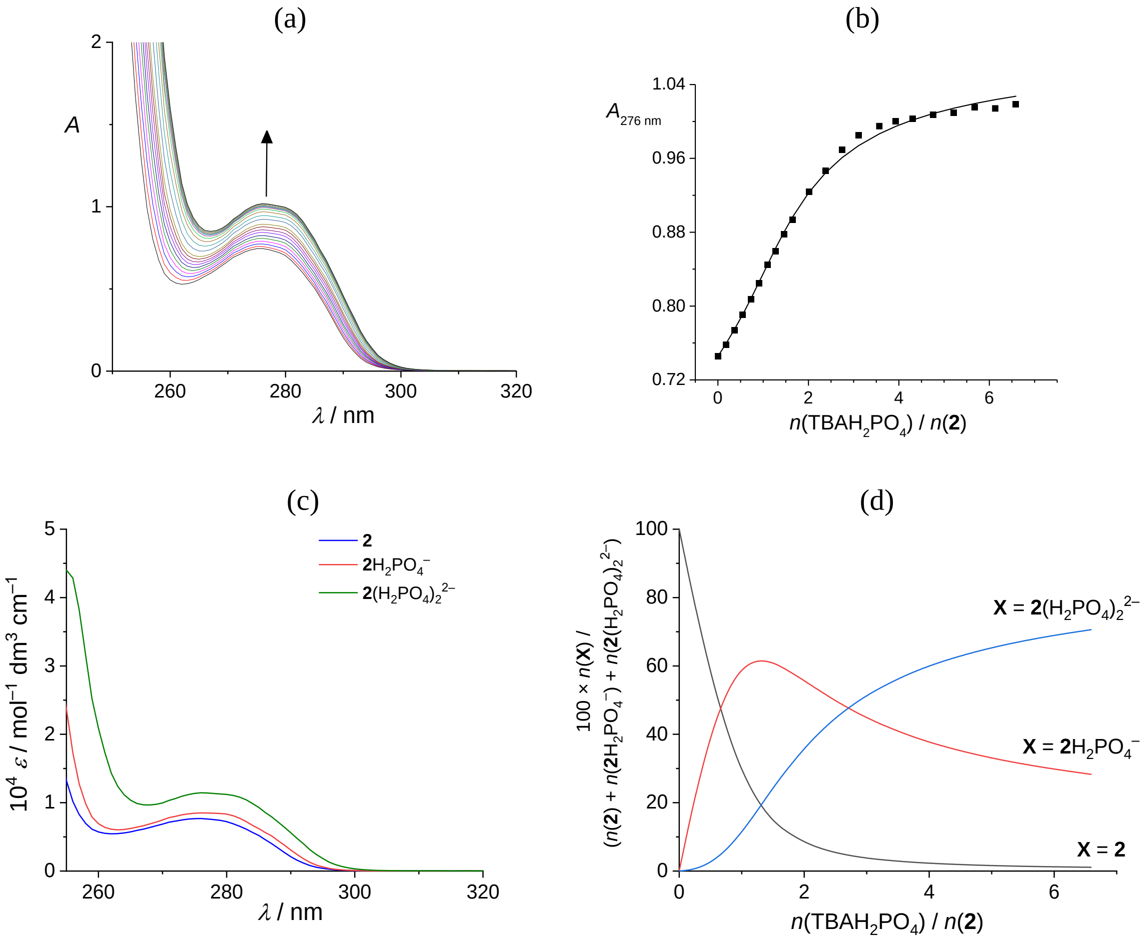

Figure S30. a) Spectrophotometric titration of **2** ($c = 9.71\times10^{-5}$ mol dm^{-3}, $V_0 = 2.2$ mL) with $TBAH_2PO_4$ ($c = 3.91\times10^{-3}$ mol dm^{-3}) in acetonitrile. $l = 1$ cm; $\vartheta = (25.0 \pm 0.1)$ °C. The spectra are corrected for dilution. b) Dependence of absorbance at 276 nm on $n(TBAH_2PO_4)$ / n(**2**) ratio. ■ experimental; — calculated, c) Characteristic UV/Vis spectra of **2** and its dihydrogenphosphate complexes. d) Distribution of **2** and its complexes with dihydrogenphosphate during the titration of **2** with $TBAH_2PO_4$.

(a)

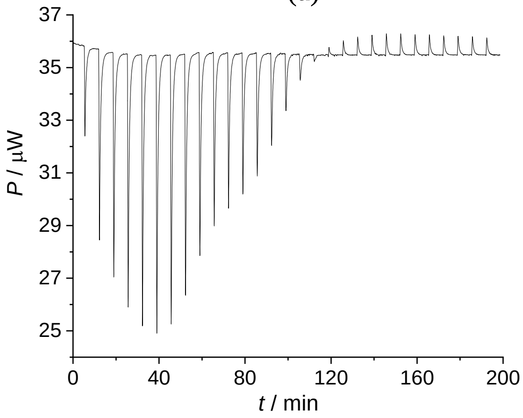

(b)

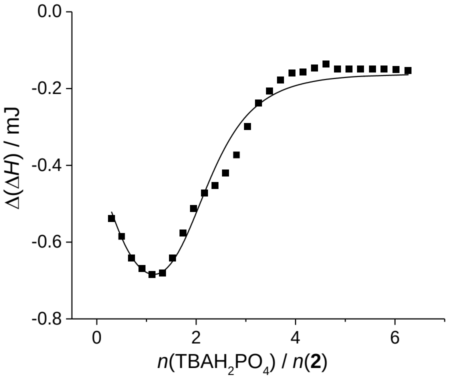

(c)

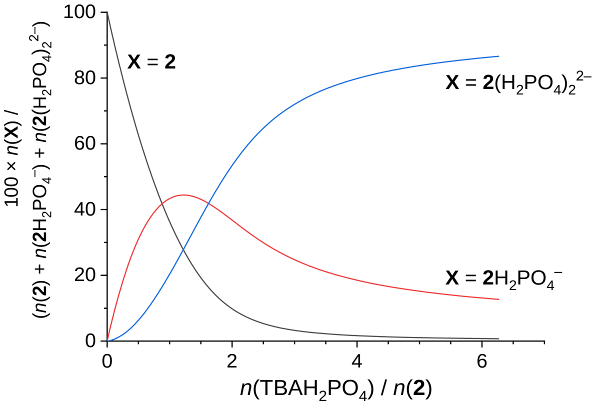

Figure S31. a) Microcalorimetric titration of **2** ($c = 6.60\times10^{-5}$ mol dm^{-3}, $V_0 = 1.425$ mL) with $TBAH_2PO_4$ ($c = 1.86\times10^{-3}$ mol dm^{-3}) in acetonitrile at 25 °C; b) Dependence of successive enthalpy change (corrected for HypDH processing)* on $n(TBAH_2PO_4)$ / n(**2**) ratio. ■ Experimental; — calculated. c) Distribution of **2** and its complexes with dihydrogenphosphate during the titration of **2** with $TBAH_2PO_4$.

* The correction consisted of the addition of the heat of formation of $(H_2PO_4)_2^{2-}$ present in the volume of the single injection to the measured successive enthalpy changes, according to the procedure described in the work of Horvat et al.[6]

*Complexation of dihydrogen phosphate with **3***

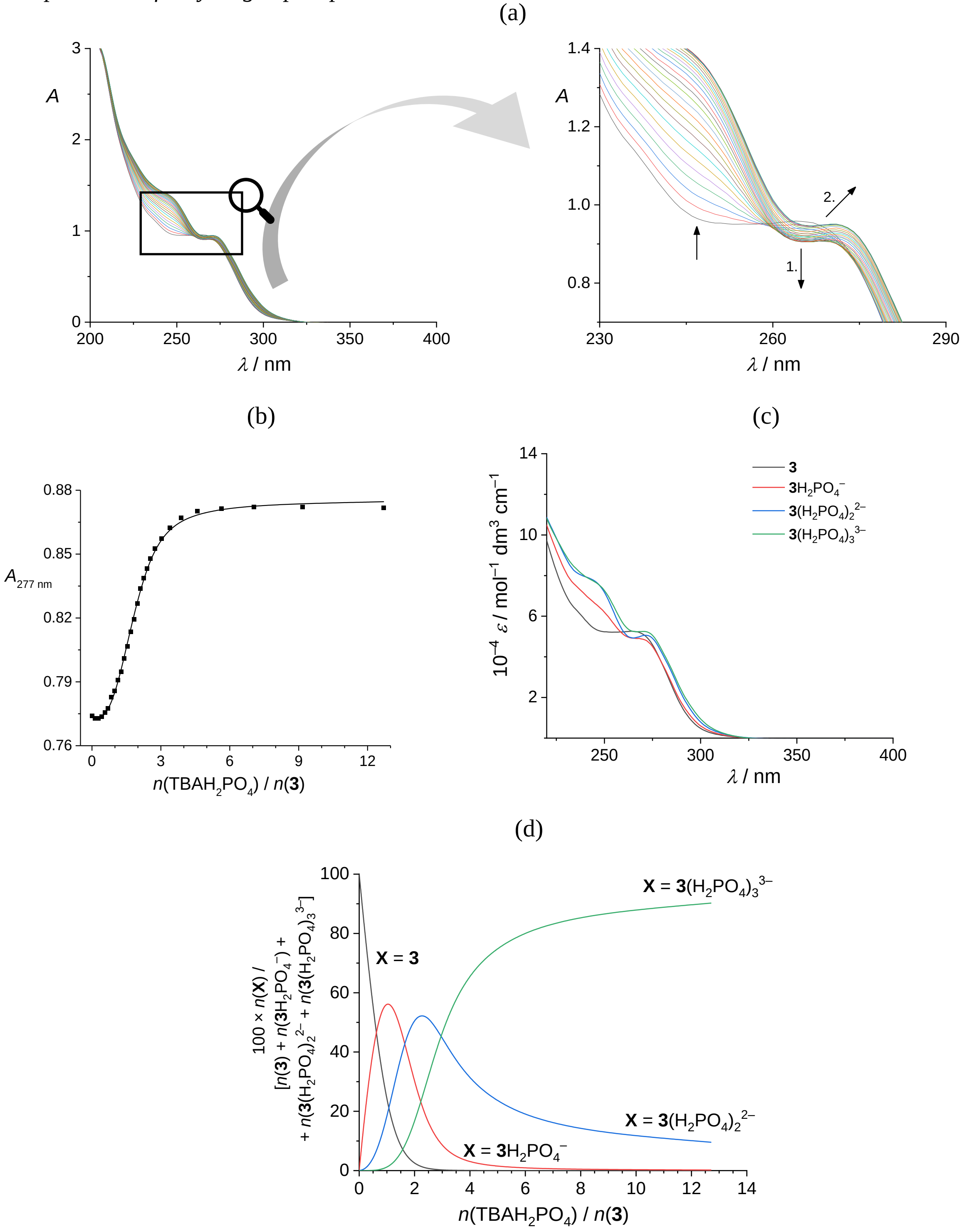

Figure S32. a) Spectrophotometric titration of **3** (c = 1.82×10^{-5} mol dm^{-3}, V_0 = 2.3 mL) with TBAH$_2$PO$_4$ (c = 5.92×10^{-4} mol dm^{-3}) in acetonitrile. l = 1 cm; ϑ = (25.0 ± 0.1) °C. The spectra are corrected for dilution. b) Dependence of absorbance at 277 nm on n(TBAH$_2$PO$_4$) / n(**3**) ratio. ■ experimental; — calculated. c) Characteristic UV/Vis spectra of **3** and its dihydrogenphosphate complexes. d) Distribution of **3** and its complexes with dihydrogenphosphate during the titration of **3** with TBAH$_2$PO$_4$.

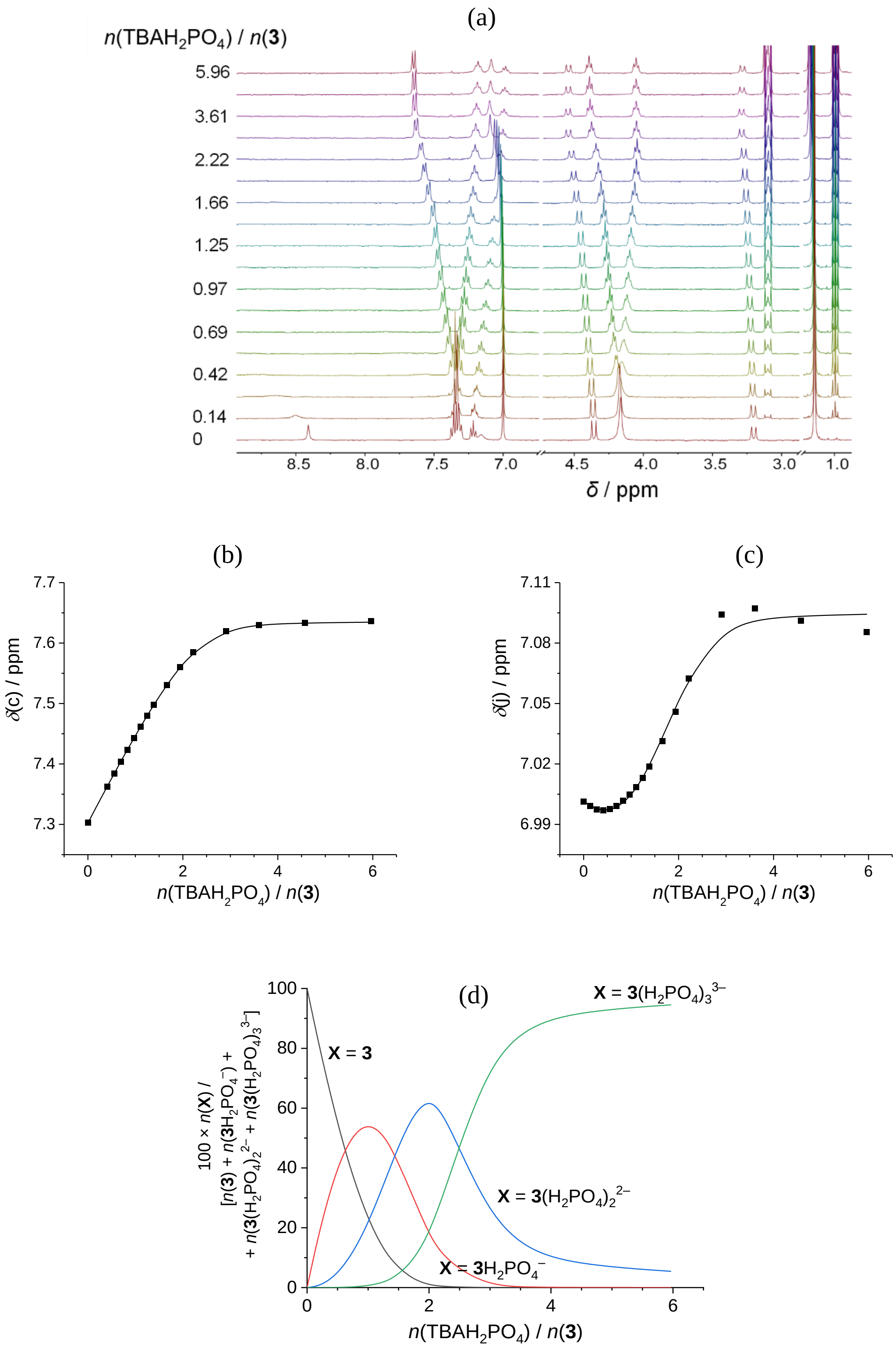

Figure S33. a) ^{1}H NMR spectroscopy titration of **3** (c = 4.77 ×10^{-4} mol dm^{-3}, V_0 = 480 μL) with $TBAH_2PO_4$ (c = 6.35×10^{-3} mol dm^{-3}) in CD_3CN at 25 °C. b), c) Experimental (■), and calculated (—) chemical shifts for selected nuclei at **3**. d) Distribution of **3** and its complexes with dihydrogen phosphate during the titration of **3** with $TBAH_2PO_4$.

Table S14. Calculated ^{1}H NMR chemical shifts (in ppm) for **3** and its complexes with dihydrogenphosphate in CD_3CN at 25 °C. Assignment of protons is depicted in Figure S1. Left/right assignments (= downfield/upfield, respectively) refer to the position of signal in NMR spectrum when assigning pair of similar protons.

H	**3**	**3**$H_2PO_4^-$	**3**$(H_2PO_4)_2^{2-}$	**3**$(H_2PO_4)_3^{3-}$
c	7.3021	7.4524	7.5804	7.6377
d	7.3619	7.254	7.1947	7.1884
e	7.2222	7.0951	7.0087	6.9872
j	7.0015	6.9833	7.0582	7.0964
hi-left	4.3722	4.4445	4.5381	4.562
f	4.1607	4.2681	4.33	4.3954
g	4.1815	4.1052	4.0301	4.0546
hi-right	3.2167	3.2496	3.2883	3.304
k	1.1437	1.142	1.1684	1.1842

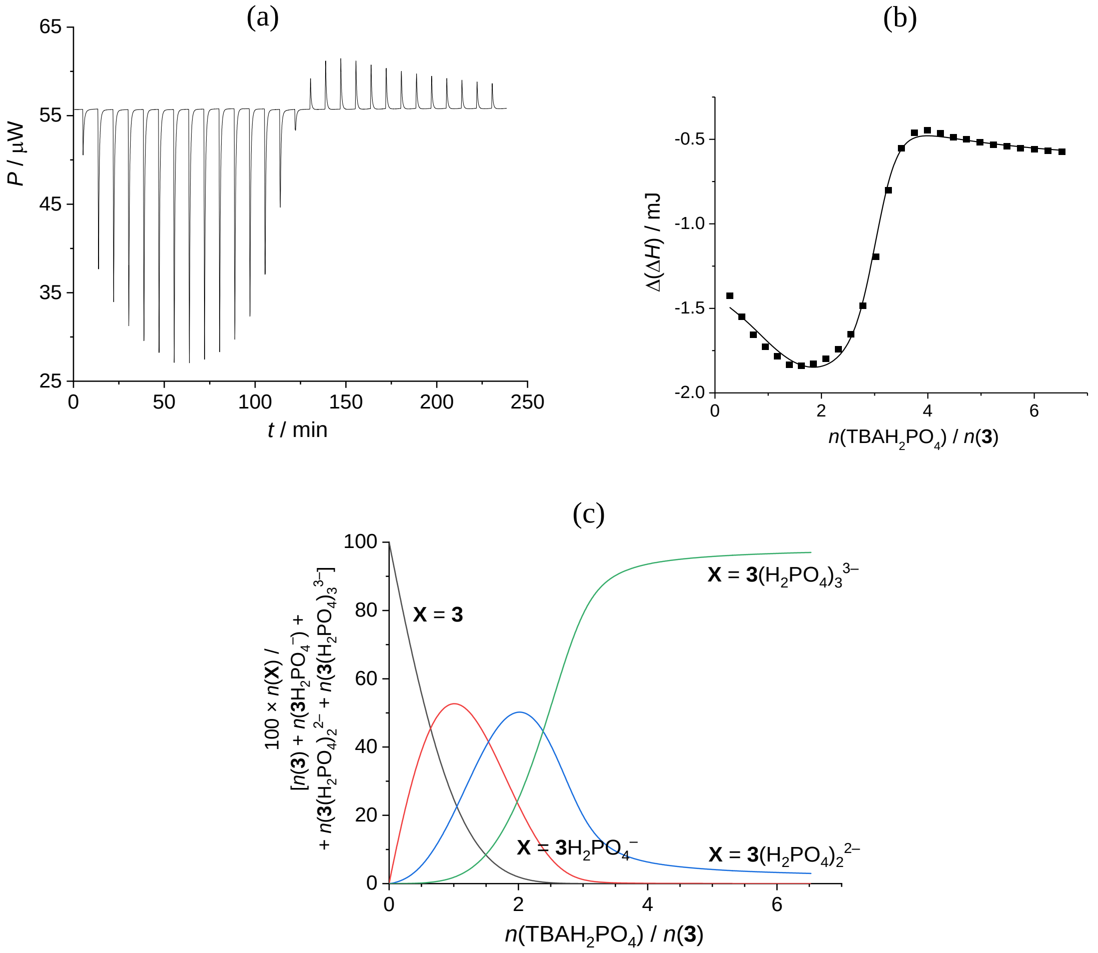

Figure S34. a) Microcalorimetric titration of **3** (c = 1.90×10^{-4} mol dm^{-3}, V_0 = 1.43 mL) with $TBAH_2PO_4$ (c = 5.89×10^{-3} mol dm^{-3}) in acetonitrile at 25 °C, not corrected for dilution. b) Dependence of successive enthalpy change (corrected for HypDH processing)* on n($TBAH_2PO_4$) / n(**3**) ratio. ■ Experimental; — calculated. c) Distribution of **3** and its complexes with dihydrogenphosphate during the titration.

* The correction consisted of the addition of the heat of formation of $(H_2PO_4)_2^{2-}$ present in the volume of the single injection to the measured successive enthalpy changes, according to the procedure described in the work of Horvat et al.[6]

Hydrogen pyrophosphate

Complexation of hydrogen pyrophosphate with ***1***

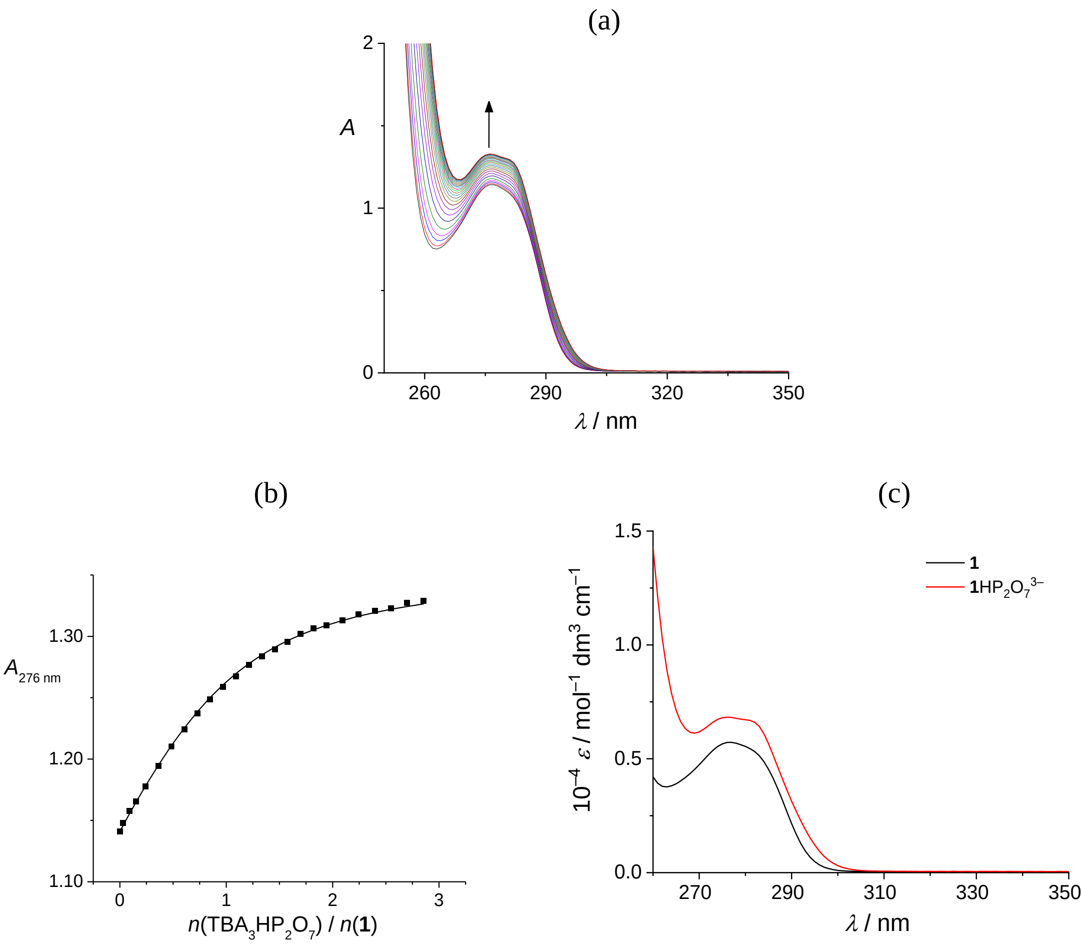

Figure S35. a) Spectrophotometric titration of **1** ($c = 2.00\times10^{-4}$ mol dm^{-3}, $V_0 = 2.3$ mL) with $TBA_3HP_2O_7$ ($c = 2.79\times10^{-3}$ mol dm^{-3}) in acetonitrile. $l = 1$ cm; $\vartheta = (25.0 \pm 0.1)$ °C. The spectra are corrected for dilution. b) Dependence of absorbance at 276 nm on $n(TBA_3HP_2O_7)$ / n(**1**) ratio. ■ Experimental; — calculated, c) Characteristic UV/Vis spectra of **1** and its hydrogen pyrophosphate complex.

(a)

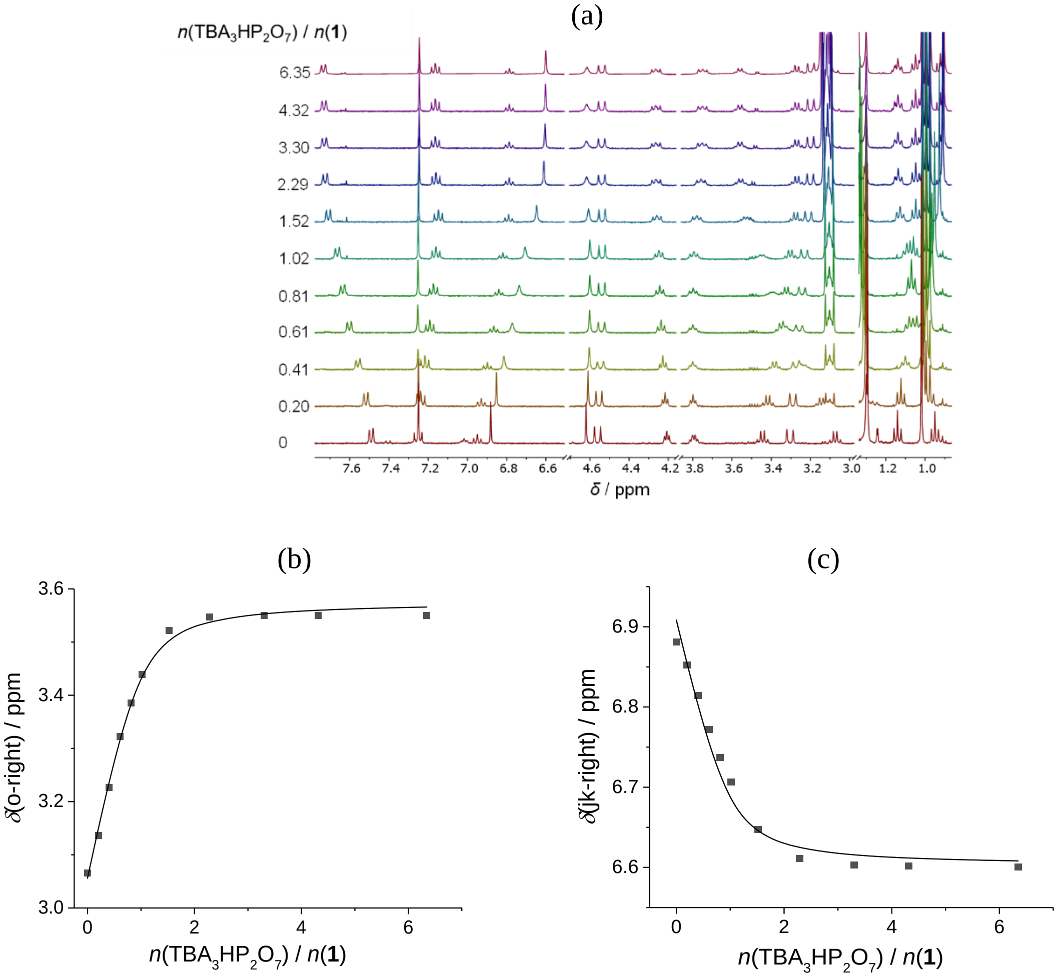

Figure S36. a) ^{1}H NMR spectroscopy titration of **1** (c = 6.40×10^{-4} mol dm^{-3}, V_0 = 500 µL) with $TBA_3HP_2O_7$ (c = 3.254×10^{-2} mol dm^{-3}) in CD_3CN at 25 °C. b), c) Experimental (■), and calculated (—) chemical shifts for selected nuclei at **1**.

Table S15. Calculated ^{1}H NMR chemical shifts (in ppm) for **1** and its complex with hydrogen pyrophosphate in CD_3CN at 25 °C. Assignment of protons is depicted in Figure S5. Left/right assignments (= downfield/upfield, respectively) refer to the position of signal in NMR spectrum when assigning pairs of similar protons.

H	**1**	**1**$HP_2O_7^{3-}$
lm-right	1.0298	0.9075
p-left	1.1353	1.0402
lm-left	1.289	1.3629
o-right	3.0559	3.5776
hi-right	3.3101	3.1962
o-left	3.4444	3.2601
f	4.2073	4.2661
jk-right	6.9083	6.6012
e	6.9554	6.775
c	7.4657	7.7301

(a) (b)

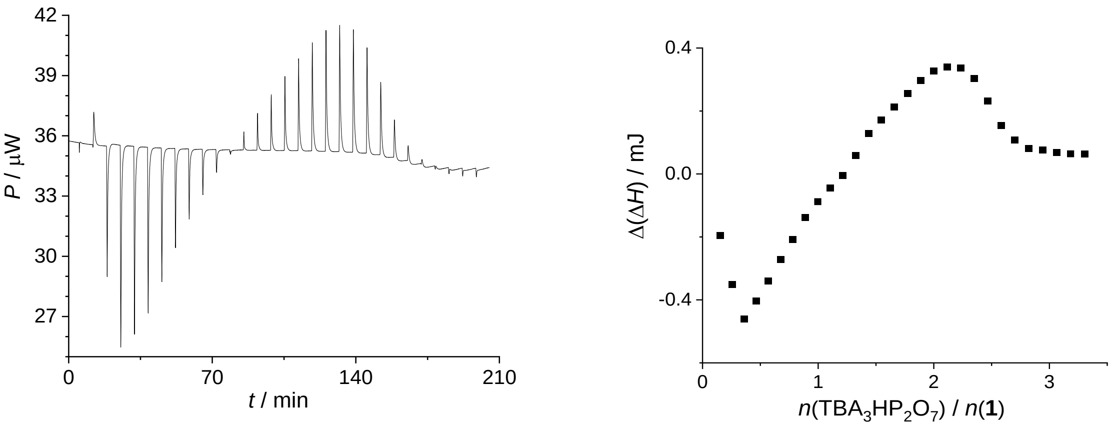

Figure S37. a) Microcalorimetric titration of **1** ($c = 2.26\times10^{-4}$ mol dm^{-3}, $V_0 = 1.4295$ mL) with $TBA_3HP_2O_7$ ($c = 3.281\times10^{-3}$ mol dm^{-3}) in acetonitrile at 25 °C. b) Dependence of successive enthalpy change on $n(TBA_3HP_2O_7)$ / n(**1**) ratio. ■ experimental.

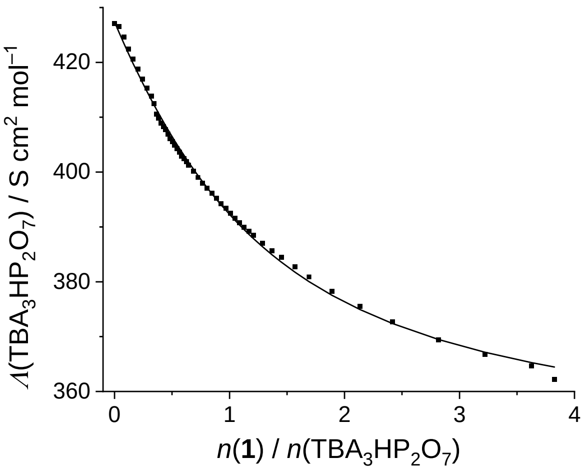

Figure S38. Conductometric titration of $TBA_3HP_2O_7$ (c = 9.92×10^{-5} mol dm^{-3}, V_0 = 15.0 mL) with **1** (c = 5.99×10^{-4} mol dm^{-3}) in acetonitrile at (25.0 ± 0.1) °C. Dependence of molar conductivity of $TBA_3HP_2O_7$ on n(**1**) / n($TBA_3HP_2O_7$)) ratio: ■ experimental; — calculated.

Table S16. Molar conductivities for ions present in titration depicted in Figure S38. Approximation of constant molar ionic conductivities throughout the titration was used. This is valid due to very small variations in ionic strength during the experiment.

	TBA^+	$HP_2O_7^{3-}$	**1**$HP_2O_7^{3-}$
λ_∞/S cm^2 mol^{-1}	61.63[a]	242.5(2)	152.9(5)[b]

[a] Literature data.[7]

[b] Data calculated with HypSpec program during the fitting of the titration curve (Figure S38) with model implying the formation of **1**$HP_2O_7^{3-}$ complex.

*Complexation of hydrogen pyrophosphate with **2***

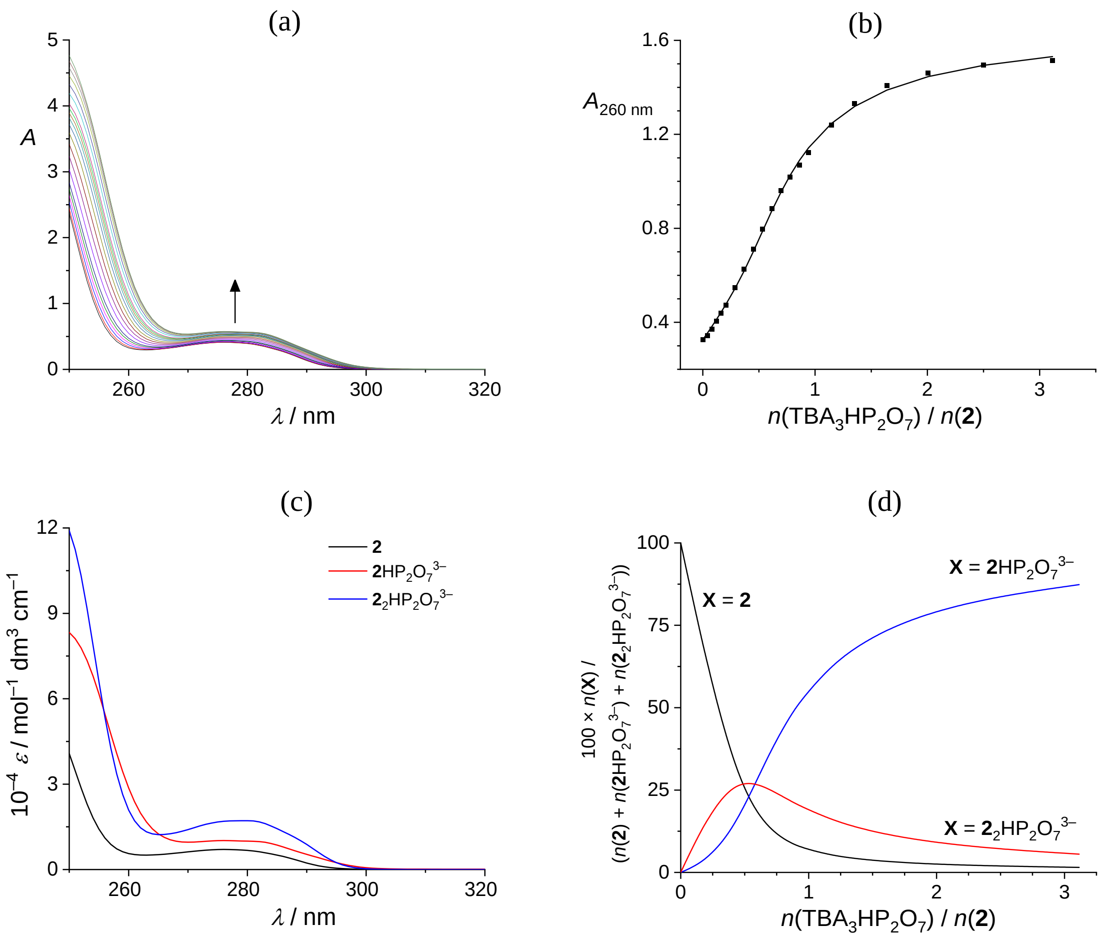

Figure S39. a) Spectrophotometric titration of **2** ($c = 5.83\times10^{-5}$ mol dm^{-3}, $V_0 = 2.3$ mL) with $TBA_3HP_2O_7$ ($c = 5.49\times10^{-4}$ mol dm^{-3}) in acetonitrile. $l = 1$ cm; $\vartheta = (25.0 \pm 0.1)$ °C. The spectra are corrected for dilution. b) Dependence of absorbance at 260 nm on $n(TBA_3HP_2O_7)$ / n(**2**) ratio. ■ experimental; — calculated, c) Characteristic UV/Vis spectra of **2** and its hydrogen pyrophosphate complexes. d) Distribution of **2** and its complexes with hydrogen pyrophosphate during the titration of **2** with $TBA_3HP_2O_7$.

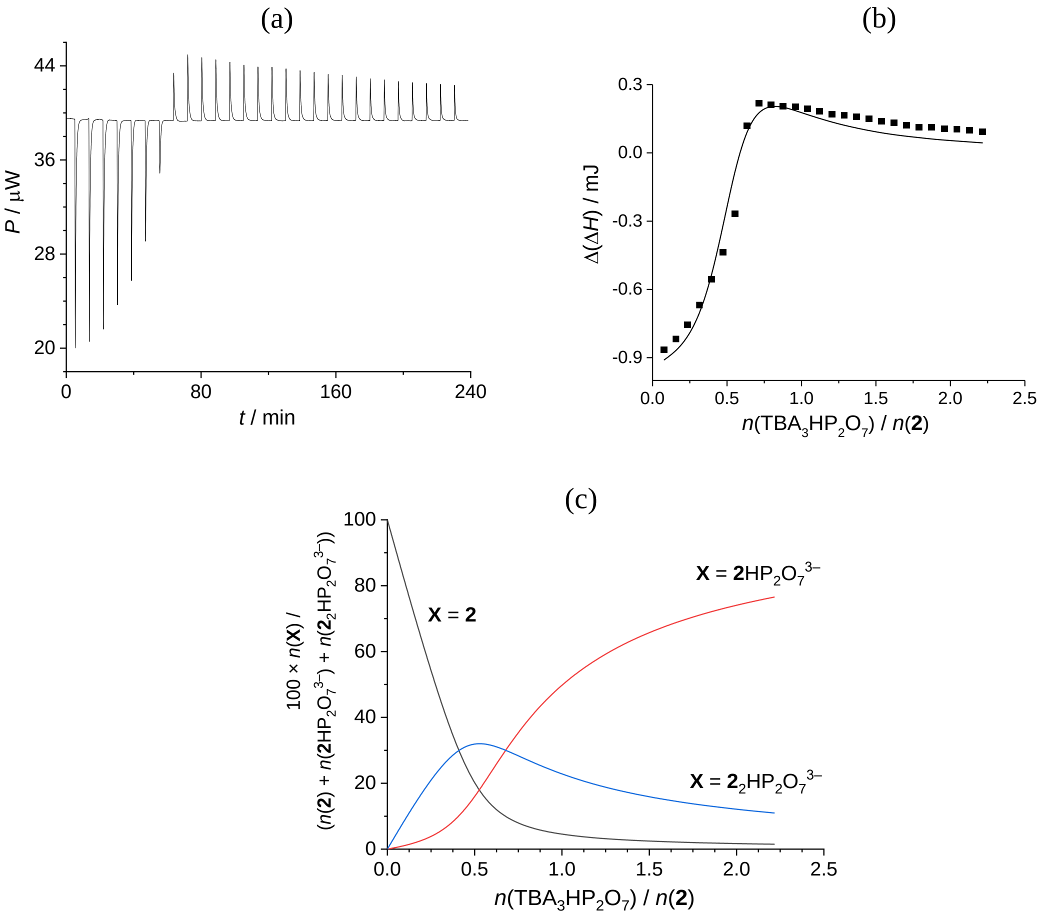

Figure S40. a) Microcalorimetric titration of **2** (c = 6.63×10^{-5} mol dm^{-3}, V_0 = 1.4265 mL) with $TBA_3HP_2O_7$ (c = 1.48×10^{-3} mol dm^{-3}) in acetonitrile at 25 °C. b) Dependence of successive enthalpy change on n($TBA_3HP_2O_7$) / n(**2**) ratio. ■ Experimental; — calculated. c) Distribution of **2** and its complexes with hydrogen pyrophosphate during the titration of **2** with $TBA_3HP_2O_7$.

(a)

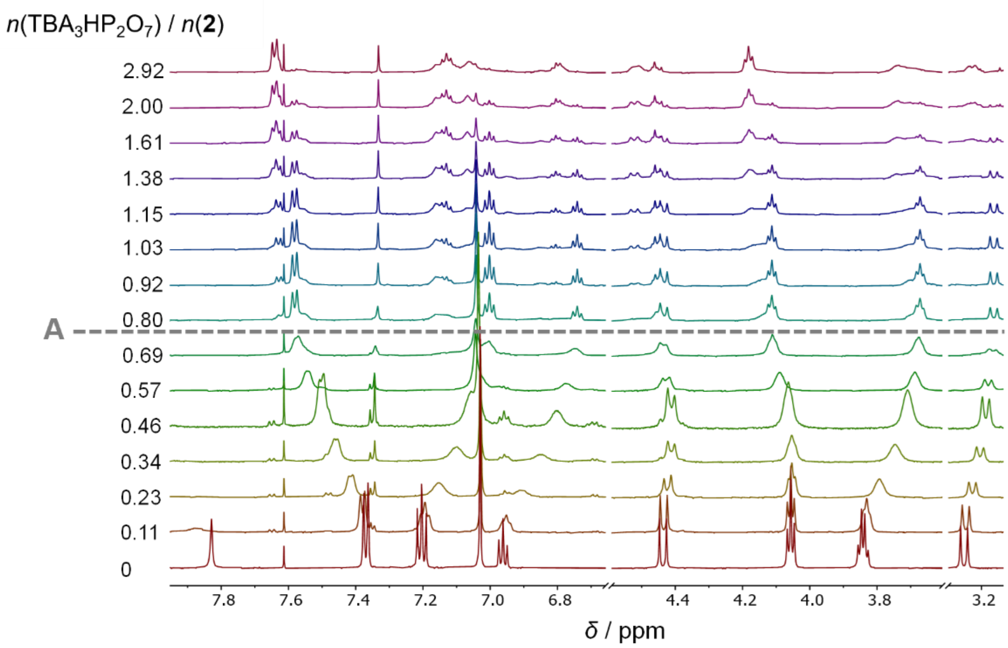

(b)

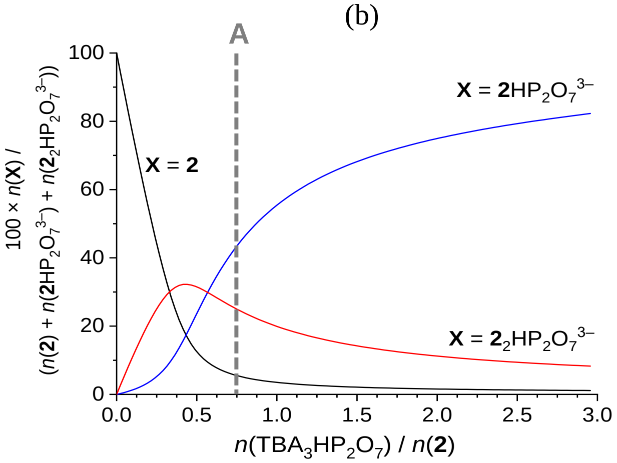

Figure S41. a) ^{1}H NMR spectroscopy titration of **2** ($c = 7.09\times10^{-5}$ mol dm^{-3}, $V_0 = 500$ μL) with $TBA_3HP_2O_7$ ($c = 8.15\times10^{-4}$ mol dm^{-3}) in CD_3CN at 25 °C. b) Distribution of **2** and its complexes with hydrogen pyrophosphate calculated with the values of the appropriate complexation constants obtained by ITC (Figure S40).

*Complexation of hydrogen pyrophosphate with **3***

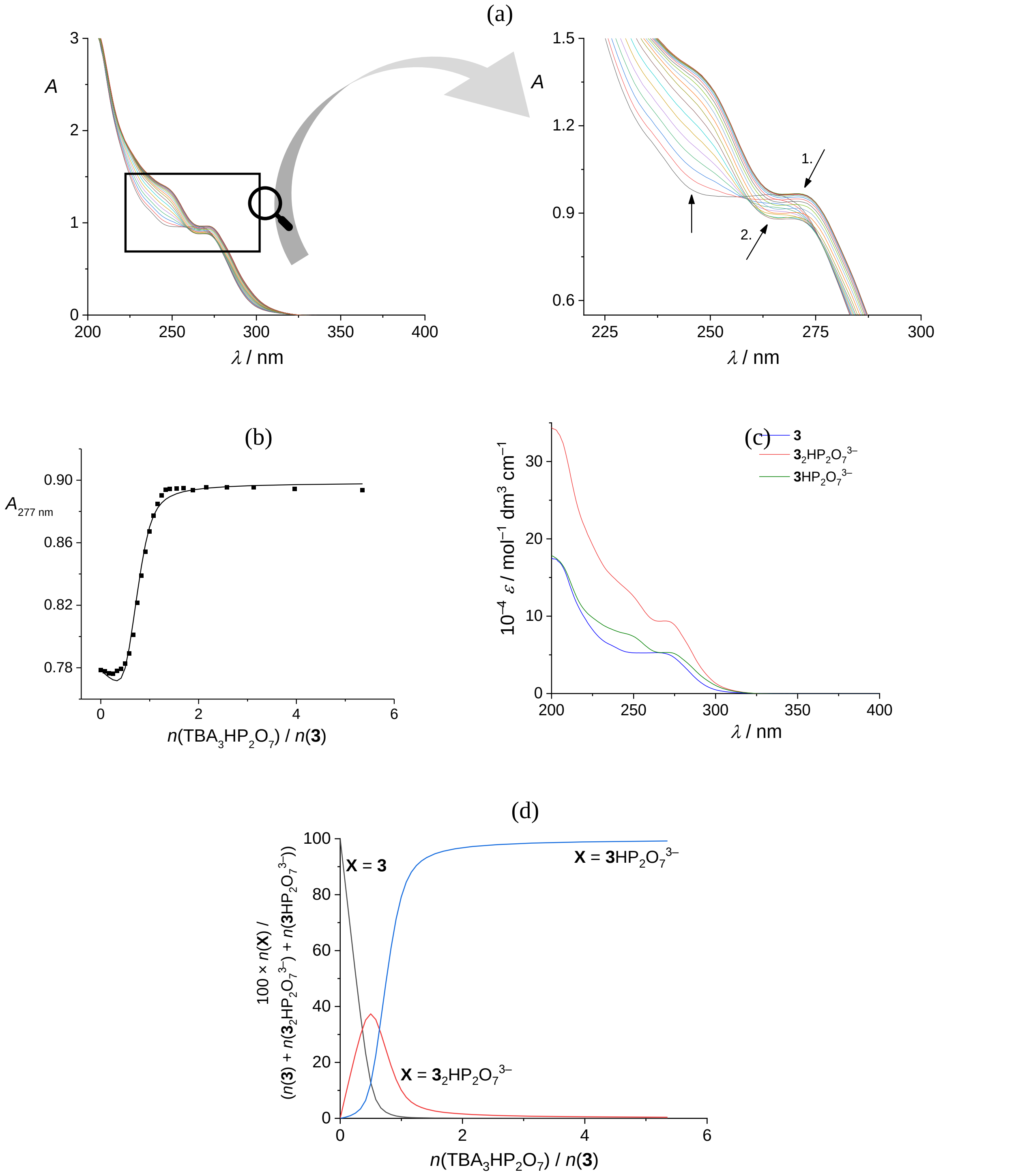

Figure S42. a) Spectrophotometric titration of **3** ($c = 1.82\times10^{-5}$ mol dm^{-3}, $V_0 = 2.3$ mL) with $TBA_3HP_2O_7$ ($c = 5.92\times10^{-4}$ mol dm^{-3}) in acetonitrile. $l = 1$ cm; $\vartheta = (25.0 \pm 0.1)$ °C. The spectra are corrected for dilution. b) Dependence of absorbance at 277 nm on $n(TBA_3HP_2O_7)$ / n(**3**) ratio. ■ experimental; — calculated. c) Characteristic UV/Vis spectra of **3** and its hydrogen pyrophosphate complexes. d) Distribution of **3** and its complexes with hydrogen pyrophosphate during the titration of **3** with $TBA_3HP_2O_7$.

(a)

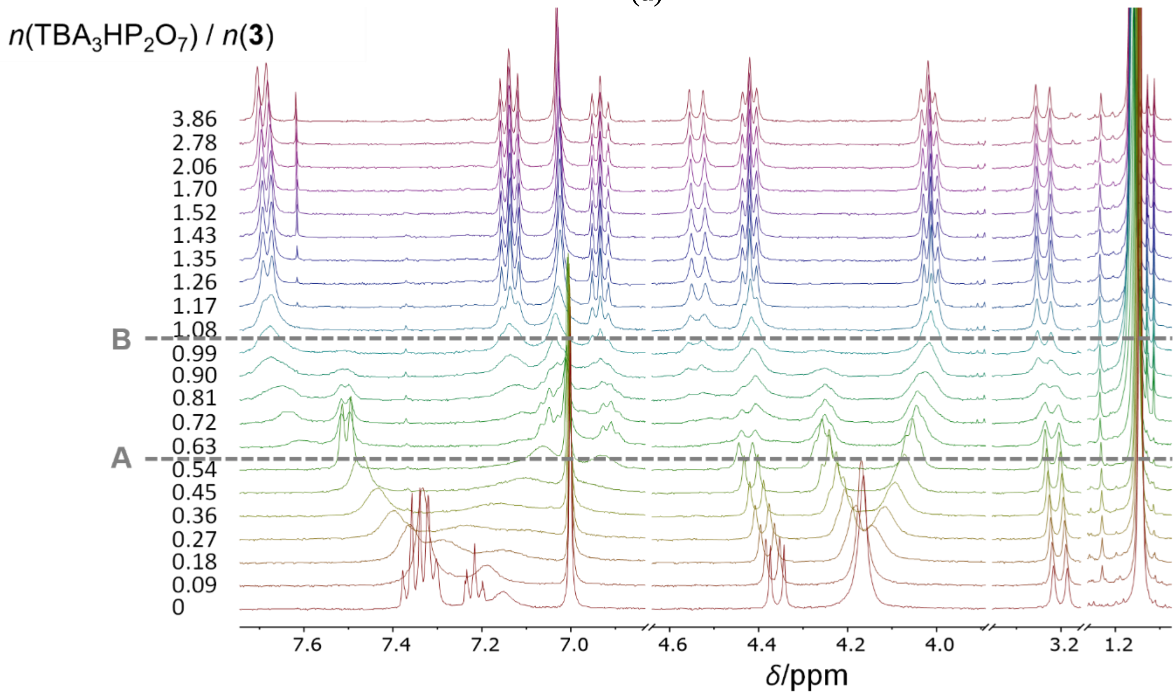

(b)

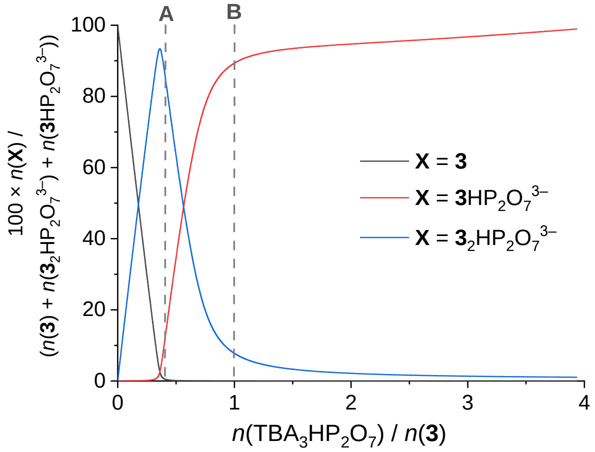

Figure S43. a) ^{1}H NMR spectroscopy titration of **3** (c = 6.58 $\times 10^{-4}$ mol dm^{-3}, V_0 = 500 μL) with $TBA_3HP_2O_7$ (c = 5.90$\times 10^{-3}$ mol dm^{-3}) in CD_3CN at 25 °C. b) Distribution of **3** and its complexes with hydrogen pyrophosphate calculated with the values of the appropriate complexation constants obtained by UV spectrophotometric titration (Figure S42).

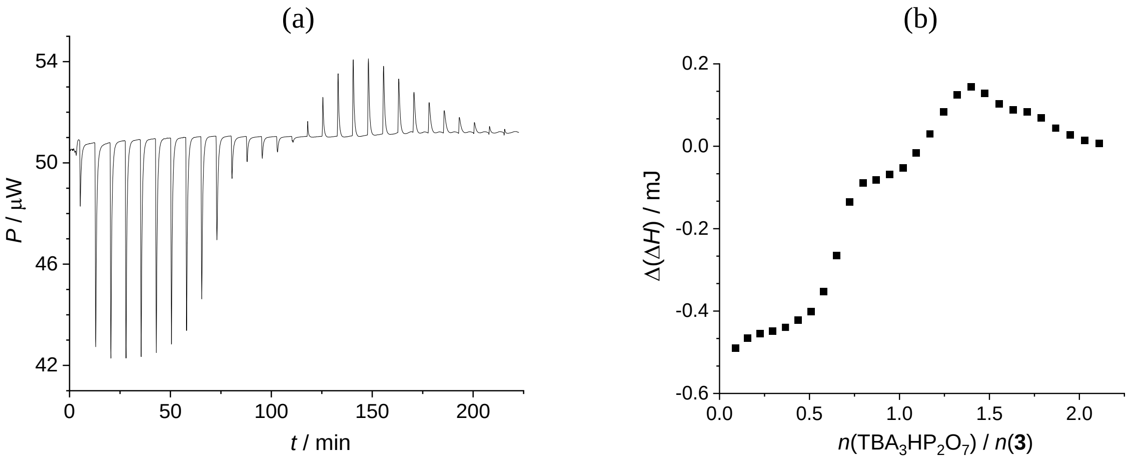

Figure S44. a) Microcalorimetric titration of **3** (c = 5.23×10^{-5} mol dm^{-3}, V_0 = 1.45 mL) with $TBA_3HP_2O_7$ (c = 5.17×10^{-4} mol dm^{-3}) in acetonitrile at 25 °C. b) Dependence of successive enthalpy change on n($TBA_3HP_2O_7$) / n(**3**) ratio.

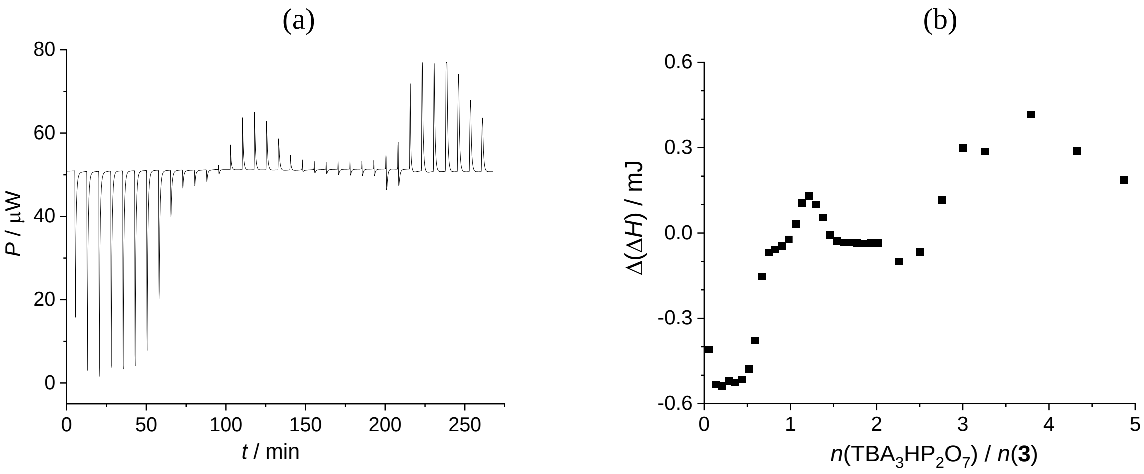

Figure S45. a) Microcalorimetric titration of **3** (c = 2.35×10^{-4} mol dm^{-3}, V_0 = 1.45 mL) with $TBA_3HP_2O_7$ (c = 6.48×10^{-3} mol dm^{-3}) in acetonitrile at 25 °C. b) Dependence of successive enthalpy change on n($TBA_3HP_2O_7$) / n(**3**) ratio. The successive volumes of titrant additions follow this order: 1. 3 μL, 2. – 26. 4 μL, 27. – 31. 12 μL, 32. – 35. 25 μL, 36. 30 μL.

Comparison with other known relevant complexes

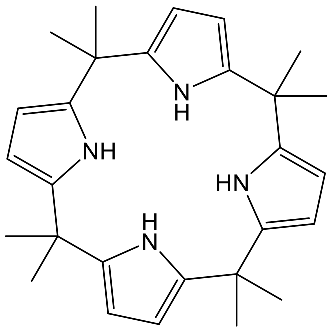

N1

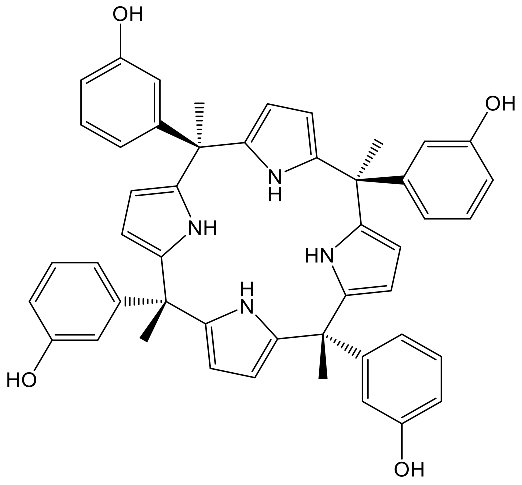

N2

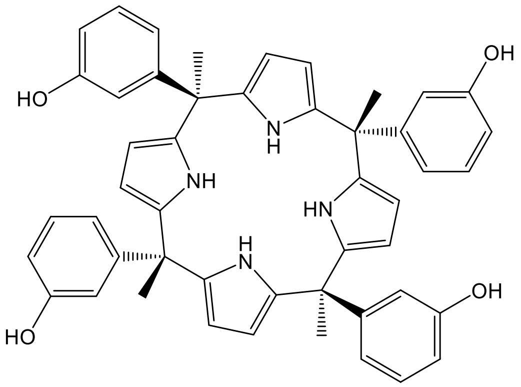

N3

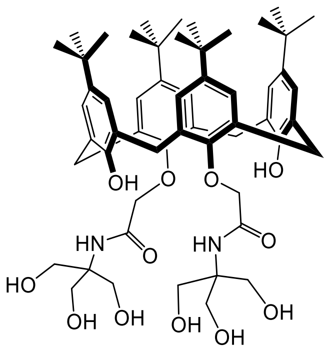

N4

Figure S46. Structures of some of the neutral receptors for hydrogen pyrophosphate in MeCN from the work of Danil de Namor at al.[8–10]

Table S17. Thermodynamic parameters for complexation of hydrogen pyrophosphate (A) with compounds **N1 – N4** (structures given in Figure S46) in acetonitrile at 25 °C.[8–10]

compound	log *β*	$\Delta_r H$ / kJ mol^{-1}	$(-T\cdot\Delta_r S)$ / kJ mol^{-1}
**N1**	**N**$_2$A: 8.61<br>**N**A: 4.17	**N**$_2$A: −273.7<br>**N**A: −89.5	**N**$_2$A: 224.5<br>**N**A: 65.6
**N2**	**N**$_2$A: 6.80<br>**N**A: 3.26	**N**$_2$A: −166<br>**N**A: −38.5	**N**$_2$A: 127<br>**N**A: 20
**N3**	**N**$_2$A: 7.22<br>**N**A: 3.94	**N**$_2$A: −148.6<br>**N**A: −65.4	**N**$_2$A: 107.3<br>**N**A: 42.6
**N4**	**N**$_2$A: 8.61<br>**N**A: 5.10	**N**$_2$A: −116.6<br>**N**A: −59.3	**N**$_2$A: 67.4<br>**N**A: 21.5

Fumarate

Preparation of TBA_2fum salt

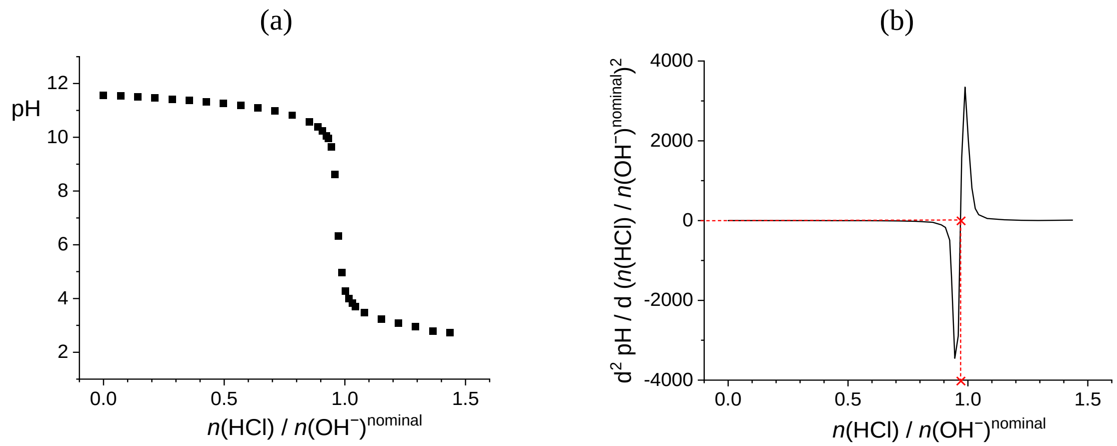

Figure S47. Potentiometric standardization of commercial TBAOH (c^{nominal} = 1.41 mol dm^{-3}, V = 0.100 mL, diluted in 30 mL of H_2O) with commercial (Kefo) standard HCl (aq, 0,1 mol dm^{-3}) at 25 °C. Titration was performed using Titrando 888, Dosino 800, and pH electrode Methohm 6.0280.300 (calibrated with buffers pH = 3, 5, 7, and 9: E/mV = −57.35×pH + 406.0). The real concentration of the analyzed TBAOH solution, obtained from the results of two potentiometric titrations is: c^{real} = (1.39 ± 0.02) mol dm^{-3}.

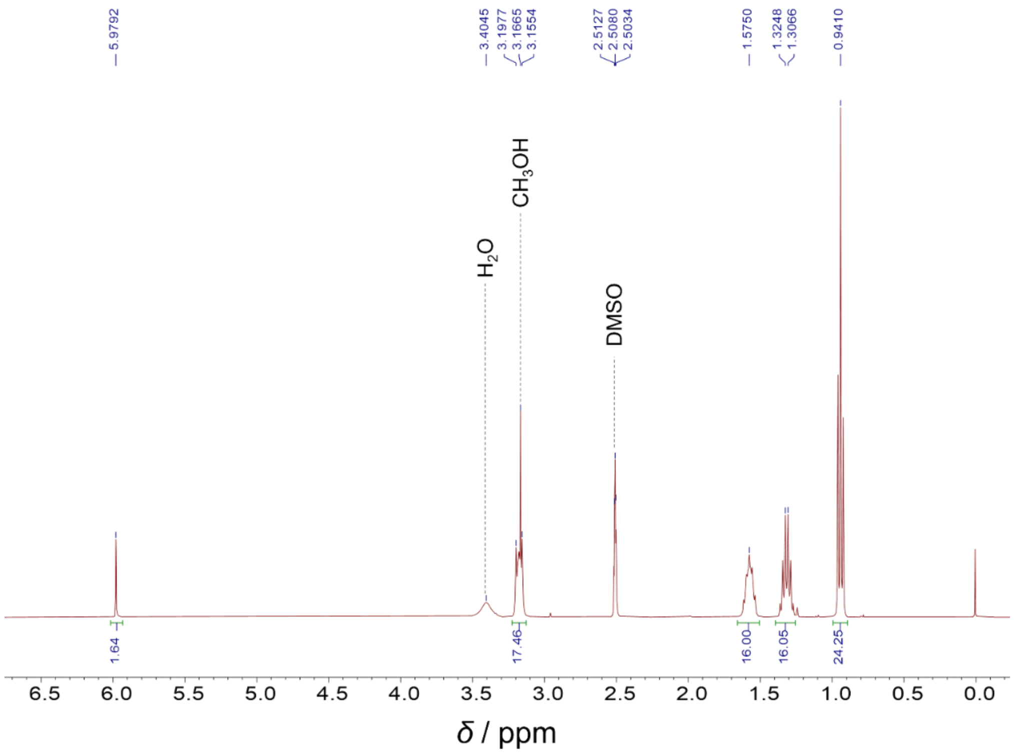

Figure S48. ^{1}H NMR spectrum of compound TBA_2fum in DMSO-d_6 (400 MHz, 298 K).

*Complexation of fumarate with **1***

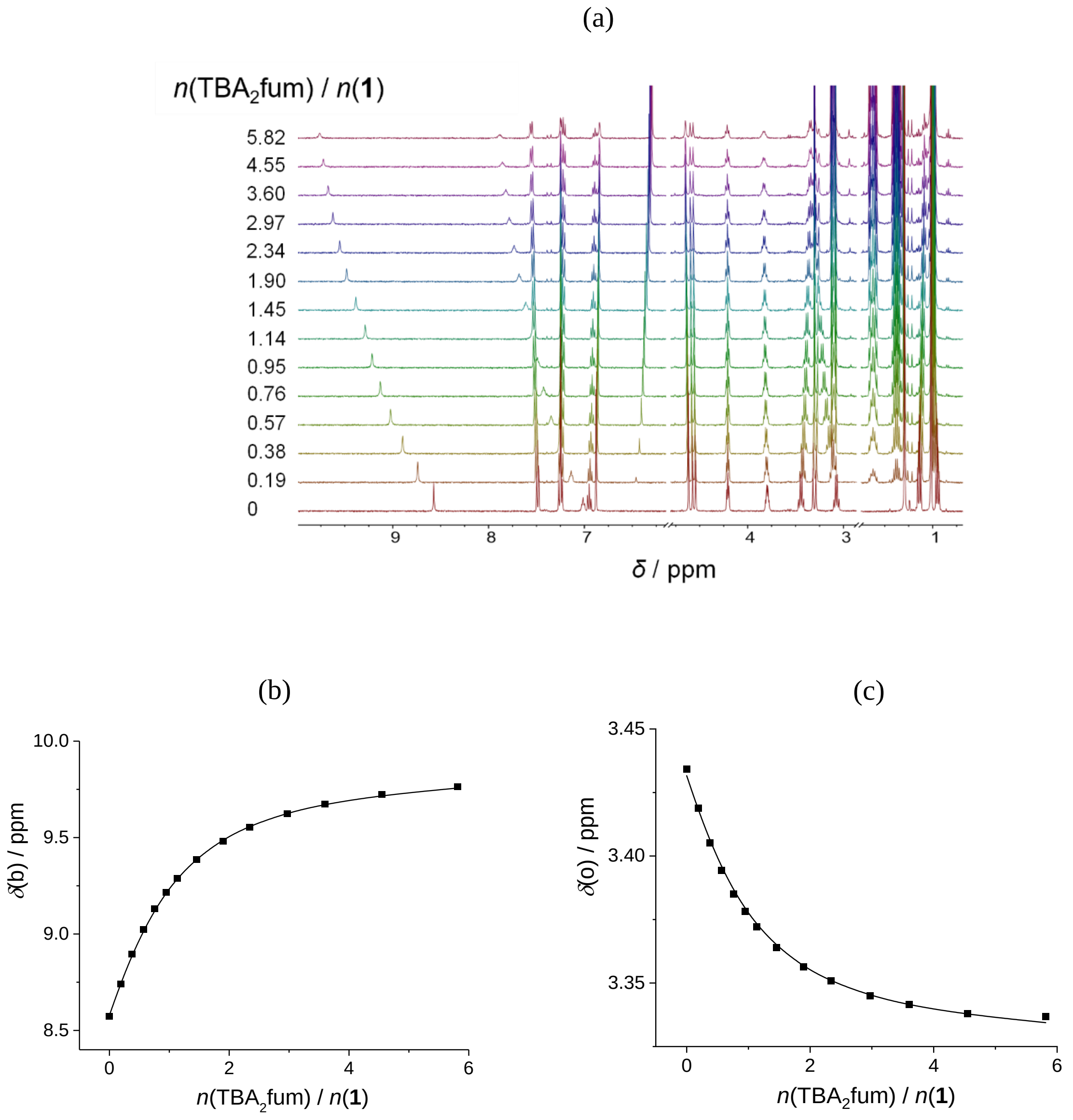

Figure S49. a) ^{1}H NMR spectroscopy titration of **1** (c = 5.59×10^{-4} mol dm^{-3}, V_0 = 500 μL) with TBA_2fum (c = 3.53×10^{-3} mol dm^{-3}) in CD_3CN at 25 °C. b), c) Experimental (■), and calculated (—) chemical shifts for selected nuclei at **1**.

Table S18. Calculated ^{1}H NMR chemical shifts (in ppm) for **1** and its complex with fum^{2-} in CD_3CN at 25 °C. Assignment of protons is depicted in Figure S5. Left/right assignments (= downfield/upfield, respectively) refer to the position of signal in NMR spectrum when assigning pairs of similar protons.

H	**1**	**1**fum^{2-}
c	7.4768	7.5564
b	8.579	10.0042
a	7.0212	8.0625
d	7.2288	7.1935
e	6.948	6.8763
jk-right	6.8784	6.8339
n	4.6171	4.6576
hi-left	4.5742	4.6079
g	3.8037	3.8477
o-left	3.4316	3.3141
hi-right	3.2865	3.2474
p-left	1.1382	1.0722

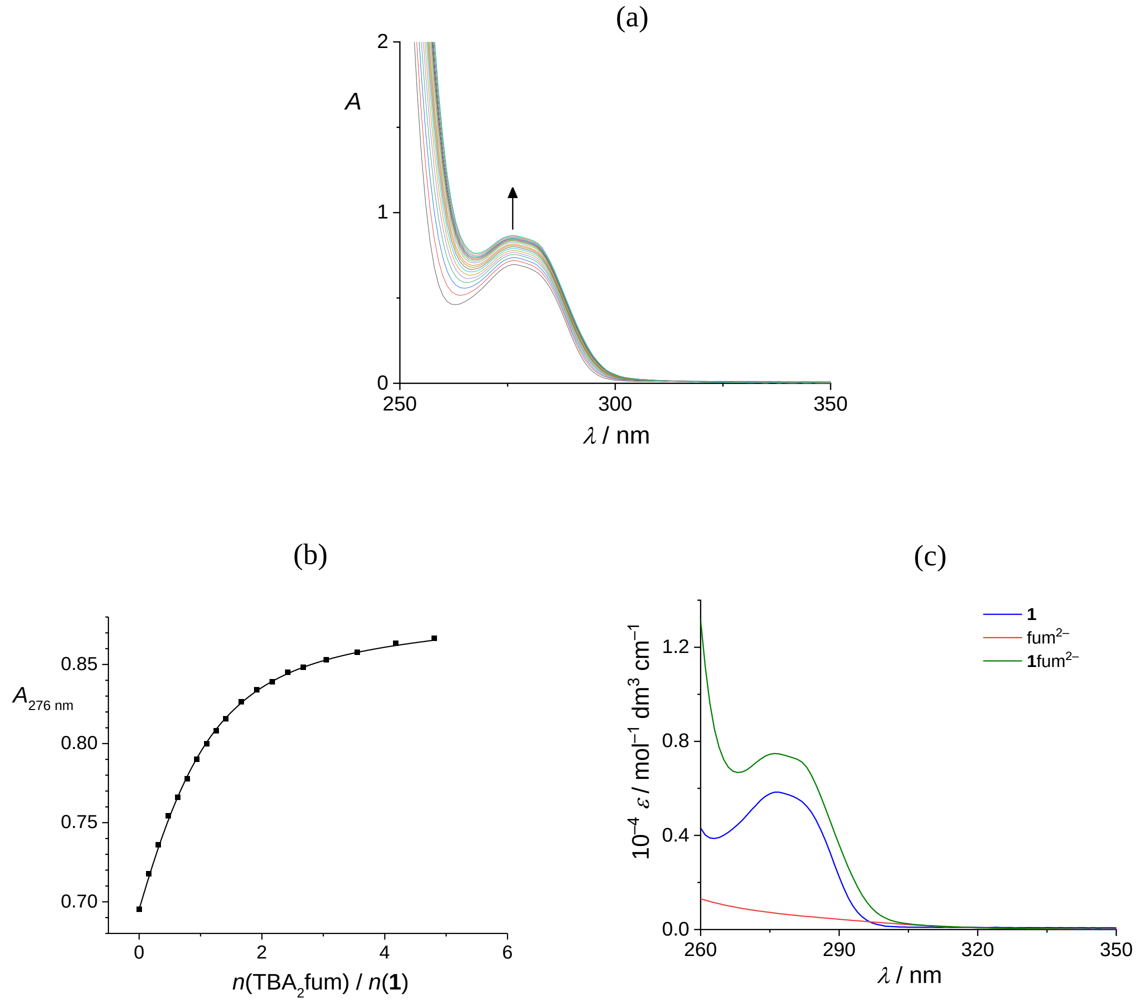

Figure S50. a) Spectrophotometric titration of **1** (c = 1.19×10^{-4} mol dm^{-3}, V_0 = 2.3 mL) with TBA_2fum (c = 1.72×10^{-3} mol dm^{-3}) in acetonitrile. l = 1 cm; ϑ = (25.0 ± 0.1) °C. The spectra are corrected for the absorbance of free TBA_2fum and for the dilution of **1**. b) Dependence of the absorbance at 276 nm on $n(TBA_2fum) / n(\mathbf{1})$ ratio. ■ Experimental; — calculated. c) Characteristic UV spectra of **1**, fum^{2-} and the complex **1**fum^{2-}.

(a) (b)

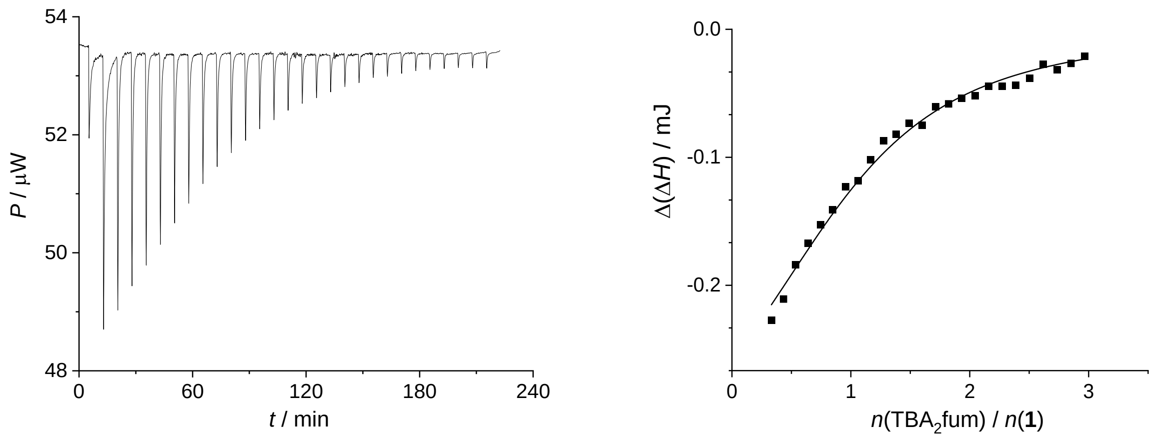

Figure S51. a) Microcalorimetric titration of **1** (c = 1.19×10^{-4} mol dm^{-3}, V_0 = 1.45 mL) with TBA_2fum (c = 1.71×10^{-3} mol dm^{-3}) in acetonitrile at 25 °C. b) Dependence of successive enthalpy change on n(TBA_2fum) / n(**1**) ratio. ■ Experimental; — calculated.

*Complexation of fumarate with **2***

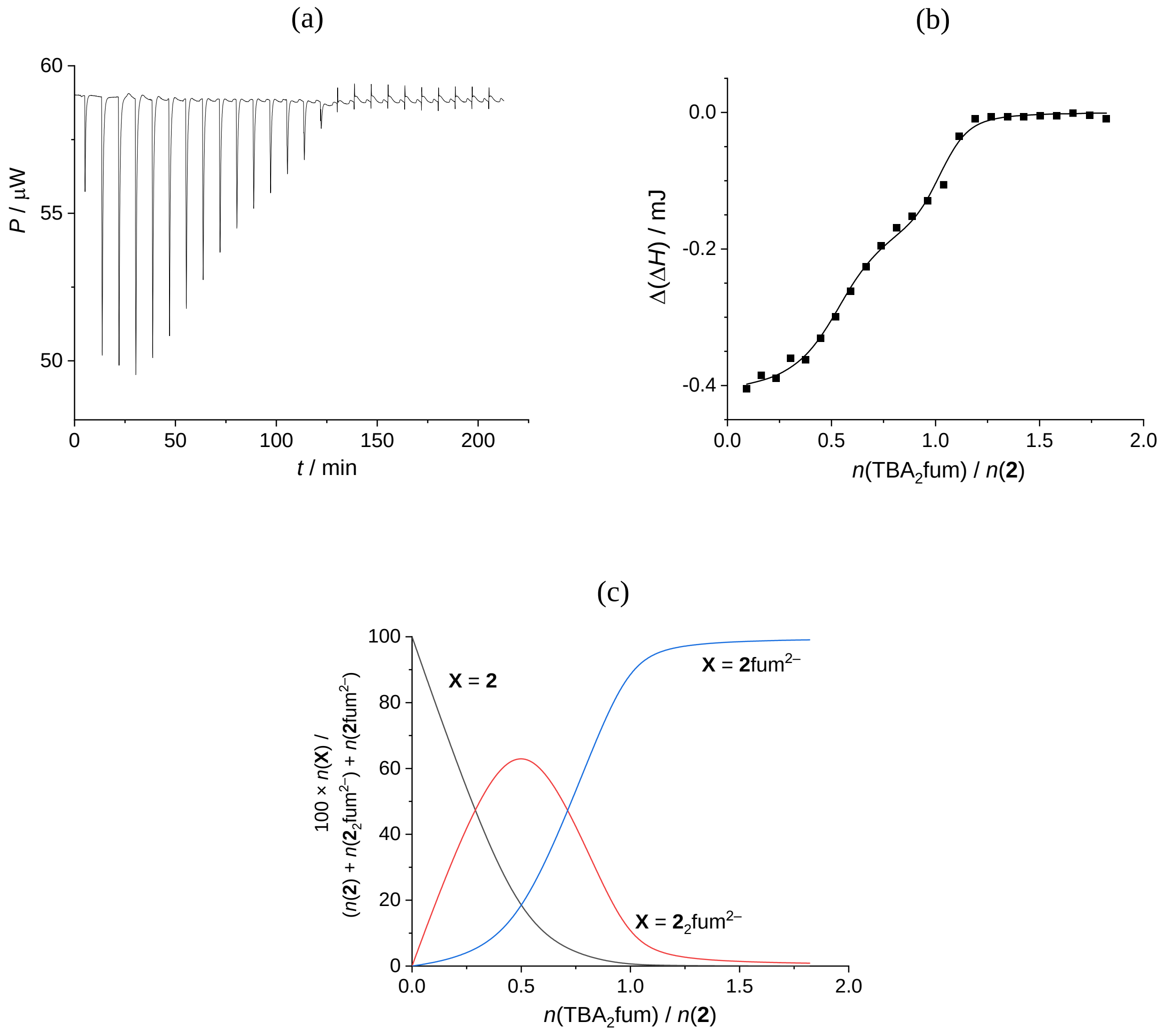

Figure S52. a) Microcalorimetric titration of **2** (c = 7.20×10^{-5} mol dm^{-3}, V_0 = 1.43 mL) with TBA_2fum (c = 7.91×10^{-4} mol dm^{-3}) in acetonitrile at 25 °C. b) Dependence of successive enthalpy change on n(TBA_2fum) / n(**2**) ratio. ■ Experimental; — calculated. c) Distribution of **2** and its complexes with fumarate.

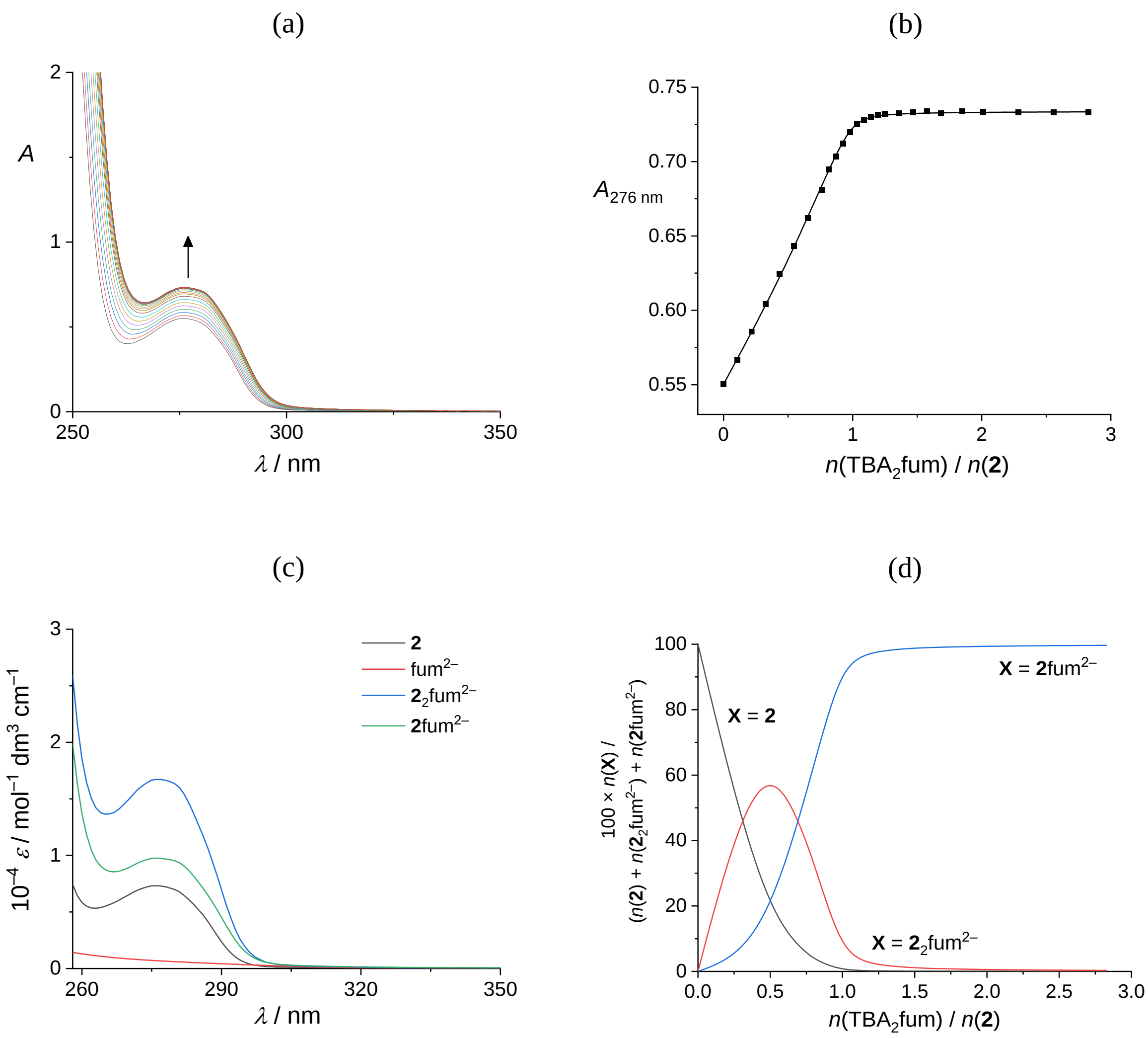

Figure S53. a) Spectrophotometric titration of **2** ($c = 7.52\times10^{-5}$ mol dm^{-3}, $V_0 = 2.3$ mL) with TBA_2fum ($c = 9.40\times10^{-4}$ mol dm^{-3}) in acetonitrile. $l = 1$ cm; $\vartheta = (25.0 \pm 0.1)$ °C. The spectra are corrected for the absorbance of free TBA_2fum and for the dilution of **2**. b) Dependence of the absorbance at 276 nm on $n(TBA_2fum)$ / n(**2**) ratio. ■ Experimental; — calculated. c) Characteristic UV spectra of **2**, fum^{2-}, **2**$_2fum^{2-}$, and **2**fum^{2-}. d) Distribution of **2** and its complexes with fumarate.

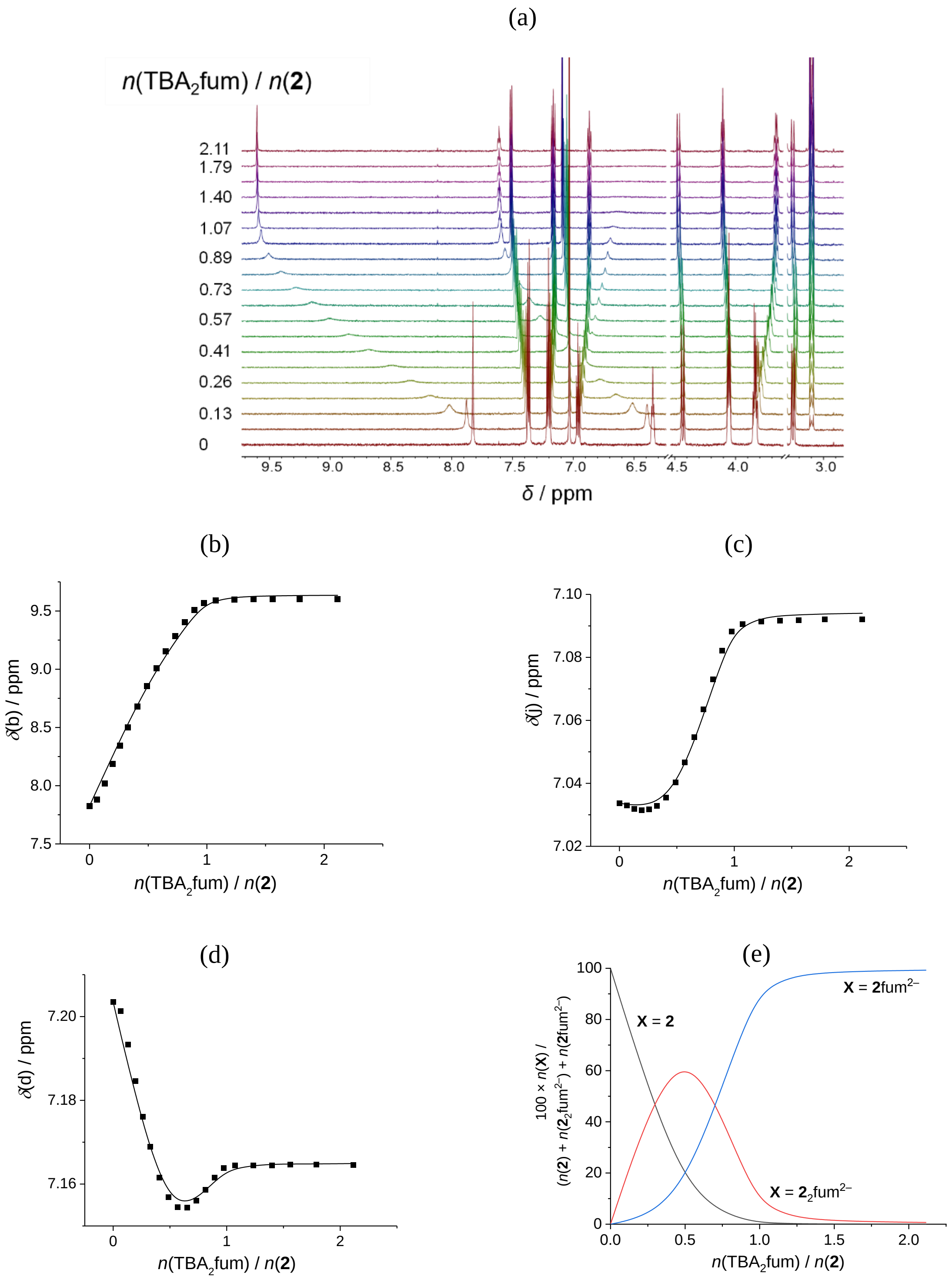

Figure S54. a) ^{1}H NMR spectroscopy titration of **2** ($c = 7.28\times10^{-5}$ mol dm^{-3}, $V_0 = 500$ µL) with TBA_2fum ($c = 2.37\times10^{-4}$ mol dm^{-3}) in CD_3CN at 25 °C. b), c), d) Experimental (■), and calculated (—) chemical shifts for selected nuclei at **2**. e) Distribution of **2** and its complexes with fumarate.

Table S19. Calculated ^{1}H NMR chemical shifts (in ppm) for **2** and its complexes with fum^{2-} in CD_3CN at 25 °C. Assignment of protons is depicted in Figure S5.

H	**2**	**2**$_2$$fum^{2-}$	**2**fum^{2-}
a	6.3463	7.2889	7.6305
b	7.826	8.9518	9.6407
c	7.3612	7.4502	7.5078
d	7.2035	7.1405	7.165
e	6.962	6.8713	6.8672
f	4.055	4.0529	4.108
g	3.8411	3.6926	3.6625
h	3.252	3.2148	3.2552
i	4.4244	4.4217	4.4614
j	7.0337	7.0264	7.0945
k	1.1423	1.1374	1.1658

*Complexation of fumarate with **3***

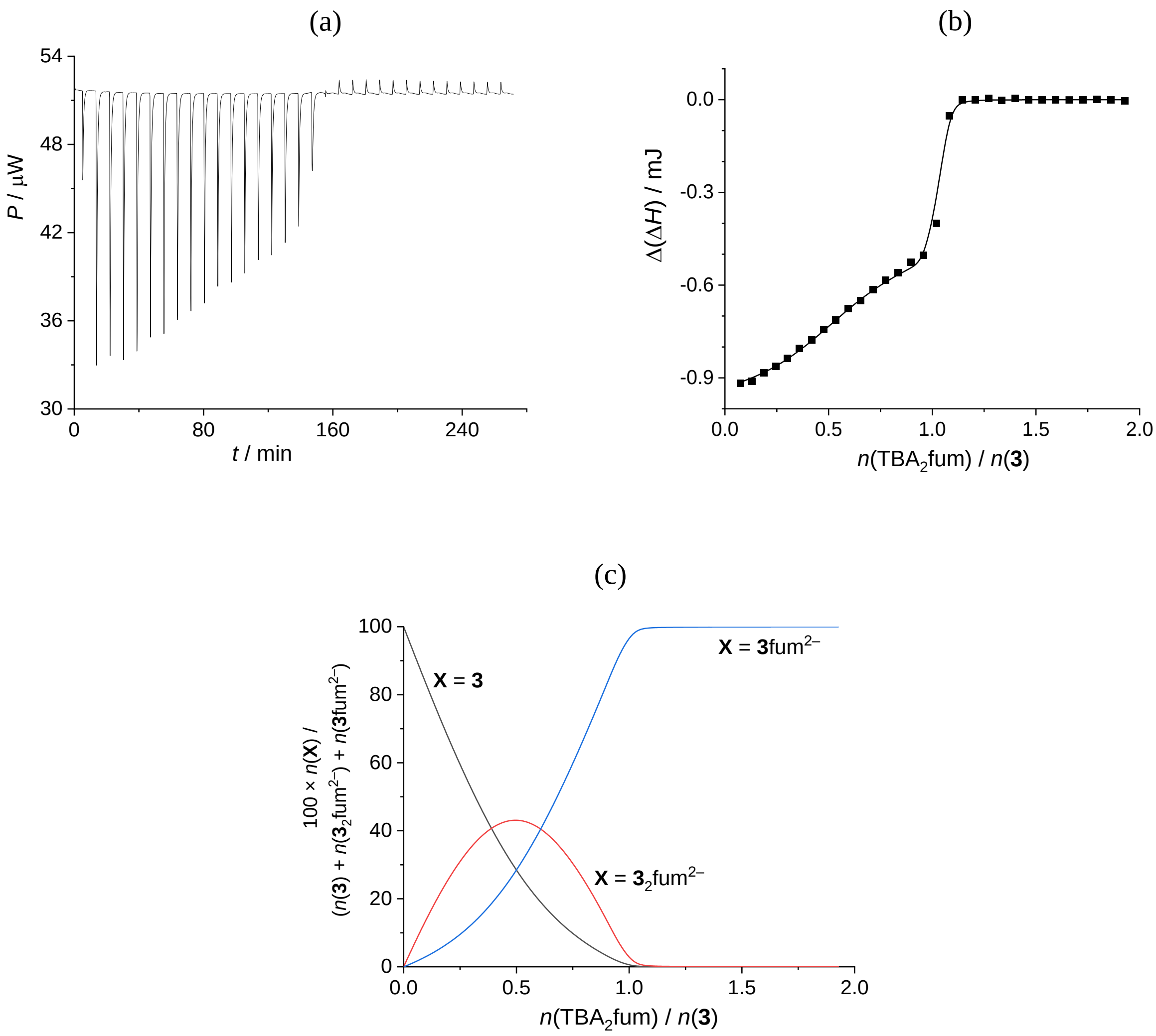

Figure S55. a) Microcalorimetric titration of **3** (c = 2.45×10^{-4} mol dm^{-3}, V_0 = 1.45 mL) with TBA$_2$fum (c = 2.20×10^{-3} mol dm^{-3}) in acetonitrile at 25 °C. b) Dependence of successive enthalpy change on n(TBA$_2$fum) / n(**3**) ratio. c) Distribution of **3** and its complexes with fumarate.

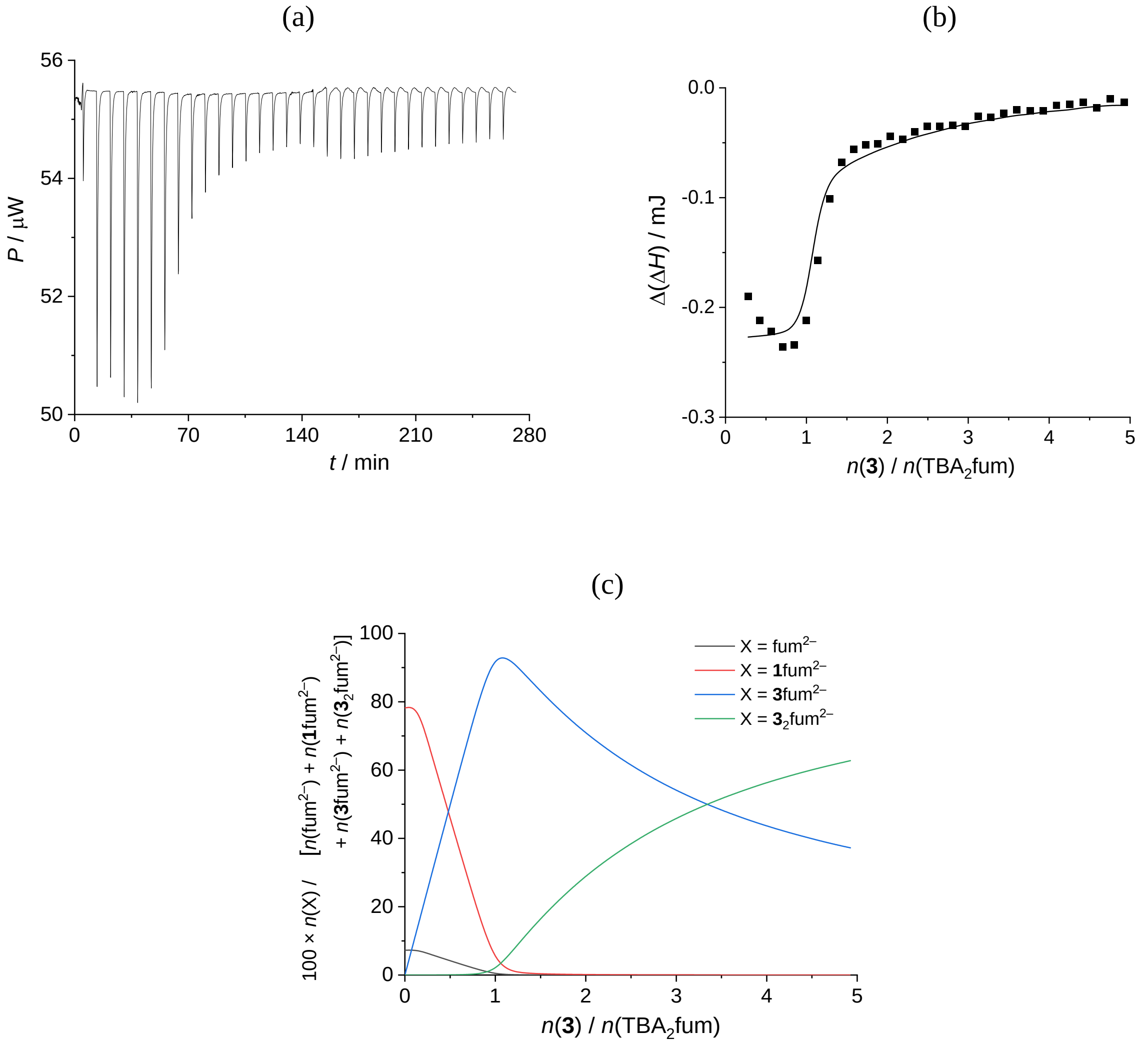

Figure S56. a) Microcalorimetric titration of TBA_2fum (c = 6.91×10^{-5} mol dm^{-3}, V_0 = 1.45 mL; saturated with **1**: c = 7.03×10^{-4} mol dm^{-3}) with **3** (c = 1.56×10^{-3} mol dm^{-3}) in acetonitrile at 25 °C. b) Dependence of successive enthalpy change on n(**3**) / n(TBA_2fum) ratio. c) Distribution of fum^{2-} and its complexes with **1** and **3**.

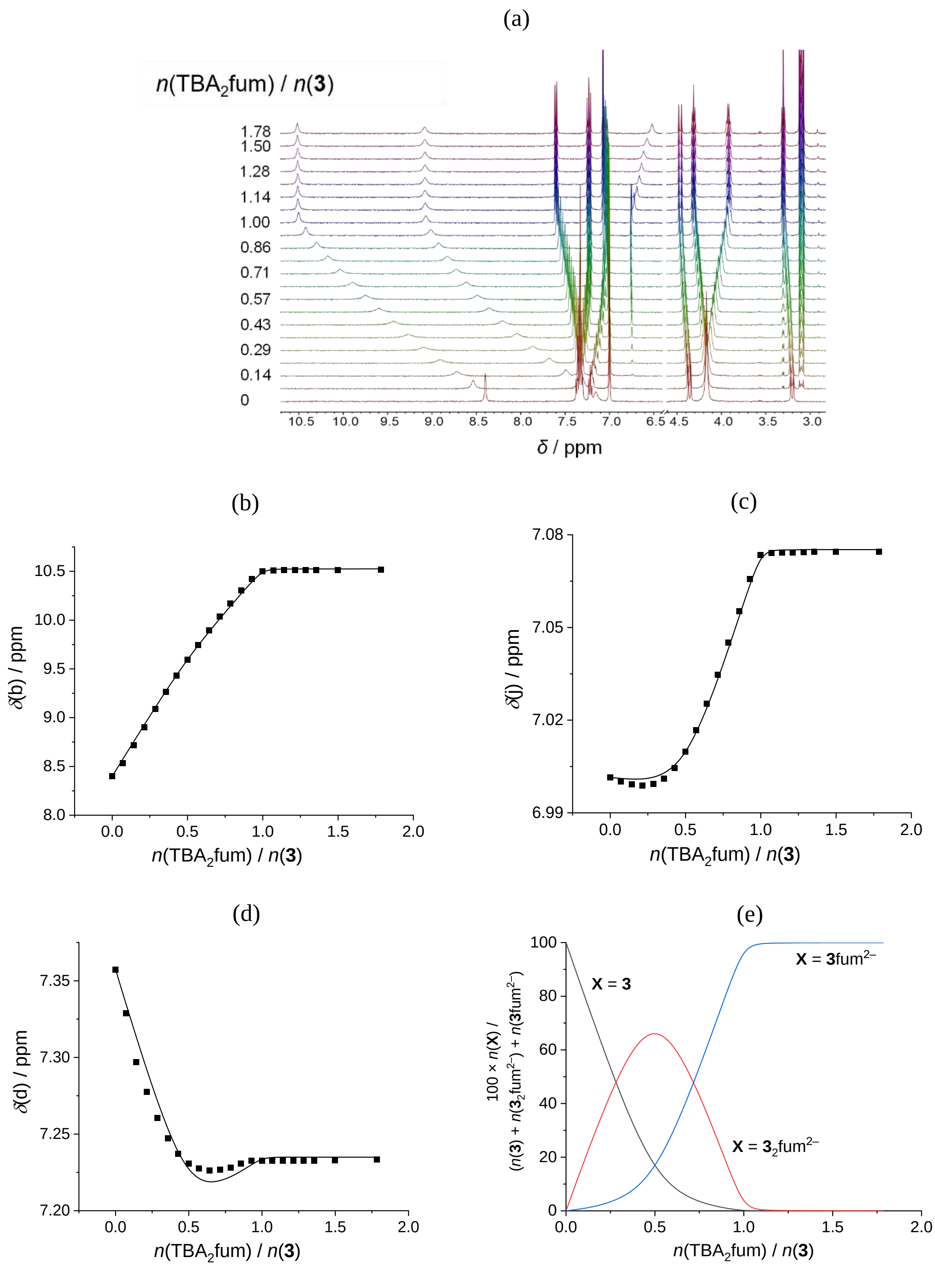

Figure S57. a) ^{1}H NMR spectroscopy titration of **3** ($c = 7.67\times10^{-4}$ mol dm^{-3}, $V_0 = 500$ µL) with TBA_2fum ($c = 5.47\times10^{-3}$ mol dm^{-3}) in CD_3CN at 25 °C. b), c), d) Experimental (■), and calculated (—) chemical shifts for selected nuclei at **3**. e) Distribution of **3** and its complexes with fumarate.

Table S20. Calculated ^{1}H NMR chemical shifts (in ppm) for **3** and its complexes with fum^{2-} in CD_3CN at 25 °C. Assignment of protons is depicted in Figure S1. Left/right assignments (= downfield/upfield, respectively) refer to the position of signal in NMR spectrum when assigning pair of similar protons.

H	**3**	**3**$_2$fum^{2-}	**3**fum^{2-}
b	8.3995	9.6618	10.526
c	7.3025	7.4233	7.5948
d	7.3573	7.1918	7.235
e	7.2167	7.0456	7.0445
a	7.1542	8.4754	9.0875
j	7.0015	6.9951	7.0753
hi-left	4.3741	4.4385	4.4801
f	4.1527	4.2344	4.3152
g	4.1954	4.0748	3.9159
hi-right	3.1863	3.2212	3.2884
k	1.1442	1.1431	1.1709

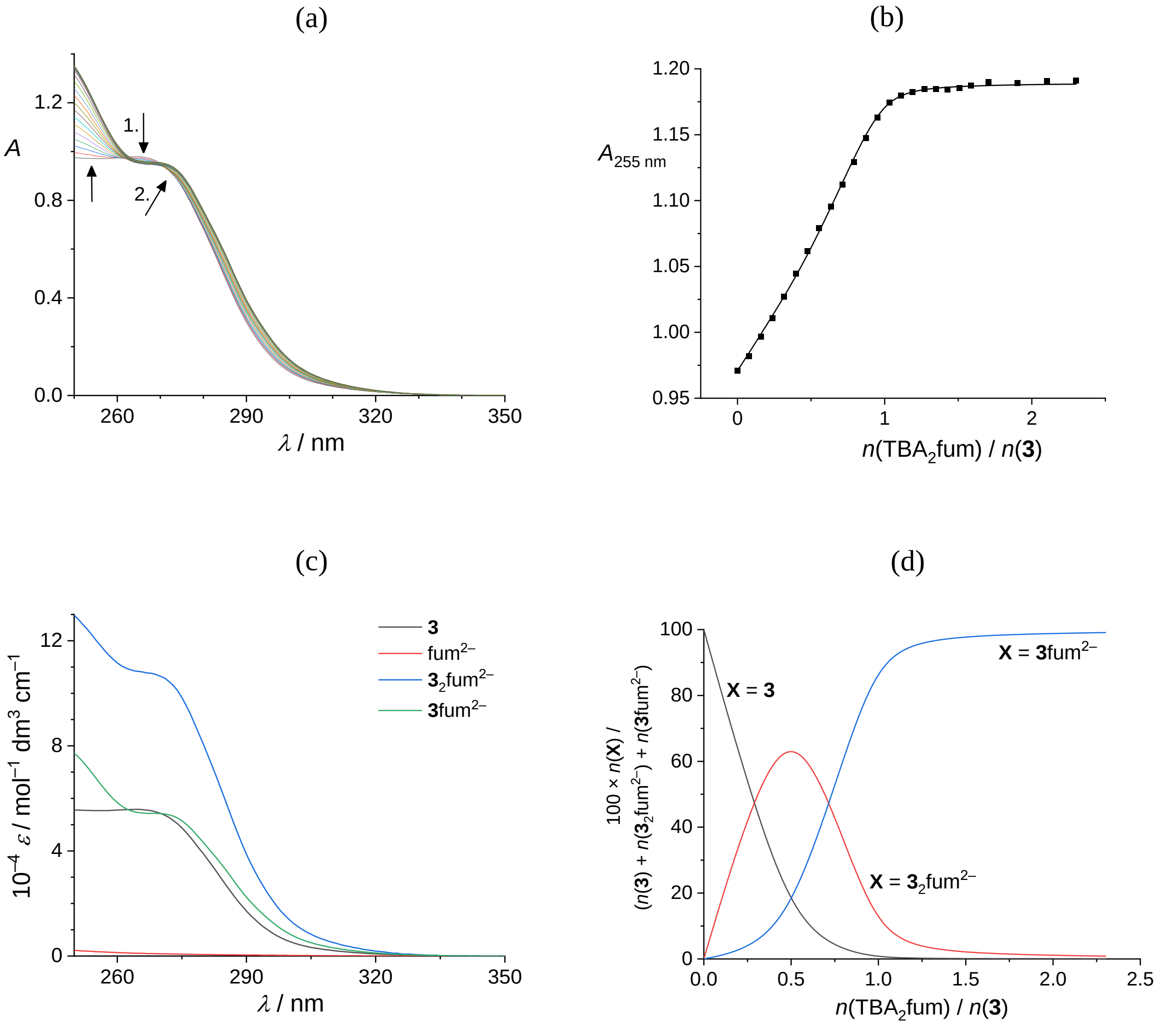

Figure S58. a) Spectrophotometric titration of **3** (c = 1.75×10^{-5} mol dm^{-3}, V_0 = 2.3 mL) with TBA$_2$fum (c = 1.60×10^{-4} mol dm^{-3}) in acetonitrile. l = 1 cm; ϑ = (25.0 ± 0.1) °C. The spectra are corrected for the absorbance of free TBA$_2$fum and for the dilution of **3**. b) Dependence of the absorbance at 255 nm on n(TBA$_2$fum) / n(**3**) ratio. ■ Experimental; — calculated, c) Characteristic UV spectra of **3**, fum^{2-}, **3**$_2$fum^{2-}, and **3**fum^{2-}. d) Distribution of **3** and its complexes with fumarate.

Test of binding of several acids (protonated forms of investigated anions) with 3

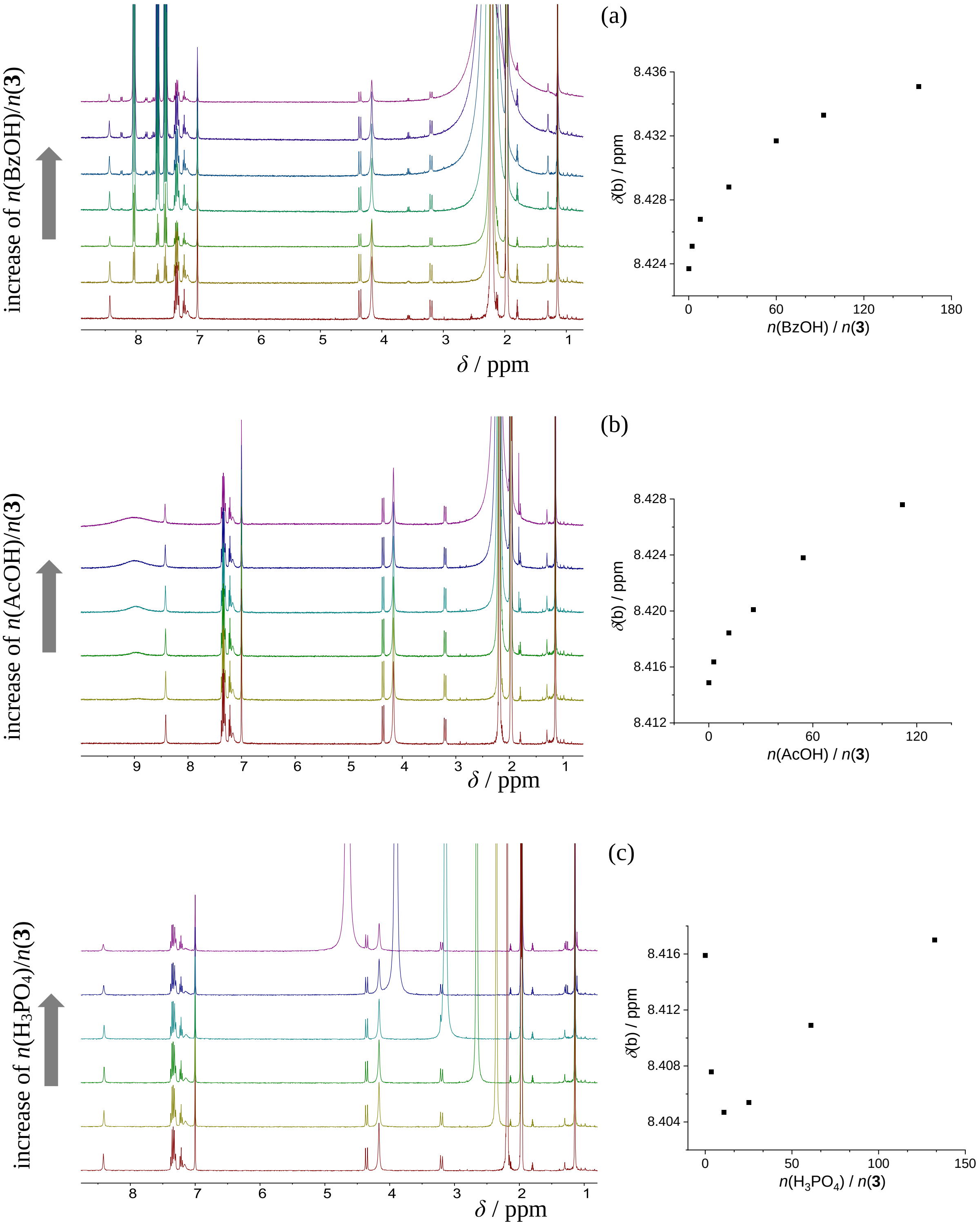

Figure S59. ^{1}H NMR spectroscopy titration of: a) **3** (c = 2.87 ×10^{-4} mol dm^{-3}, V_0 = 500 μL) with BzOH (c_1 = 3.25×10^{-2} mol dm^{-3}, c_2 = 1.87×10^{-1} mol dm^{-3}), b) **3** (c = 5.96 ×10^{-4} mol dm^{-3}, V_0 = 450 μL) with AcOH (c = 1.54×10^{-1} mol dm^{-3}), c) **3** (c = 5.96 ×10^{-4} mol dm^{-3}, V_0 = 450 μL) with H_3PO_4 (c = 1.92×10^{-1} mol dm^{-3}), in CD_3CN at 25 °C. Right: NMR spectra. Left: Chemical shifts of ureido-protons at **3**. In all three cases changes of chemical shifts are negligible (< 0.02 ppm) which suggests no complexation of the tested acids with calixarene **3** is present.

Acetate

*Complexation of acetate with **3***

Table S21. Models used for fitting data obtained from titrations of **3** with TBAOAc. Here are presented real values of log β, whereas in HYPNMR and HypSpec program reduced values were used (for models D and E) according to the procedure described in chapter: Dealing with high values of protonation constants in HYPNMR. In both cases (D and E) the values of characteristic chemical shifts for **3**, **3**$^{-}$ and **3**$^{2-}$ were fixed based on the results from the NMR titration of **3** with DBU (Figure S8).

reaction	log β	Value fixed or refined during fitting?
**Model C**		
$\mathbf{3} + AcO^{-} \rightleftarrows \mathbf{3}AcO^{-}$	3.84[a]<br>7.03[b]	refined
$\mathbf{3} + 2\ AcO^{-} \rightleftarrows \mathbf{3}AcO_2^{2-}$	7.46[a]<br>11.65[b]	refined
**Model D**		
$\mathbf{3}^{2-} + H^{+} \rightleftarrows \mathbf{3}^{-}$	24.28	fixed
$\mathbf{3}^{2-} + 2\ H^{+} \rightleftarrows \mathbf{3}$	47.12	fixed
$H^{+} + AcO^{-} \rightleftarrows AcOH$	22.23	fixed
$MeCN \rightleftarrows MeCN^{-} + H^{+}$	−39	fixed
$\mathbf{3}^{2-} + 2\ H^{+} + AcO^{-} \rightleftarrows \mathbf{3}AcO^{-}$	51.92[c]	refined
$\mathbf{3}^{2-} + 2\ H^{+} + 2\ AcO^{-} \rightleftarrows \mathbf{3}AcO_2^{2-}$	55.08[c]	refined
**Model E**		
$\mathbf{3}^{2-} + H^{+} \rightleftarrows \mathbf{3}^{-}$	24.28	fixed
$\mathbf{3}^{2-} + 2\ H^{+} \rightleftarrows \mathbf{3}$	47.12	fixed
$H^{+} + AcO^{-} \rightleftarrows AcOH$	22.23	fixed
$H^{+} + 2\ AcO^{-} \rightleftarrows AcOH{\cdot}AcO^{-}$	26.63	fixed
$MeCN \rightleftarrows MeCN^{-} + H^{+}$	−39	fixed
$\mathbf{3}^{2-} + 2\ H^{+} + AcO^{-} \rightleftarrows \mathbf{3}AcO^{-}$	50.96[b]<br>54.71[b]	fixed
$\mathbf{3}^{2-} + 2\ H^{+} + 2\ AcO^{-} \rightleftarrows \mathbf{3}AcO_2^{2-}$	55.12[c]<br>59.79[b]	refined

Values obtained by refinement of [a]ITC, [b]UV, [c]NMR data.

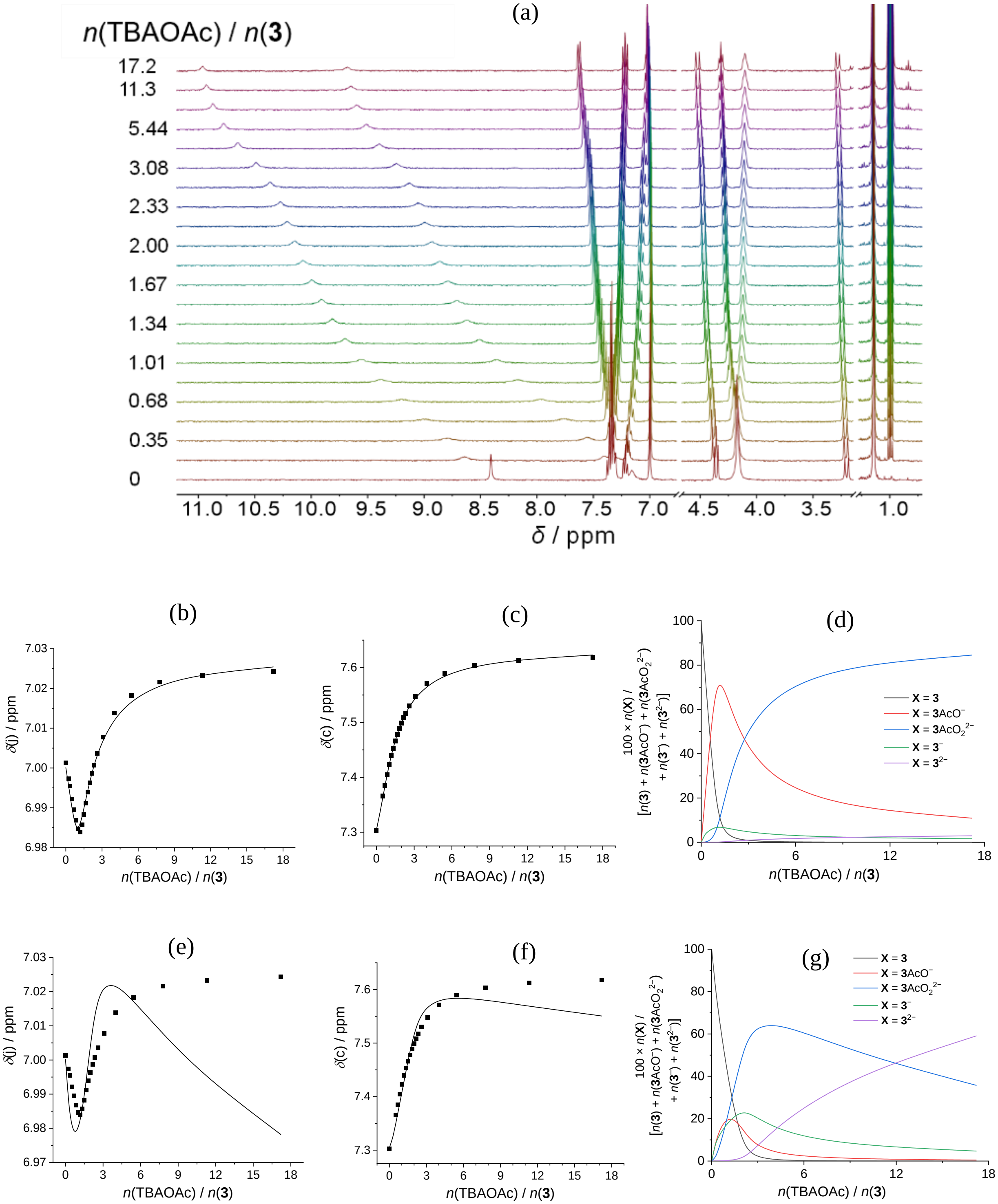

Figure S60. a) ^{1}H NMR spectroscopy titration of **3** (c = 6.02×10^{-4} mol dm^{-3}, V_0 = 500 µL) with TBAOAc (c = 1.42×10^{-2} mol dm^{-3}) in CD_3CN at 25 °C. b), c) Experimental (■), and calculated (—; based on model E, see Table S21) chemical shifts for selected nuclei. d) Distribution of **3** and its complexes with acetate during the titration of **3** with TBAOAc according to model E. e), f) Experimental (■), and calculated (—; based on model D, see Table S21) chemical shifts for selected nuclei. g) Distribution of **3** and its complexes with acetate during the titration of **3** with TBAOAc according to model D.

Table S22. Calculated ^1H NMR chemical shifts (in ppm) for **3** and its complexes with AcO^- in CD_3CN at 25 °C based on model D (Table S21Table S21). Assignment of protons is depicted in Figure S1. Left/right assignments (= downfield/upfield, respectively) refer to the position of signal in NMR spectrum when assigning pair of similar protons.

H	**3**	**3**AcO^-	**3**AcO_2^{2-}
c	7.4892	7.4431	7.6576
d	7.1839	7.2753	7.2121
e	7.013	7.111	7.0023
j	6.9276	6.9741	7.0357
h/i-left	4.4767	4.47	4.5504
f	4.3446	4.2637	4.3279
g	4.1341	4.1247	4.0971
h/i-right	3.2237	3.2691	3.3013
k	1.1323	1.1392	1.1562

Table S23. Calculated ^1H NMR chemical shifts (in ppm) for **3** and its complexes with AcO^- in CD_3CN at 25 °C based on model E (Table S21). Assignment of protons is depicted in Figure S1. Left/right assignments (= downfield/upfield, respectively) refer to the position of signal in NMR spectrum when assigning pair of similar protons.

H	**3**	**3**AcO^-	**3**AcO_2^{2-}
c	7.4892	7.4571	7.6898
d	7.1839	7.221	7.2592
e	7.013	7.0549	7.0577
j	6.9276	6.8267	7.0596
h/i-left	4.4767	4.3215	4.5854
f	4.3446	4.3848	4.3042
g	4.1341	4.1248	4.1003
h/i-right	3.2237	3.325	3.3159
k	1.1323	1.1069	1.1605

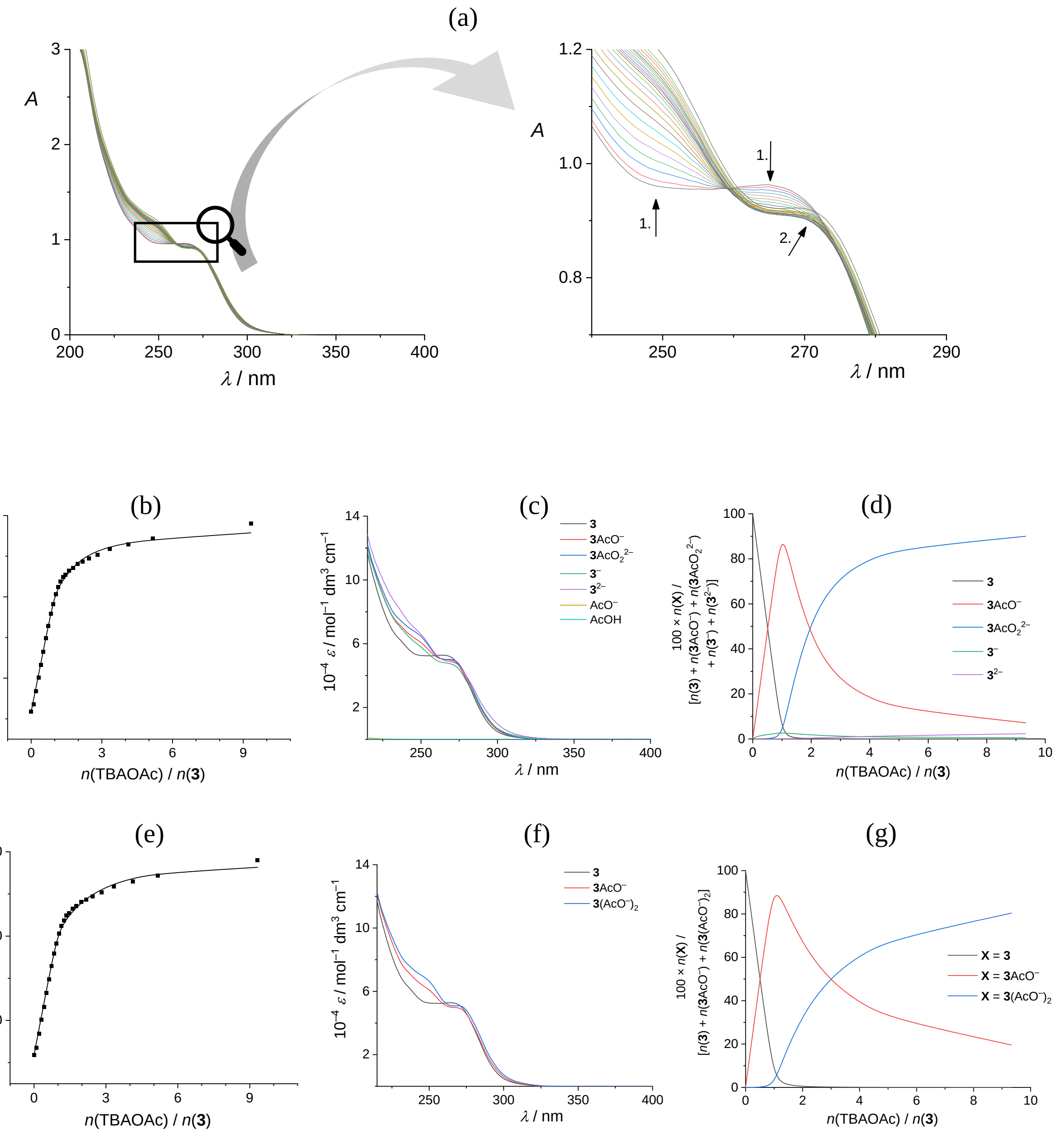

Figure S61. a) Spectrophotometric titration of **3** (c = 1.82×10^{-5} mol dm^{-3}, V_0 = 2.3 mL) with TBAOAc (c = 4.37×10^{-4} mol dm^{-3}) in acetonitrile. l = 1 cm; ϑ = (25.0 ± 0.1) °C. b) Dependence of absorbance at 250 nm on n(TBAOAc) / n(**3**) ratio. Experimental (■), calculated (—; based on model E, see Table S21). c) Characteristic UV spectra of **3** and its complexes with AcO$^-$ based on model E. d) Distribution of **3** and its complexes with acetate during the titration of **3** with TBAOAc based on model E. e) Dependence of absorbance at 250 nm on n(TBAOAc) / n(**3**) ratio. Experimental (■), calculated (—; based on model D, see Table S21). f) Characteristic UV spectra of **3** and its complexes with AcO$^-$ based on model D. g) Distribution of **3** and its complexes with acetate during the titration of **3** with TBAOAc based on model D.

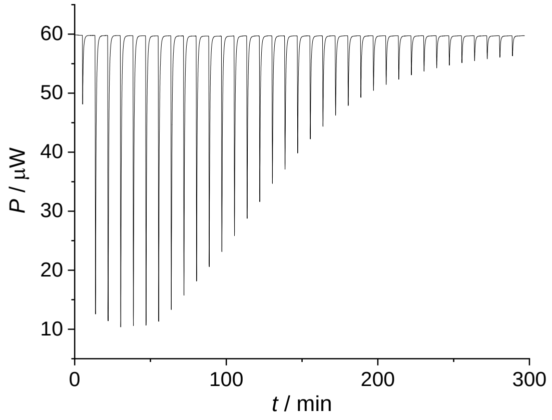

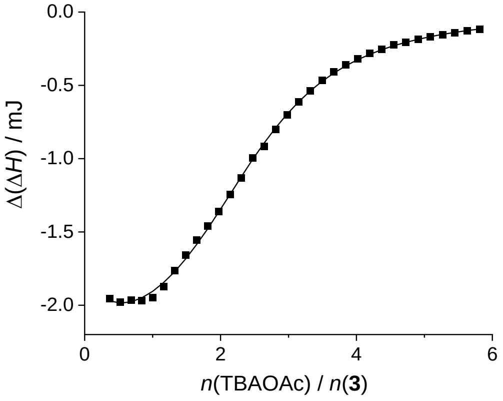

(c)

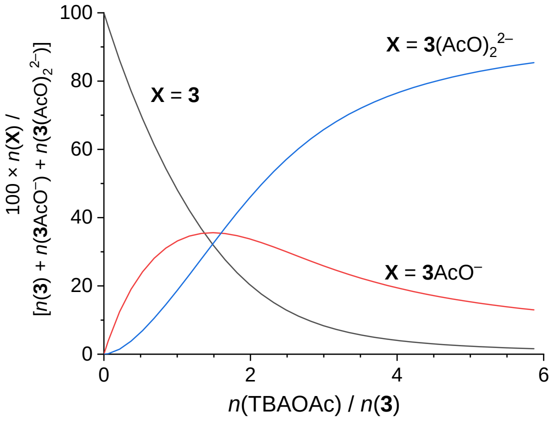

Figure S62. a) Microcalorimetric titration of **3** ($c = 3.95\times10^{-4}$ mol dm^{-3}, $V_0 = 1.43$ mL) with TBAOAc ($c = 1.09\times10^{-2}$ mol dm^{-3}) in acetonitrile at 25 °C. b) Dependence of successive enthalpy change on *n*(TBAOAc) / *n*(**3**) ratio: ■ experimental; — calculated (based on model C, see Table S21). c) Distribution of **3** and its complexes with acetate during the titration of **3** with TBAOAc.

Benzoate

*Complexation of benzoate with **1***

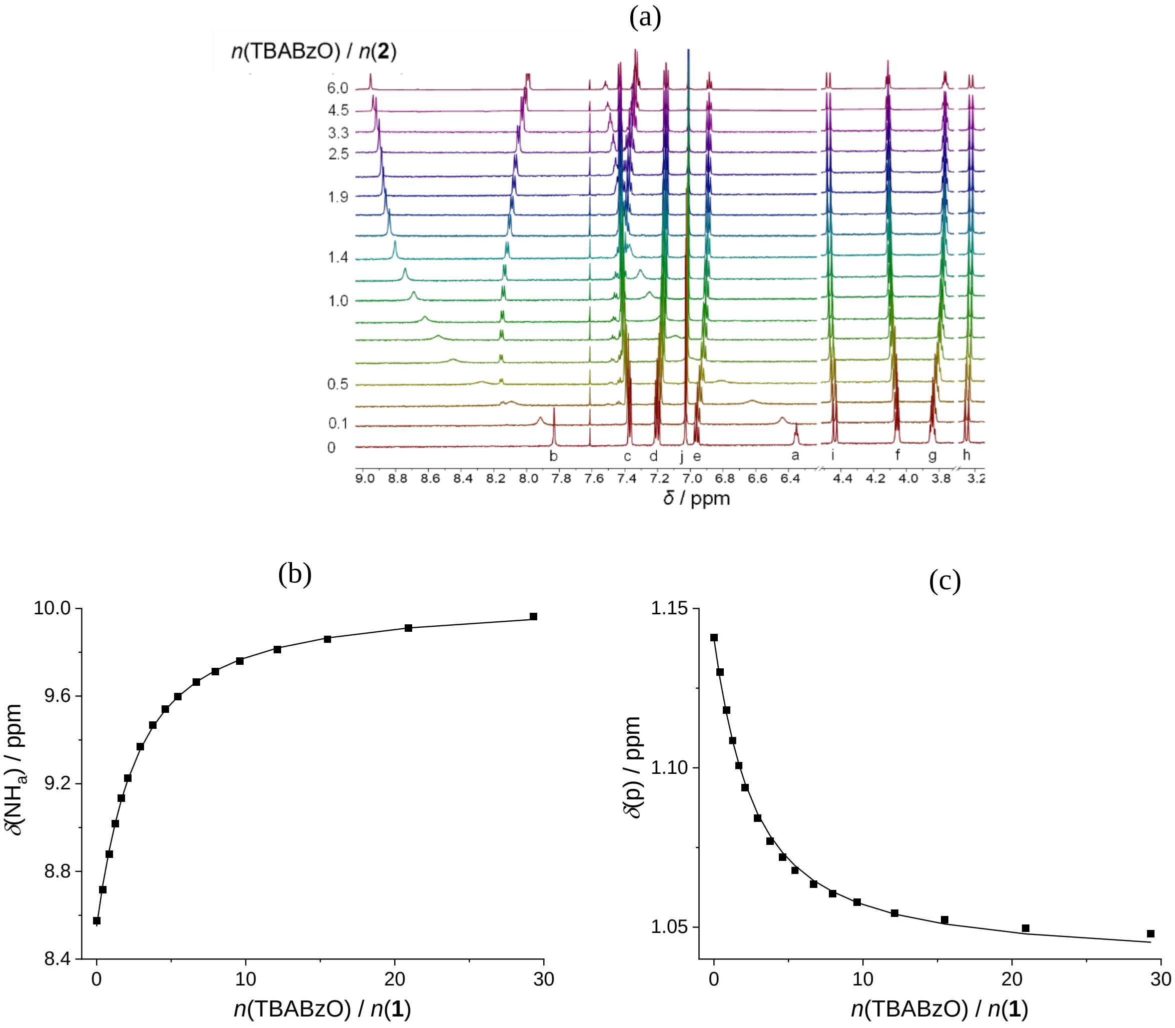

Figure S63. a) ^{1}H NMR spectroscopy titration of **1** (c = 6.72×10^{-4} mol dm^{-3}, V_0 = 500 µL) with TBABzO (c = 0.141 mol dm^{-3}) in CD_3CN at 25 °C. b), c) Experimental (■), and calculated (—) chemical shifts for selected nuclei at **1**.

Table S24. Calculated ^{1}H NMR chemical shifts (in ppm) for **1** and its complex with BzO^- in CD_3CN at 25 °C. Assignment of protons is depicted in Figure S5. Left/right assignments (= downfield/upfield, respectively) refer to the position of signal in NMR spectrum when assigning pairs of similar protons.

H	**1**	**1**BzO^-
NH-a	8.5516	10.0666
NH-b	6.9933	8.4021
e	6.9506	6.8783
n	4.6133	4.6877
g	3.788	3.9019
o-left	3.4372	3.2828
o-right	3.0643	3.3558
p-left	1.1405	1.0373

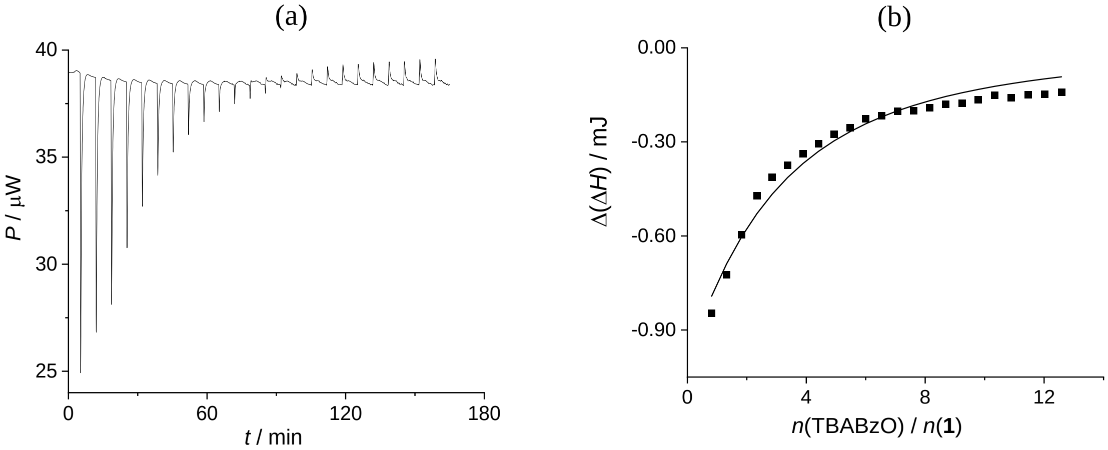

Figure S64. a) Microcalorimetric titration of **1** ($c = 2.26\times10^{-4}$ mol dm^{-3}, $V_0 = 1.432$ mL) with TBABzO ($c = 2.02\times10^{-2}$ mol dm^{-3}) in acetonitrile at 25 °C; b) Dependence of successive enthalpy change on *n*(TBABzO) / *n*(**1**) ratio. ■ Experimental; — calculated.

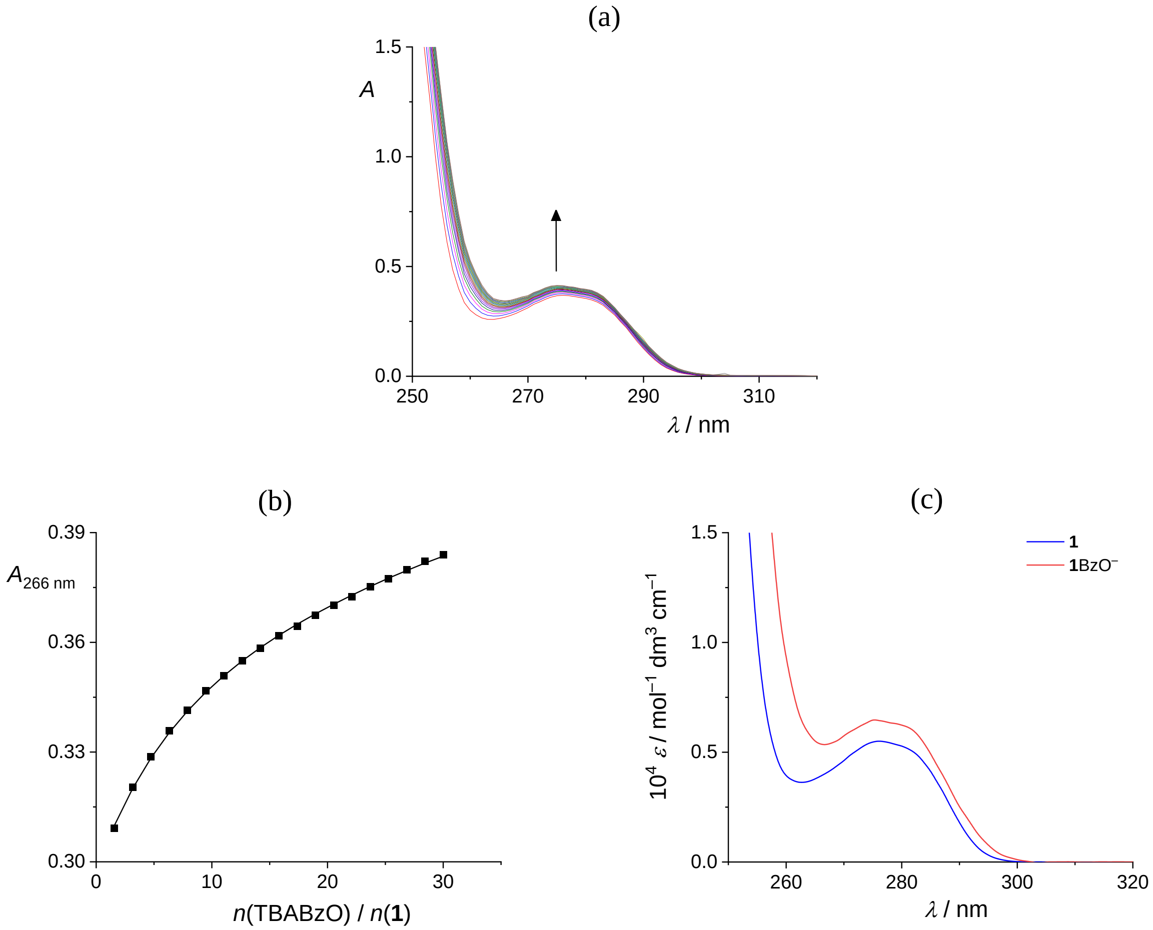

Figure S65. a) Spectrophotometric titration of **1** (c = 6.52×10^{-5} mol dm^{-3}, V_0 = 2.3 mL) with TBABzO (c = 1.58×10^{-2} mol dm^{-3}) in acetonitrile. l = 1 cm; ϑ = (25.0 ± 0.1) °C. The spectra are corrected for benzoate absorption and dilution. b) Dependence of absorbance at 266 nm on n(TBABzO) / n(**1**) ratio. ■ experimental; — calculated, c) Characteristic UV/Vis spectra of **1** and its benzoate complex.

Complexation of benzoate with ***2***

(a)

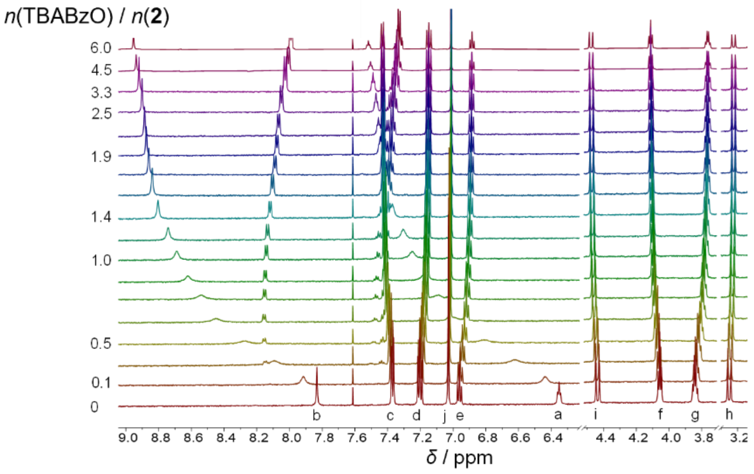

(b)

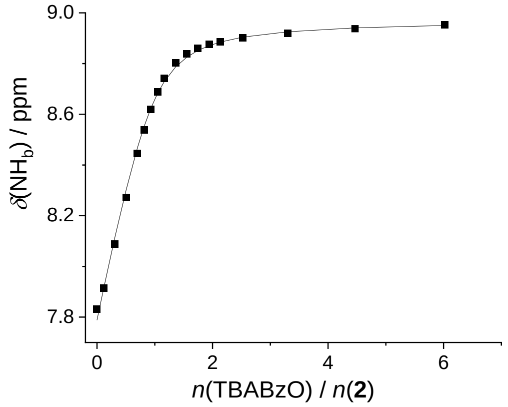

(c)

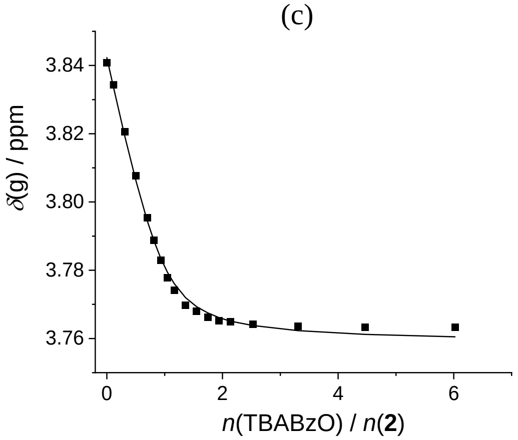

Figure S66. a) ^{1}H NMR spectroscopy titration of **2** (c = 7.09×10^{-5} mol dm^{-3}, V_0 = 500 µL) with TBABzO (c = 1.38×10^{-3} mol dm^{-3}) in CD_3CN at 25 °C. b), c) Experimental (■), and calculated (—) chemical shifts for selected nuclei at **2**.

Table S25. Calculated ^{1}H NMR chemical shifts (in ppm) for **2** and **2**BzO^{-} in CD_3CN at 25 °C. Assignment of protons is depicted in Figure S5.

H	**2**	**2**BzO^{-}
a	6.3062	7.5488
b	7.7886	8.9785
c	7.3682	7.4334
d	7.2056	7.1461
e	6.9646	6.8823
f	4.0541	4.1144
g	3.8423	3.7585
h	3.2521	3.2226
i	4.4342	4.4769

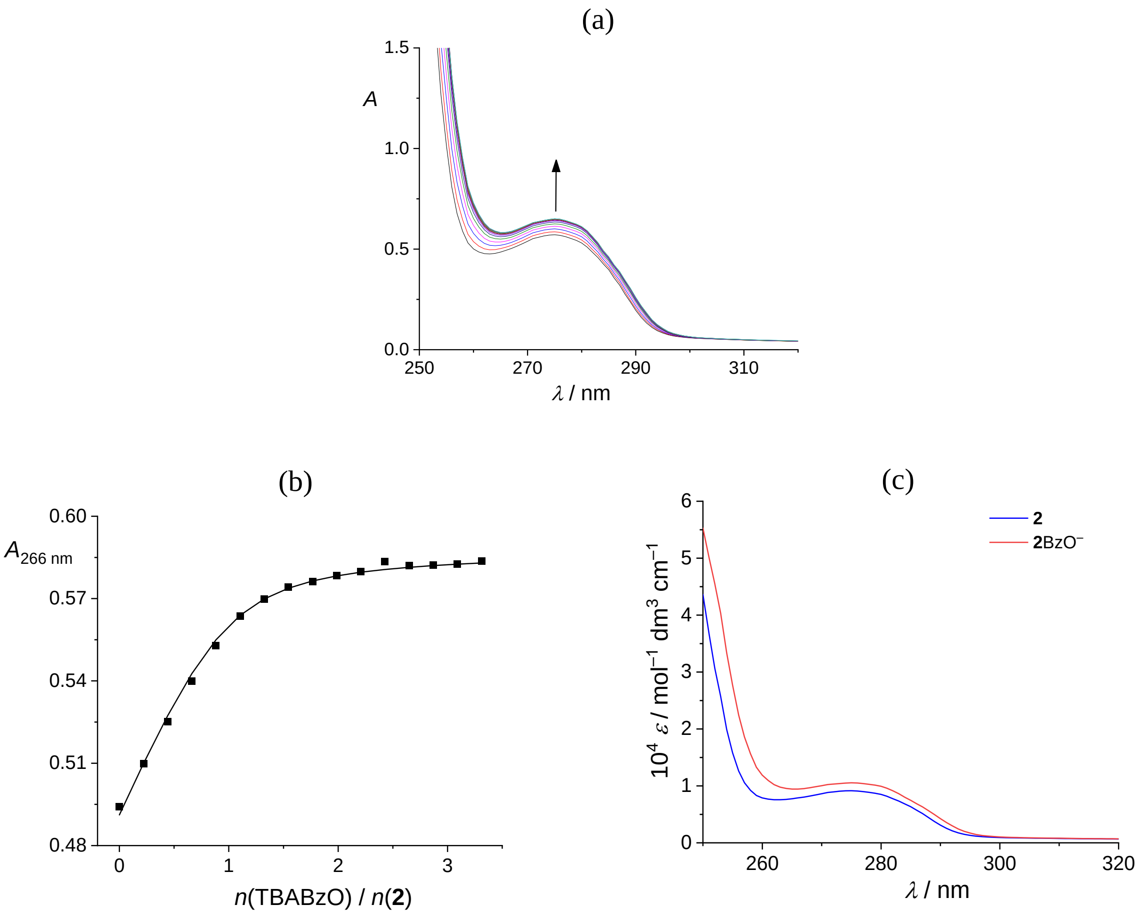

Figure S67. a) Spectrophotometric titration of **2** ($c = 6.22\times10^{-5}$ mol dm^{-3}, $V_0 = 2.3$ mL) with TBABzO ($c = 1.58\times10^{-3}$ mol dm^{-3}) in acetonitrile. $l = 1$ cm; $\vartheta = (25.0 \pm 0.1)$ °C. The spectra are corrected for benzoate absorption and dilution. b) Dependence of absorbance at 266 nm on n(TBABzO) / n(**2**) ratio. ■ Experimental; — calculated, c) Characteristic UV/Vis spectra of **2** and its benzoate complex.

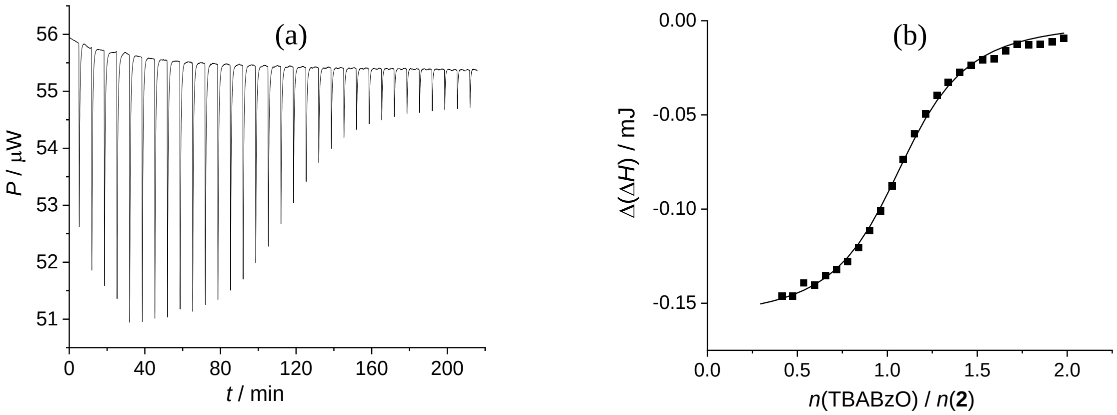

Figure S68. a) Microcalorimetric titration of **2** ($c = 6.67\times10^{-5}$ mol dm^{-3}, $V_0 = 1.430$ mL) with TBABzO ($c = 1.12\times10^{-3}$ mol dm^{-3}) in acetonitrile at 25 °C; b) Dependence of successive enthalpy change on n(TBABzO) / n(**2**) ratio. ■ experimental; — calculated.

(a)

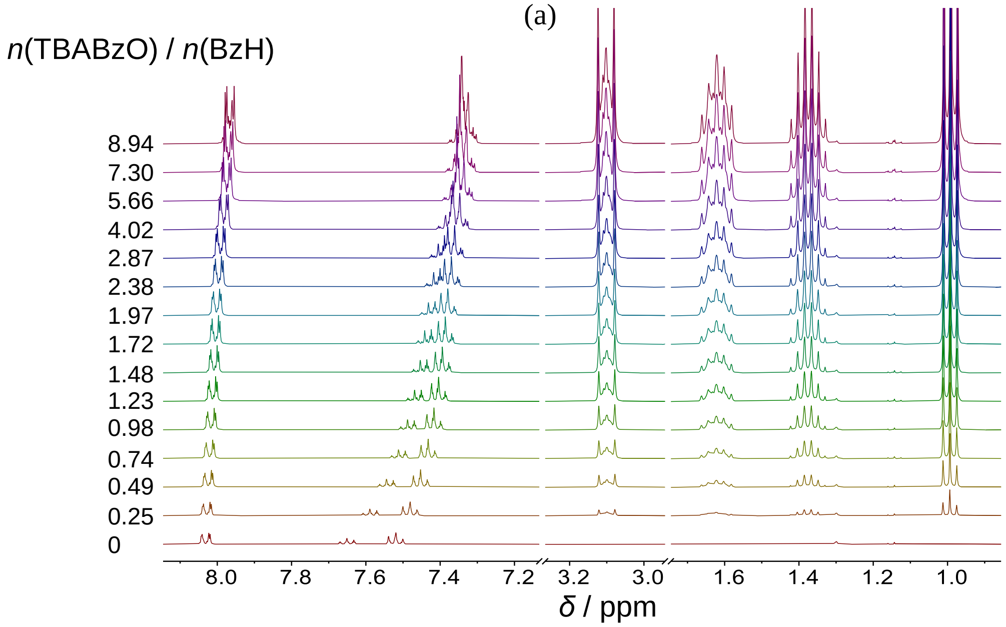

(b)

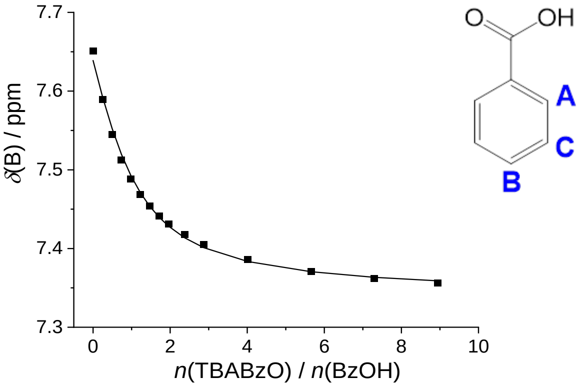

Figure S69. a) ^{1}H NMR spectroscopy titration of benzoic acid ($c = 9.45 \times 10^{-4}$ mol dm^{-3}, $V_0 = 515$ µL) with TBABzO ($c = 1.60 \times 10^{-2}$ mol dm^{-3}) in CD_3CN at 25 °C. b) Experimental (■) and calculated (—) chemical shifts for nucleus B at benzoic acid/benzoate (fast exchange).

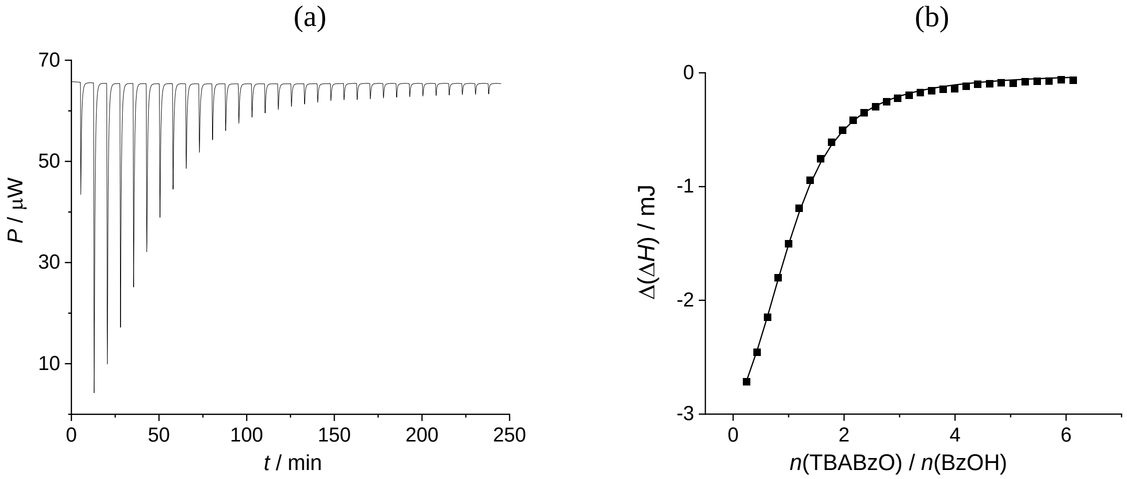

Figure S70. a) Microcalorimetric titration of BzOH (c = 5.05×10^{-4} mol dm^{-3}, V_0 = 1.45 mL) with TBABzO (c = 1.51×10^{-2} mol dm^{-3}) in acetonitrile at 25 °C. b) Dependence of successive enthalpy change on n(TBABzO) / n(BzO) ratio: ■ experimental; — calculated.

*Complexation of benzoate with **3***

(a)

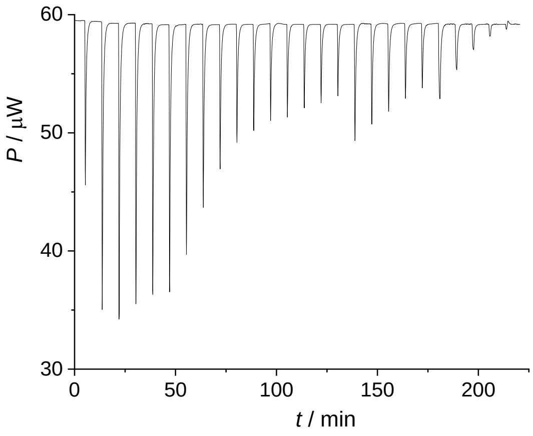

(b)

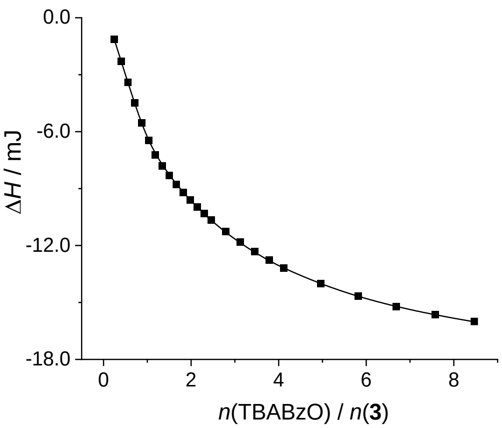

(c)

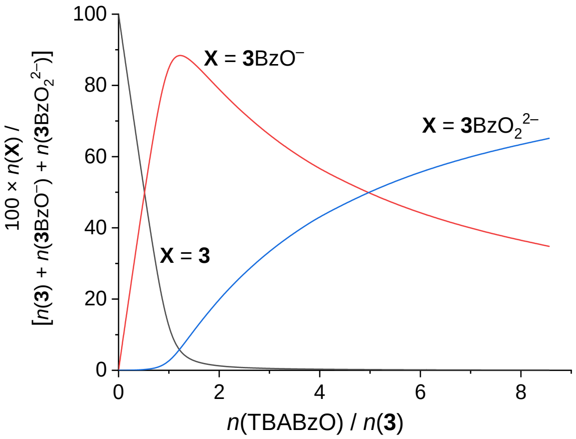

Figure S71. a) Microcalorimetric titration of **3** ($c = 2.29\times10^{-4}$ mol dm^{-3}, $V_0 = 1.45$ mL) with TBABzO ($c = 1.02\times10^{-2}$ mol dm^{-3}) in acetonitrile at 25 °C. b) Dependence of cumulative enthalpy change on *n*(TBABzO) / *n*(**3**) ratio: ■ experimental; — calculated (based on model A, see Table S26). The successive volumes of titrant additions follow this order: 1. 3 μL (not shown), 2. – 16. 5 μL, 17. – 21. 10 μL, 22. – 28. 25 μL. c) Distribution of **3** and its complexes with benzoate during the titration of **3** with TBABzO.

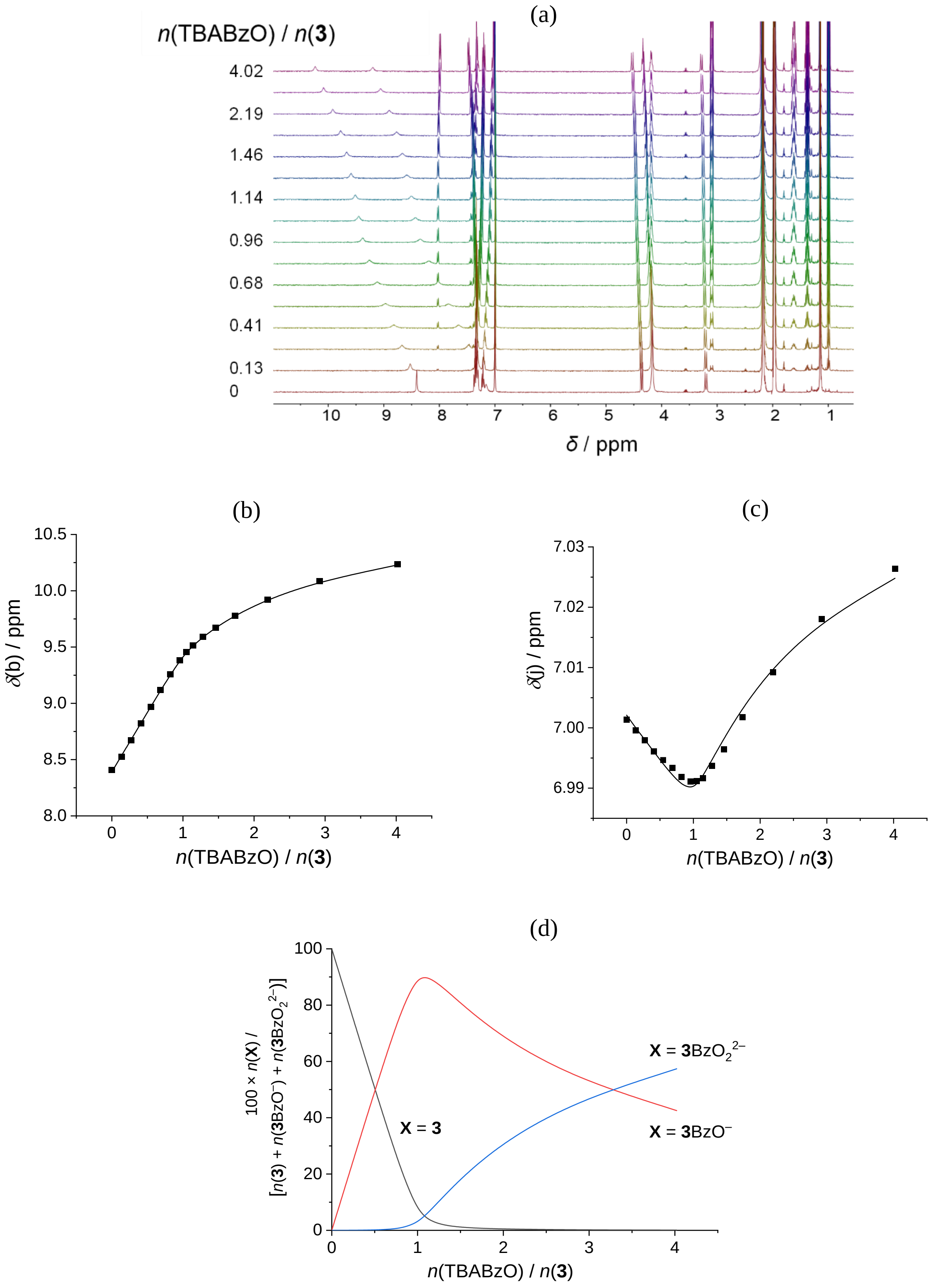

Figure S72. a) ^{1}H NMR spectroscopy titration of **3** (c = 6.87 ×10^{-4} mol dm^{-3}, V_0 = 500 µL) with TBABzO (c = 6.23×10^{-3} mol dm^{-3}) in CD_3CN at 25 °C. b), c) Experimental (■), and calculated (—; based on model A, see Table S26) chemical shifts for selected nuclei at **3**. d) Distribution of **3** and its complexes with benzoate during the titration of **3** with TBABzO.

Table S26. Two models used for fitting data obtained from titrations of **3** with TBABzO. Here are presented real values of cumulative stability constants (β), whereas in HYPNMR program reduced values were used according to the procedure described in chapter: Dealing with high values of protonation constants in HYPNMR.

**Model A**		
reaction	log β	Value fixed or refined during fitting?
$\mathbf{3} + BzO^- \rightleftarrows \mathbf{3}BzO^-$	5.59	fixed
$\mathbf{3} + 2\,BzO^- \rightleftarrows \mathbf{3}BzO_2^{2-}$	8.66	refined
**Model B**		
reaction	log β	Value fixed or refined during fitting?
$\mathbf{3}^{2-} + H^+ \rightleftarrows \mathbf{3}^-$	24.28	fixed
$\mathbf{3}^{2-} + 2\,H^+ \rightleftarrows \mathbf{3}$	47.12	fixed
$H^+ + BzO^- \rightleftarrows BzOH$	21.5	fixed
$H^+ + 2\,BzO^- \rightleftarrows BzOH{\cdot}BzO^-$	24.8	fixed
$MeCN \rightleftarrows MeCN^- + H^+$	−39	fixed
$\mathbf{3}^{2-} + 2\,H^+ + BzO^- \rightleftarrows \mathbf{3}BzO^-$	52.17	fixed
$\mathbf{3}^{2-} + 2\,H^+ + 2\,BzO^- \rightleftarrows \mathbf{3}BzO_2^{2-}$	55.78	refined

Table S27. Calculated ^{1}H NMR chemical shifts (in ppm) for **3** and its complexes with BzO^- in CD_3CN at 25 °C based on model A (Table S26). Assignment of protons is depicted in Figure S1. Left/right assignments (= downfield/upfield, respectively) refer to the position of signal in NMR spectrum when assigning pair of similar protons.

H	**3**	**3**BzO^-	**3**BzO_2^{2-}
b	8.391	9.4555	10.8067
c	7.305	7.3633	7.5348
d	7.3595	7.2316	7.1732
e	7.2193	7.0793	6.9951
a	7.1302	8.4496	9.7626
j	7.0021	6.987	7.0528
h/i-left	4.3722	4.4682	4.5988
f	4.1587	4.2574	4.3943
g	4.1728	4.1919	4.1914
h/i-right	3.2165	3.2603	3.3279
k	1.1439	1.1431	1.1652

(a) (b)

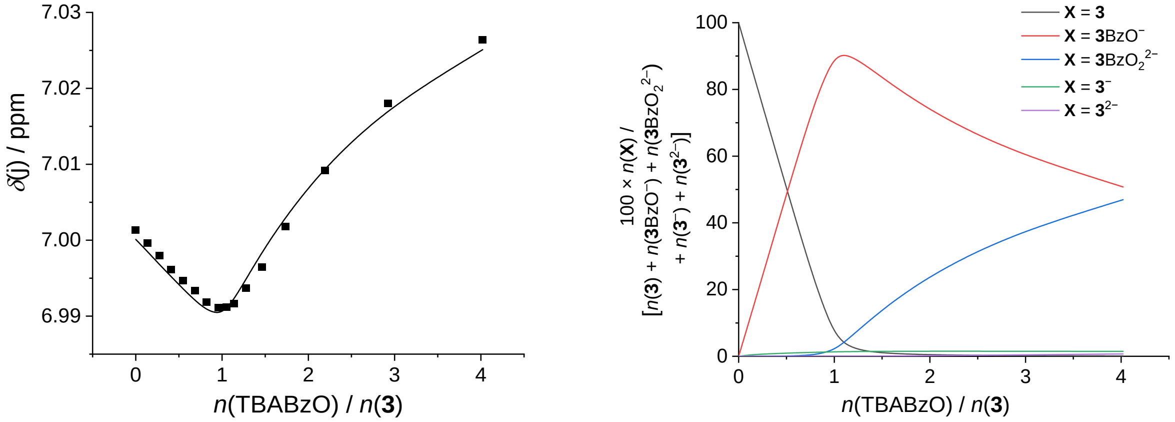

Figure S73. ^{1}H NMR spectroscopy titration of **3** (c = 6.87 ×10^{-4} mol dm^{-3}, V_0 = 500 μL) with TBABzO (c = 6.23×10^{-3} mol dm^{-3}) in CD_3CN at 25 °C. a) Experimental (■), and calculated (—; based on model B, see Table S26) chemical shifts for proton "j" at **3**. b) Distribution of **3** and its complexes with benzoate during the titration of **3** with TBABzO.

Table S28. Calculated ^{1}H NMR chemical shifts (in ppm) for **3** and its complexes with BzO^- in CD_3CN at 25 °C based on model B (Table S26). Assignment of protons is depicted in Figure S1. Left/right assignments (= downfield/upfield, respectively) refer to the position of signal in NMR spectrum when assigning pair of similar protons.

H	**3**	**3**BzO^-	**3**BzO_2^{2-}
c	7.4892	7.3657	7.5755
d	7.1839	7.2311	7.1623
e	7.013	7.0781	6.9801
j	6.9276	6.9876	7.0678
h/i-left	3.3925	4.4792	4.6561
f	4.3446	4.2586	4.4267
g	5.6703	4.1389	4.1161
h/i-right	3.117	3.2649	3.3506
k	1.1323	1.1433	1.1702

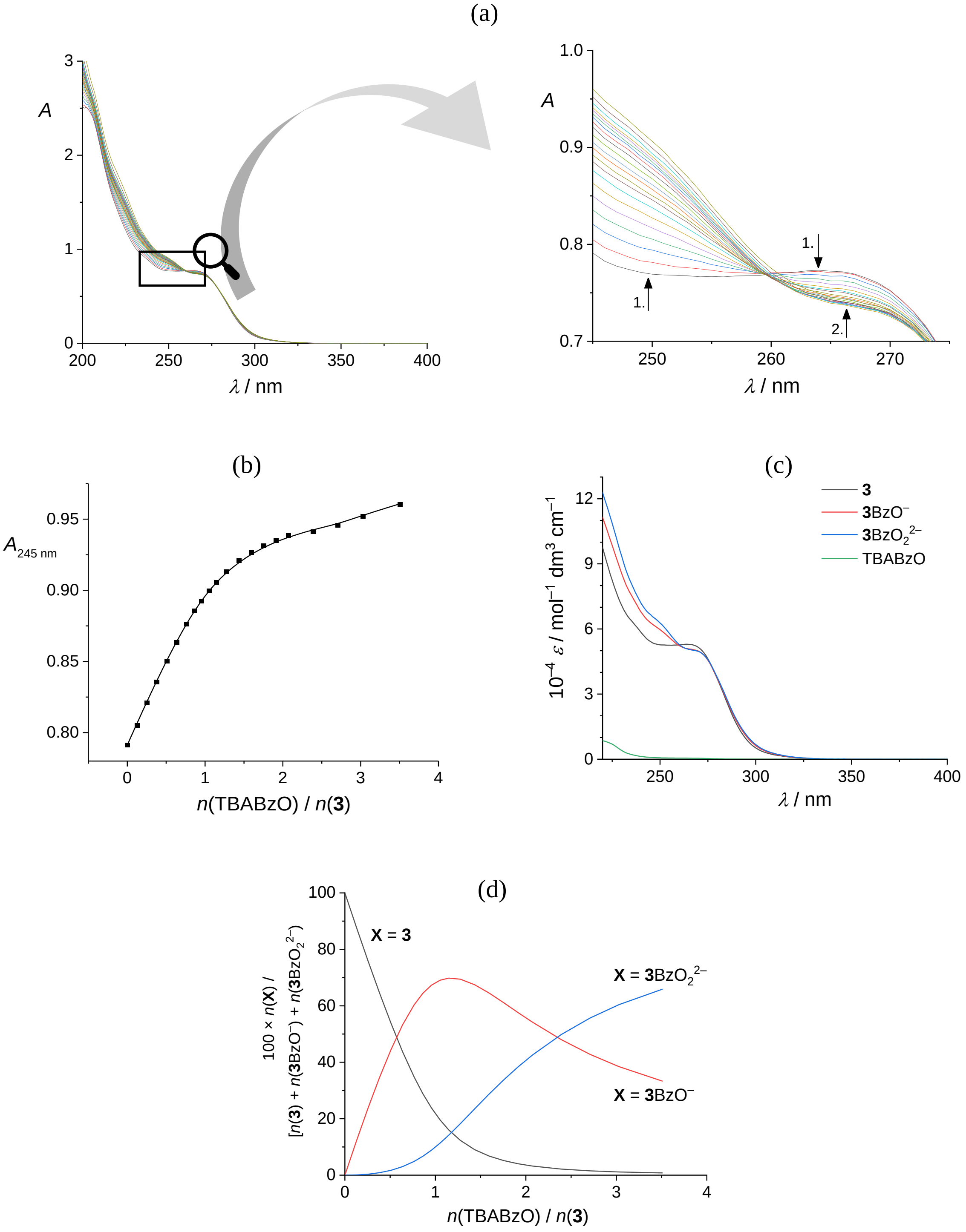

Figure S74. a) Spectrophotometric titration of **3** ($c = 1.46\times10^{-5}$ mol dm^{-3}, $V_0 = 2.3$ mL) with TBABzO ($c = 2.14\times10^{-4}$ mol dm^{-3}) in acetonitrile. $l = 1$ cm; $\vartheta = (25.0 \pm 0.1)$ °C. The spectra are corrected for the absorption of benzoate and for the dilution of **3**. b) Dependence of absorbance at 245 nm on n(TBABzO) / n(**3**) ratio. Experimental (■), calculated (—; based on model B, see Table S26). c) Characteristic UV spectra of **3** and its complexes with BzO^-. d) Distribution of **3** and its complexes with benzoate during the titration of **3** with TBABzO.

Additional proof of the experimentally obtained values for the complexation of benzoate with **3** emerged from the results of the titration of **3**$^{-}$ with BzOH followed by TBABzO, which was composed of three processes: protonation of calixarene, homoconjugation and complexation of benzoate:

(a)

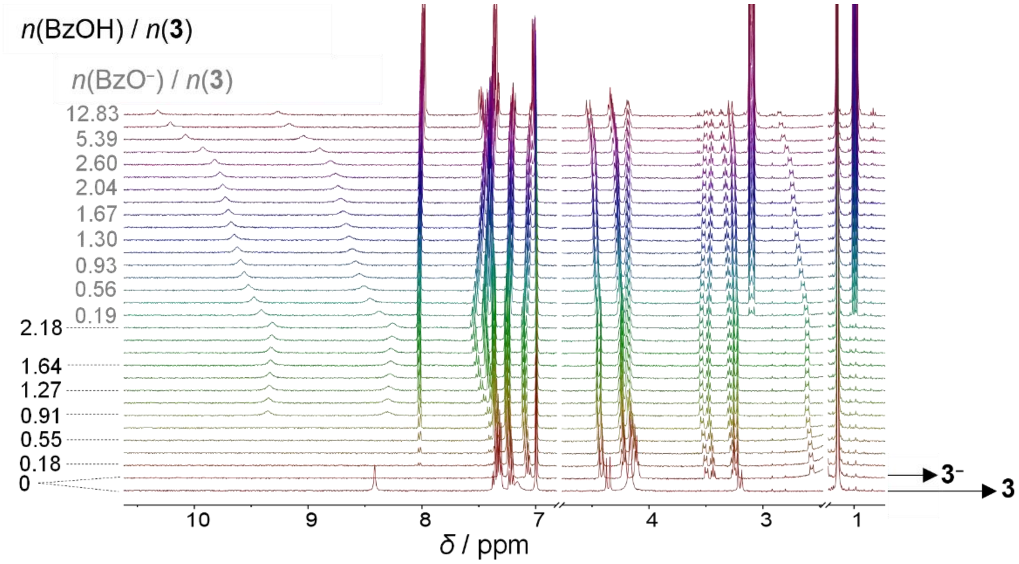

(b)

(c)

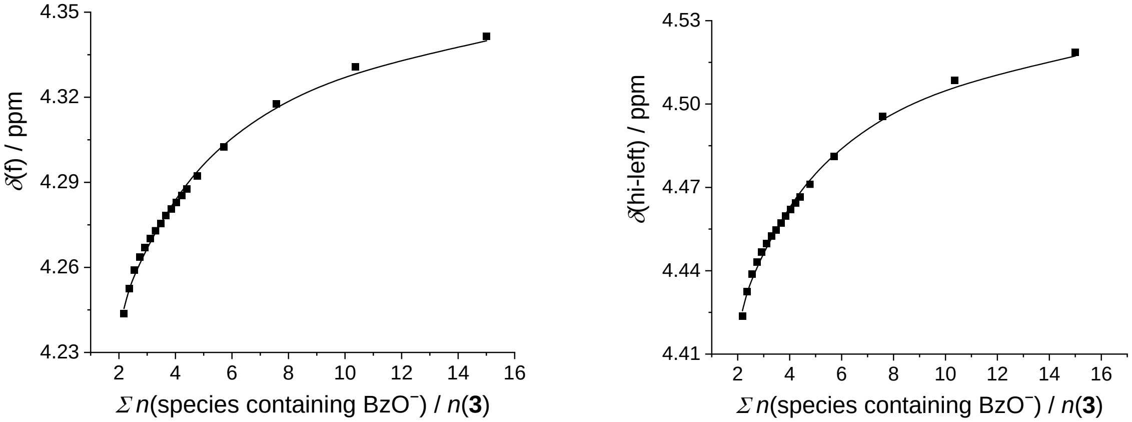

(d)

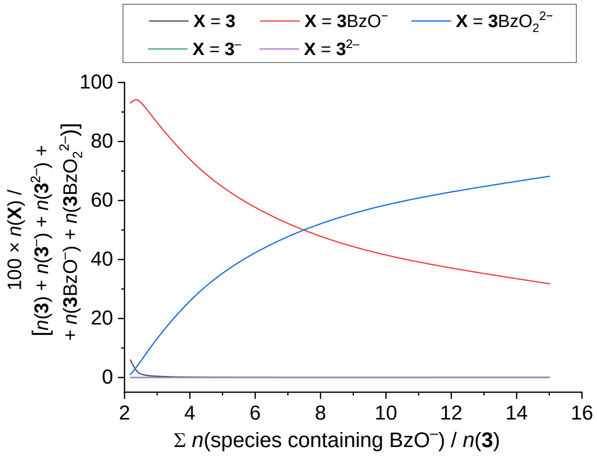

(e)

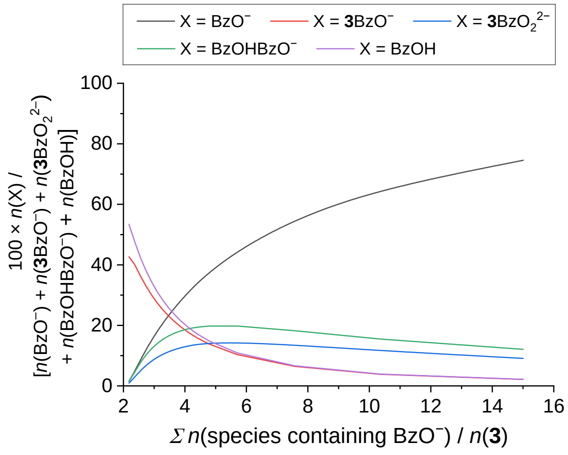

(f)

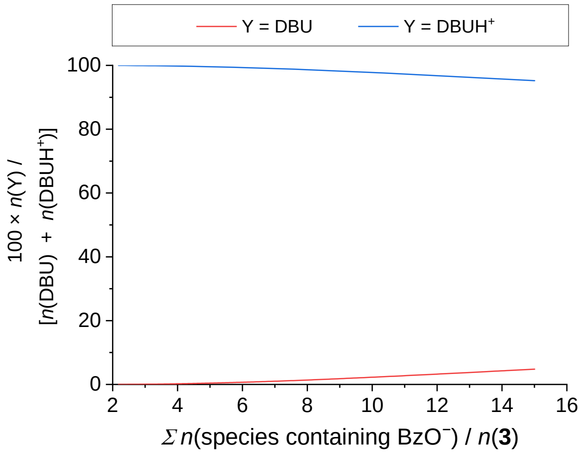

Figure S75. a) ^{1}H NMR titration of **3**$^-$($c = 5.96\times10^{-4}$ mol dm^{-3}, $V_0 = 450$ µL, deprotonation executed with the addition of 1 molEq DBU base in 35 µL) first with BzOH ($c = 3.25\times10^{-2}$ mol dm^{-3}), and then with TBABzO ($c = 9.96\times10^{-3}$ mol dm^{-3}) in CD_3CN at 25 °C. b), c) Experimental (■), and calculated (—; based on model X, see Table S29) chemical shifts for selected nuclei at **3**. Distribution of d) calixarene **3**, e) benzoate, and f) DBU between all possible species containing them during the titration.

Table S29. Model X used for fitting data obtained from the titration depicted in Figure S75. Here are presented real values of log β, whereas in HYPNMR program reduced values were used according to the procedure described in chapter: Dealing with high values of protonation constants in HYPNMR. The values of characteristic chemical shifts for **3**, **3**$^{-}$ and **3**$^{2-}$ were fixed based on the results obtained from the NMR titration of **3** with DBU (Figure S8).

**Model X**		
reaction	log β	Value fixed or refined during fitting?
$\mathbf{3}^{2-} + H^+ \rightleftarrows \mathbf{3}^-$	24.28	fixed
$\mathbf{3}^{2-} + 2\,H^+ \rightleftarrows \mathbf{3}$	47.12	fixed
$BzO^- + H^+ \rightleftarrows BzOH$	21.5	fixed
$DBU + H^+ \rightleftarrows DBUH^+$	24.34	fixed
$2\,BzO^- + H^+ \rightleftarrows BzOH{\cdot}BzO^-$	24.4	fixed
$MeCN \rightleftarrows MeCN^- + H^+$	−39	fixed
$\mathbf{3}^{2-} + 2\,H^+ + BzO^- \rightleftarrows \mathbf{3}{\cdot}BzO^-$	53.07	fixed
$\mathbf{3}^{2-} + 2\,H^+ + 2\,BzO^- \rightleftarrows \mathbf{3}{\cdot}(BzO^-)_2$	55.85	refined

References

(1) Cvetnić, M.; Cindro, N.; Topić, E.; Bregović, N.; Tomišić, V. Supramolecular Handshakes: Characterization of Urea-Carboxylate Interactions Within Calixarene Frameworks. *ChemPlusChem* **2024**, e202400130. https://doi.org/10.1002/cplu.202400130.

(2) Himmel, D.; Goll, S. K.; Leito, I.; Krossing, I. A Unified PH Scale for All Phases. *Angew. Chem., Int. Ed.* **2010**, *49* (38), 6885–6888. https://doi.org/10.1002/anie.201000252.

(3) Schwesinger, R.; Schlemper, H.; Hasenfratz, C.; Willaredt, J.; Dambacher, T.; Breuer, T.; Ottaway, C.; Fletschinger, M.; Boele, J.; Fritz, H.; Putzas, D.; Rotter, H. W.; Bordwell, F. G.; Satish, A. V.; Ji, G. Z.; Peters, E. M.; Peters, K.; Von Schnering, H. G.; Walz, L. Extremely Strong, Uncharged Auxiliary Bases; Monomeric and Polymer-Supported Polyaminophosphazenes (P2-P5). *Liebigs Ann.* **1996**, *1996* (7), 1055–1081. https://doi.org/10.1002/jlac.199619960705.

(4) Kaljurand, I.; Kütt, A.; Sooväli, L.; Rodima, T.; Mäemets, V.; Leito, I.; Koppel, I. A. Extension of the Self-Consistent Spectrophotometric Basicity Scale in Acetonitrile to a Full Span of 28 PKa Units: Unification of Different Basicity Scales. *J. Org. Chem.* **2005**, *70* (3), 1019–1028. https://doi.org/10.1021/jo048252w.

(5) Tshepelevitsh, S.; Kütt, A.; Lõkov, M.; Kaljurand, I.; Saame, J.; Heering, A.; Plieger, P. G.; Vianello, R.; Leito, I. On the Basicity of Organic Bases in Different Media. *Eur. J. Org. Chem.* **2019**, *2019* (40), 6735–6748. https://doi.org/10.1002/ejoc.201900956.

(6) Horvat, G.; Tarana, S.; Vidović, N.; Cindro, N.; Speranza, G.; Tomišić, V. Thermodynamic and MD Studies of Anion Complexation by Cyclopentaleucine in Acetonitrile and Dimethyl Sulfoxide. *J. Mol. Liq.* **2021**, *340*, 116848. https://doi.org/10.1016/j.molliq.2021.116848.

(7) Izutsu, K. *Electrochemistry in Nonaqueous Solutions*; Wiley-VCH Verlag GmbH & Co. KGaA: Weinheim, 2002.

(8) De Namor, A. F. D.; Shehab, M. Recognition of Biologically and Environmentally Important Phosphate Anions by Calix[4]Pyrrole: Thermodynamic Aspects. *J. Phys. Chem. A* **2004**, *108* (35), 7324–7330. https://doi.org/10.1021/jp031343x.

(9) Danil De Namor, A. F.; Shehab, M.; Abbas, I.; Withams, M. V.; Zvietcovich-Guerra, J. New Insights on Anion Recognition by Isomers of a Calix Pyrrole Derivative. *J. Phys. Chem. B* **2006**, *110* (25), 12653–12659. https://doi.org/10.1021/jp060859o.

(10) Danil De Namor, A. F.; Chaaban, J. K.; Abbas, I. Cation/Anion Recognition by a Partially Substituted Lower Rim Calix[4]Arene Hydroxyamide, a Ditopic Receptor. *J. Phys. Chem. A* **2006**, *110* (31), 9575–9584. https://doi.org/10.1021/jp062154s.
